# Alkaloids from single skins of the Argentinian toad *Melanophryniscus rubriventris* (ANURA, BUFONIDAE): An unexpected variability in alkaloid profiles and a profusion of new structures

**DOI:** 10.1186/2193-1801-1-51

**Published:** 2012-11-23

**Authors:** H Martin Garraffo, Nirina R Andriamaharavo, Marcos Vaira, María F Quiroga, Cecilia Heit, Thomas F Spande

**Affiliations:** 1 Laboratory of Bioorganic Chemistry, NIDDK, NIH, DHHS, 20892 Bethesda, MD USA; 2 CIBA, Facultad de Ingeniería, Universidad Nacional de Jujuy, Gorriti 237, 4600 Jujuy, Argentina; 3 Instituto de Bio y Geociencias del NOA, Universidad Nacional de Salta, Mendoza 2, Salta, Argentina; 4 Facultad de Ciencias Agrarias, Universidad Nacional de Jujuy, Alberdi 47, Jujuy, Argentina; 5 Laboratorio de Análisis de Residuos y Trazas (LAnaRT), Zorrilla de San Martín, 2170 Jujuy, Argentina

**Keywords:** Alkaloids, Gas chromatography–mass spectrometry, *Melanophryniscus* Toads, Reproduction, Sequestration

## Abstract

GC-MS analysis of single-skins of ten *Melanophryniscus rubriventris* toads (five collections of two toads each) captured during their breeding season in NW Argentina has revealed a total of 127 alkaloids of which 56 had not been previously detected in any frog or toad. Included among these new alkaloids are 23 new diastereomers of previously reported alkaloids. What is particularly distinguishing about the alkaloid profiles of these ten collections is the occurrence of many of the alkaloids, whether known or new to us, in only one of the ten skins sampled, despite two skins being obtained from each breeding site of the five populations. Many of the alkaloids are of classes known to have structures with branched-chains (e.g. pumiliotoxins and tricyclic structures) that are considered to derive from dietary mites. A large number of previously reported and new alkaloids are also of unclassified structures. Only a very few 3,5-disubstituted-indolizidine or -pyrrolizidine alkaloids are observed that have a straight-chain carbon skeleton and are likely derived from ant prey. The possible relationship of these collections made during the toad’s brief breeding episodes to sequestration of dietary arthropods and individual alkaloid profiles is discussed.

**Electronic supplementary material:**

The online version of this article (doi:10.1186/2193-1801-1-51) contains supplementary material, which is available to authorized users.

## Introduction

Nine of the 25 currently known species (Frost, 2011) of South American bufonid toads of the genus *Melanophryniscus* have been surveyed for lipophilic alkaloids in skin glands. These alkaloids are considered to provide a defense against predation, particularly during the mainly diurnal breeding episodes of the toads (Santos and Grant 2010a). The toads examined for alkaloids are *M* (Mebs et al. . *atroluteus*^2007^), *M*. *cupreuscapularis* (Daly et al. 2008a), *M* (Mebs et al.. *devincenzii*^2007^), *M*. *klappenbachi* (Daly et al. 2008a), *M* (Daly et al. . *moreirae*^1984^), *M*. *montevidensis* (Garraffo et al. 1993a; Mebs et al. 2005), *M* (Daly et al. . *rubriventris*^2007^), and *M*. *stelzneri* (Garraffo et al. 1993a; Daly et al. 2007). The Brazilian species, *M*. *simplex* has recently been investigated and contains both known and new alkaloids (Grant et al. 2012). A tenth species, *M*. *cambaraensis* from southern Brazil, is reported to have “defensive chemicals” in skin, not proven, however, to be alkaloids (Colombo and Grant, cited in Santos and Grant 2010a).

**Figure 1:**
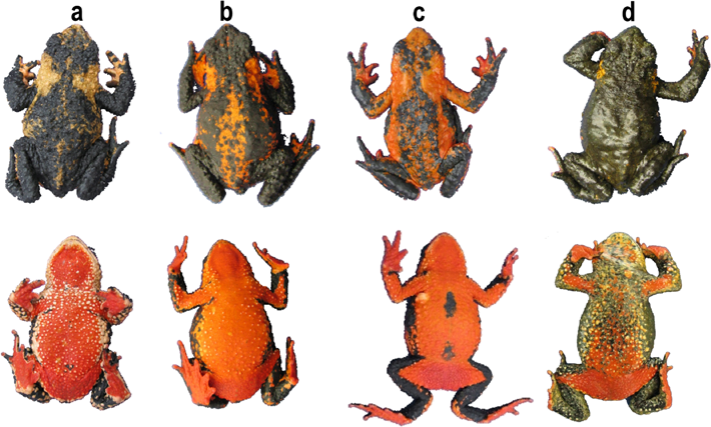
**Photographs showing the bright coloration and patterns typical of*M***. ***rubriventris*toads in Argentina.** Specimens are from: **a**) Huaico Chico, Salta (skins #3, 4); **b**) Tablada, Jujuy (skins #5, 6); **c**) Abra Colorada and **d**) Tiraxi (see Figure 2).

Eighty-one skins of a Uruguayan *Melanophryniscus* toad, *M*. *montevidensis* from six southeastern sites were individually examined and had widely varying amounts of pumiliotoxin ( ranging from none, or scarcely detectible, to as much as 1 mg per toad. Traces of six other PTX alkaloids (Mebs et al. PTX) **251D**^2005^) were also seen as well as traces of indolizidines that were not characterized. From two northern Uruguayan toads, *M*. *atroluteus* and *M*. *devincenzii* was the major alkaloid identified along with five known trace PTX alkaloids and another trace PTX congener (Mebs et al. , PTX **251D**^2007^species that are known to have alkaloids, are reported to have many more alkaloids, ranging from 10–25 alkaloids (Daly et al. ). Skins of the remaining six *Melanophryniscus*^2007^) to as many as 36 (Daly et al. 2008a), all with **251D** and other alkaloids of the PTX class, several di- and tri-substituted indolizidines, disubstituted pyrrolizidines, disubstituted quinolizidines and tricyclic alkaloids. Some classes, considered from their straight-chain structures to derive from ants, were significant in a 1989 collection of *M*. *stelzneri* from Córdoba, Argentina, (Garraffo et al. 1993a) but were scarce in a collection from the same site made ten years later (Daly et al. 2007). Combined skin collections from four populations of *M* likewise reflected temporal variation (Daly et al. . *rubriventris*^2007^, the subject of the current study, is a species of aposematic toads with a snout vent length (SVL) of 32–43 mm and restricted to the NW Argentinian provinces of Salta and Jujuy and temperate interandean valleys of southern Bolivia (Frost ). *Melanophryniscus rubriventris*^2011^). Although the species was first formally described as a toad with a black background and bright orange coloration covering the scapular region and partially the head, dorsum and flanks, with a uniform red ventral coloration (Vellard 1947), subsequent studies on population color variations in Argentina showed that they differed significantly in dorsal and ventral coloration patterns (Vaira 2002). Northern and central populations (Salta and central east of Jujuy province) have individuals with bright uniform dorsum, differing mainly in the extent of black patches, whereas the southern population (southeast of Jujuy province) has toads that predominately showed a more cryptic olive to black dorsal pattern. Concomitant individuals from northern and central populations have a mostly uniform yellow to red belly whereas the southern population has toads with well-demarcated yellow, red and black speckled bellies (see Figure 1).

**Figure 2:**
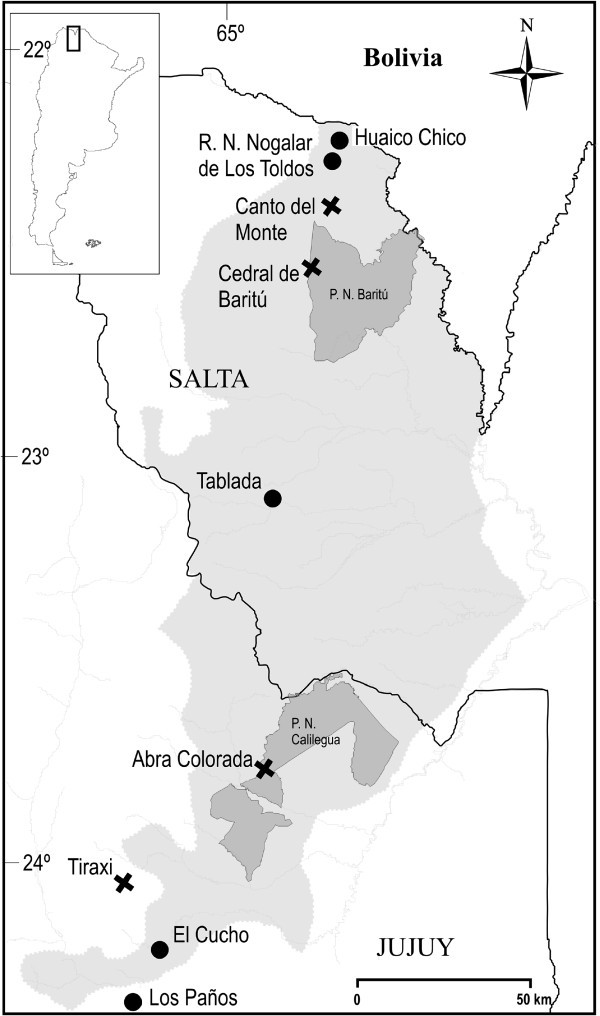
**Map of the collection sites of this study** (**filled circles**) **and the earlier study** (“**x**”) **of Daly et al. 2007.** The larger light grey shaded area is the Las Yungas Biosphere Reserve; the darker grey areas within it are National Parks (P.N.).

Earlier work on four *M*. *rubriventris* multi-skin collections made in 2002 from two sites in Salta province and two in Jujuy province (see Figure 2) indicated all contained various representatives of the PTX class but only two populations (Canto del Monte, Salta and Tiraxi, Jujuy) contained pumiliotoxin **251D**, normally found in *Melanophryniscus* (bufonid) toads and common also to some dendrobatid, *Mantella* (mantellid) and *Pseudophryne* (myobatrachid) frogs. The Tiraxi two-skin collection had alkaloid **267A**, the only occurrence of an allopumiliotoxin among the four collections in this earlier study. Extreme variability was also seen in the following izidine (Iz) classes detected from the 2002 collections. All four collections had **195G** (5-propyl-6,8-dimethylindolizidine) or other 5,6,8-trisubstituted indolizidines and only two collections of the four had 5,8-disubstiuted indolizidines, however none of these shared common structures. Likewise, all collections had several izidines (Iz) or tricyclic structures but none of these alkaloids were shared.

The present study analyzing single skins of *M*. *rubriventris*, focuses on alkaloid profiles of a single species of toad from undisturbed contiguous and non-contiguous sites and whether any conclusions can be drawn as to the variety of arthropod prey consumed during their explosive breeding bouts that might allow a comparison in alkaloid composition with earlier collections taken during non-breeding periods.

We have deviated considerably from the data formats we used earlier in other journals in that we have included much more original data such as GC retention times, high resolution EIMS, CIMS, total-ion mass chromatograms and complete EIMS mass spectra (not just major peaks). We think this is important in appreciating the unexpected complexity and variation in the toad skin alkaloids of this species and the unique time and place of these collections. Almost half have never been seen before in any Old or New World frog or toad. The new alkaloids are not the result of improved analytical sensitivity or more concentrated samples as we have used basically the same protocols and instruments as in our earlier work on single-skins of mantellid frogs (Daly et al., 2008b).

The corrected GC retention times are necessary to confirm that a particular alkaloid is either new or a new diastereomer of a known alkaloid. In an effort to assist other investigators, this data (see Additional file 1 Tables S1-S10) will also update our earlier review that included approximately 800 poison frog alkaloids then known, with their corrected GC retention times (Daly et al. 2005). The total ion current (TIC) chromatograms (Additional file 3 Figure S1-S10) are critically illustrative here because of the alkaloid variability observed and also because of the wide range in alkaloid amounts. Because so many alkaloids are new, we cannot, as is usual, simply refer to characterization citations in previously published papers on poison frog or toad alkaloids. So we chose to enter the whole of our mass spectral data here in hopes of stimulating further work and as an explanation for the departure of these toads from expected analytical results, where only half a dozen or so new alkaloids would have been expected. With the current state of the art of electronic publishing, there is no good reason to depict the mass spectra in any other format but the graphical one, which is the best to compare with new data to be obtained in the future.

## Methods and materials

Two toads were collected at each of five sites: three sites in Salta province of NW Argentina and two sites in the contiguous province of Jujuy (see Figure 2). Both provinces border Bolivia (see inset, Figure 2). The two specimens from each site were captured within an area of roughly 4 m x 4 m at the dates indicated in the footnotes to Additional file 1 Tables S1-S11. Table S11 provides GPS coordinates and elevations of the sites along with the size and sex of the toads. The two photographs of Additional file 2 Figure S27 depict typical collection ponds.

After skinning in the field, the individual skins were collected in vials with MeOH (aprox. 1 ml) and brought to the lab, where they were cut into small pieces and triturated with MeOH (3 x 2 mL) in a small mortar. The methanolic extract for each individual skin was concentrated under nitrogen to a volume of 0.5 mL. To this, 0.5 mL of water was added, and the combined extract shaken with CHCl_3_ (3 x 1 mL). The chloroform layer was concentrated to 0.5 mL and diluted with hexane to 3 mL. The hexane-chloroform layer was extracted with 0.1N HCl (3 x 1 mL) and the hexane layer (containing neutral materials such as fatty acid esters and other lipids) was discarded. The acid layer was adjusted to pH > 8 with 1M ammonia, then extracted with chloroform (3 x 2 mL). The combined chloroform layers were dried (anhyd. Na_2_SO_4_), filtered and evaporated carefully to dryness under a nitrogen flow to yield the alkaloids. Methanol was added to yield a final volume of 0.5 mL. The weight of wet skins was not obtained in this study. Typically 1 uL of each extract was injected for GC-MS analysis. The spectrometric analysis used a Thermo Electron PolarisQ mass spectrometer interfaced with a Thermo Electron Focus gas-chromatograph fitted with a Phenomenex Zebron ZB-5 column (30 m, i.d. 0.25 mm) and programmed from 100°C (1 min) to 280°C at 10°C / min, with a hold time of 10 min. Chemical ionization (CI) used ammonia as the reagent gas. High-resolution mass spectrometry data were obtained with a Waters GCT mass spectrometer interfaced with an Agilent HP-6890 gas-chromatograph fitted with an HP-5 column (30 m, i.d. 0.25 mm) and programmed as before. GC-FTIR data were obtained for a few alkaloids with a Hewlett-Packard model 5890 gas-chromatograph fitted with an HP-5 column (30 m, i.d. 0.25 mm) and programmed as before, interfaced with a Hewlett-Packard model 5971 Mass Selective Detector and a model 5965B IRD with a narrow range (4000–750 cm^-1^) infrared detector.

## Results

### Assignment of alkaloid classes

The mass spectral identification protocols, discussed in much greater detail in Daly et al., (2005), are briefly as follows for the classes identified in this study: “Tri” are tricyclic alkaloids exhibiting many fragment ions, separated by 14 atomic mass units. Otherwise nearly all the alkaloids found here are bicyclic with a bridgehead nitrogen (‘izidines”) and display one major fragment ion (the 5,8-I and 5,6,8-I classes) or two significant ions (3,5-I, 3,5-P and 4,6-Q)(see footnote to Table 1 for an explanation of these abbreviations). The main pumiliotoxin (PTX) class, being also bicyclic izidines, exhibits a major ion at *m*/*z* 166 with an ion at *m*/*z* 70 of varying intensity. The alloPTX class has a major ion at *m*/*z* 182 and a weaker ion at *m*/*z* 70. Various “izidines” of undefined ring structures have one or two major fragments due to α-cleavage and often unsaturation in the ring system. The 2,6-disubstituted piperidines (monocyclic) show two α-cleavages.

**Table 1:** **Summary of alkaloid occurrence by extract**

Province		Salta		Salta		Jujuy		Salta		Jujuy		
Locale		El Nogalar		Huaico Chico		Los Paños		Tablada		El Cucho		
Alkaloid ^a^	Class ^b^	Toad 1	Toad 2	Toad 3	Toad 4	Toad 5	Toad 6	Toad 7	Toad 8	Toad 9	Toad 10	N ^c^
**169A** *	Unclass	x										1
**181F** *	Iz		x								x	2
**183C**	Unclass	-/x			-/x			-/x			x*/- ^d^	4
**193C**	Tri				x					x	x	3
**193G**	5,6,8-I					x*						1
**193N** *	Tri	x	x	x	x	x	x					7
**193O** *	Tri	-/x			-/x			-/x			x/-	4
**195G**	5,6,8-I	x/-	x/-	x/-	x/-		-/x*		x/-			6
**195L**	Unclass									x*	x*	2
**195N** *	Iz						x					1
**196**	Q	x	x			x	x			x	x	6
**199B** *	Unclass							x				1
**203B**	Tri	x	x		x			x	x			5
**203C** *	Iz		x									1
**203D** *	Unclass		x									1
**205G**	Iz	x*	x*	x*	x*							4
**205H**	Tri	x	x					x	x			4
**205K**	Tri							x				1
**205M** *	Tri							x				1
**207J**	Tri		x*		x*	x*	x*					4
**207N**	Unclass	-/x*	-/x*	-/x*						x*/x*	x*/-	6
**207U**	Tri							x	x			2
**207X** *	Unclass	x	x	x	x							4
**207Y** *	Tri							x	x			2
**207Z** *	Tri							x				1
**209B**	5,8-I										x	1
**209N**	Iz					x	x					2
**209T** *	Unclass				-/x			x/-				2
**211P**	Iz						x*					1
**211U** *	Unclass			x								1
**221Q**	5,6,8-I	x	x		x			x	x			5
**221R**	Iz	x/-	x/x*	x/-	x/-	x/-		x/-				7
**221S**	Tri	x*						x*	x*			3
**221Z** *	Tri			-/x	-/x					x/-	x/-	4
**221A2** *	Tri					x						1
**221B2** *	Tri							x				1
**223H**	3,5-P	x/-						x/x			x/-	4
**223T**	Unclass		-/x/−/−	-/x/x*/x	-/x/-/x		-/x/-/x			x*/x/x*/x	x*/x/x*/x	16
**223X**	5,6,8-I	-/x						x/-				2
**223AB**^e^	3,5-I	-/x			x/x							3
**223E2** *	Unclass				x							1
**225B**	Pip					x*	x*					2
**225O** *	Unclass									x	x	2
**225P** *	Tri	x										1
**225Q** *	Iz					x/-	x/x					3
**231B**	5,6,8-I			x	x							2
**231J**	Unclass							x	x			2
**235E**	5,6,8-I							x*				1
**235D2** *	Tri		x					x				2
**235E2** *	Unclass								x			1
**236**	Spiro				x		x					2
**237A**	PTX	x	x	x		x			x	x	x	7
**237O**	Tri						x*	x*	x*			3
**237W** *	Unclass					.	x					1
**239D2** *	5,6,8-I					x	x					2
**239E2** *	4,6-Q				x	x	x					3
**239F2** *	Amine									x	x	2
**241J** *	Amine								x			1
**247Q** *	Unclass	x										1
**247R** *	Tri	x	x	x	x		x	x	x			7
**247S** *	Tri						x					1
**249P**	Unclass	x*/x						x*/x	x*/x			6
**249C2** *	Unclass							x				1
**249D2** *	Unclass								x			1
**251D**	PTX	x	x	x	x	x	x	x	x	x	x	10
**251S**	5,6,8-I							x*/x/x	-/x/-			4
**251F2** *	Tri	x/x										2
**253O**	Unclass			-/x*						x*/-	x*/-	3
**253W** *	Unclass	x		x	x					x	x	5
**261D**	5,8-I							x*	x*			2
**261I** *	Unclass				x		x	x	x			4
**263U** *	Tri	x	x	x	x	x	x	x	x			8
**263V** *	Unclass							x	x			2
**263W** *	Tri							x	x			2
**265D2** *	Tri	x	x		x	x	x	x	x			7
**265E2** *	PTX	x	x	x	x							4
**267R**	5,6.8-I	x	x	x	x	x		x				6
**267Z** *	Unclass						x				x	2
**267A2** *	Unclass									x/x	x/x	4
**267B2** *	Unclass										x	1
**273E** *	De-5,8-I		x									1
**275D**	De-5,8-I		x*									1
**277B**	PTX				x	x		x	x	x		5
**277G**	PTX	x	x	x	x							4
**281P** *	Unclass	x										1
**281Q** *	5,8-I		x/x									2
**283G** *	Iz	x	x	x	x	x			x	x		7
**291G**	PTX	x/x	x/x	x/x	x/x	-/x	-/x			-/x		11
**305B**	PTX	x	x	x	x							4
**305I** *	PTX	-/x	x/x	x/x	x/x				-/x			8
**307A**	PTX	x	x	x	x				x			5
**307E**	PTX	x	x	x	x							4
**307G**	PTX	x/x*	x	x	x	x	x	x	x	x		10
**309A**	PTX	x							x			2
**309K** *	PTX	x	x	x	x							4
**321F** *	PTX								x			1
**323A**	PTX	x	x	x	x	x	x	x	x	x	x	10
**337D** *	PTX	x	x	x	x							4
**341A**	aPTX	x	x	x	x	x			x	x	x	8
**348A** *	Unclass									x	x	2
**364A** *	Unclass									x		1
**374A** *	Unclass				x			x	x			3
**379**^f^	PTX					x		x	x			3
**380A** *	Unclass							x/x				2
**104 alk**.		46^g^	42^g^	34^g^	45^g^	24^g^	26^g^	43^g^	35^g^	27^g^	28^g^	

^a^ An asterisk after an alkaloid code name indicates an unreported alkaloid, or after an "x" indicates an unreported diastereomer on GC R_t_. See Additional file 1 for HRMS data on 19 fragments of the 56 new alkaloids. See Additional file 1 Tables S1-S10 for observed and corrected GC retention times and relevant literature R_t_ data.

^b^ Abbreviations used: 3,5-P, 3,5-disubstituted pyrrolizidine; 3,5-I, 3,5-disubstituted indolizidine; 5,8-I, 5,8-disubstituted indolizidine; 5,6,8-I, 5,6,8-trisubstituted indolizidine; De-5,8-I, dehydro-5,8-disubstituted indolizidine; Q, quinolizidine; Iz, izidine; 4,6-Q, 4,6-disubstituted quinolizidine; PTX, pumiliotoxin; aPTX, allopumiliotoxin; Spiro, spiropyrrolizidine; Tri, tricyclic; Unclass, unclassified as to structural type.

^c^ Number of occurrences of the coded alkaloid and diastereomers throughout the ten skins.

^d^ Where one or more diastereomers are indicated for a formula, these are listed in the order of increasing R_t_ on GC.

^e^ The indolizidine of shorter R_t_ is the 5*Z*,9*Z* diastereomer; the indolizidine of the longer R_t_ is the 5*E*,9*Z* diastereomer.

^f^ Alkaloid **323A** is occasionally observed at *m*/*z* 379 as the dimethylsiloxane, a GC artifact.

^g^ Total number of alkaloids including diastereomers detected in each extract.

### Analyses of the toad skins

The ten skins were found to contain 127 different alkaloids in total, 56 of which have not been detected previously (Table 1). The EIMS of alkaloids have an odd mass for the molecular ion (if they contain an odd number of nitrogen atoms) and generally have even-mass fragments for the major peaks, when the fragments result from a cleavage of a radical from the molecular ion and neutral molecule cleavages after that (see Additional file 4: Figures S11-S26, for the mass spectra of all 127 alkaloids). The largest structural classes of alkaloids observed in this study are tricyclic structures (24) and pumiliotoxins (18) with a significant number (31) being unclassified as to structural type. The present collections show only one allopumiliotoxin, alkaloid **341A** (found in seven skins), one piperidine and two alkaloids classified as amines. Most of the remaining alkaloids are of the izidine (Iz) classes, either previously detected and having assigned structures (14), newly detected (codes with asterisks), but having assigned structures (5) or having MS fragmentations indicating only mass spectral patterns typical of the izidine category (9). Of these latter izidines to which no tentative structure has been assigned, four were previously known and five are new (Table 1).

Of the 56 new alkaloids, 24 are unclassified, 16 are considered to have tricylic structures, nine have various izidine (e.g. indolizidines) structures and five of the remaining seven are of the pumiliotoxin class. The alkaloids found in each toad skin are listed in Additional file 1: Tables S1-S10. We have included a truncated version of Additional file 1: Table S1 (Table 2) below as an example of how the data is presented in these Tables S1.

**Table 2:** **Data for toad # 1 (truncated, see Sup. Info)**

R_t_(obs.)	R_t_(corr.)	Alkaloid	Class	Comments
4.63	3.66	**169A** *	Unclass	CI 170; C_11_H_23_N
5.96	5.02	**183C**	Unclass	CI 184; C_12_H_25_N; M-CH_3_ = C_11_H_22_N
6.73	5.80	**193N** *	Tri	CI 194; C_13_H_23_N
6.92	5.99	**195G**	5,6,8-I	CI 196; C_13_H_25_N
7.59	6.68*	**207N**	Unclass	CI 208; C_14_H_25_N
				IR ν_C=CH-_ 3015 cm^-1^
8.14	7.24	**207X** *	Unclass	C_14_H_25_N
8.29	7.39*	**205G**	Iz	C_14_H_23_N
8.74	7.85	**205H**	Tri	CI 206; C_14_H_23_N
8.84	7.95	**193O** *	Tri	CI 194; C_13_H_23_N analog of **193C**
16.64	15.91	**305B**	PTX	
16.71	15.99	**305I** *	PTX	C_19_H_31_NO_2_
16.84	16.12	**307E**	PTX	
17.36	16.65	**341A**	aPTX	
17.45	16.74	**337D** *	PTX	C_20_H_35_NO_2_
18.07	17.37	**323A**	PTX	

The alkaloids present in each toad skin are tabulated in order of increasing GC retention time. The observed retention times of the detected alkaloids are corrected to compare with those in the most recent summary of the anuran skin alkaloids (Daly et al. 2005). These proved critical in the present work in identifying new diastereomers of known alkaloids. In column 3 of each table is our identification of the toad alkaloid at that R_t_ with a previously detected anuran skin alkaloid. Alkaloids not seen previously are given a new code and followed by an asterisk (e.g. 169A^*^). In column 4, we report the structural class, if known, of that alkaloid. Additional comments are included in column 5. Such data indicates a formula (most formulae were determined by high resolution mass spectrometry (HRMS)) and the chemical ionization (CI) molecular ion (M+H)^+^ determined with ammonia reagent gas. The formulae permit the calculation of double bond equivalents, i.e. rings and/or unsaturations. A few formulae are estimated and are in quotes. We have provided electron-impact HRMS data on approximately 70 alkaloids, most previously unreported. In many instances, major fragments were also mass-measured (see Additional file 1: Table S1-S10 for this data). A few vapor-phase infrared spectral data are included in Additional file 1: Tables S1-S10, with indicated frequencies of selected bands.

Related to each table is an annotated total ion current chromatogram (TIC) (Additional file 3 Figures S1-S10) to allow the reader to visually estimate relative amounts of alkaloids. We have supplied the chromatograms to allow this way of quantitation, rather than use our more common tabular methods of classifying major, minor and trace components that are approximate only. The observed gas chromatographic retention times (R_t_ (obs.)) in Additional file 1 Tables S1-S10 are included to allow the identification of GC peaks in the ten total-ion-current chromatograms. Only the most significant peaks are annotated in those chromatograms. The chromatogram TIC intensities all fell in the range of 1.7 x 10^7^ to 9.2 x 10^7^ (see Additional file 1 Table S13, for this data).

Table 1 is a compilation of the data of Additional file 1 Tables S1-S10 where the toad extracts are organized by collection sites and an “x” indicates an alkaloid’s presence and any accompanying diastereomer. A new alkaloid is indicated by an asterisk after the code name in all tables. One or more new diastereomer(s) is(are) indicated by the symbol x*. A footnote to Table 1 indicates the alkaloid class abbreviations used in that table and in Additional file 1 Tables S1-S10.

**Figure 3:**
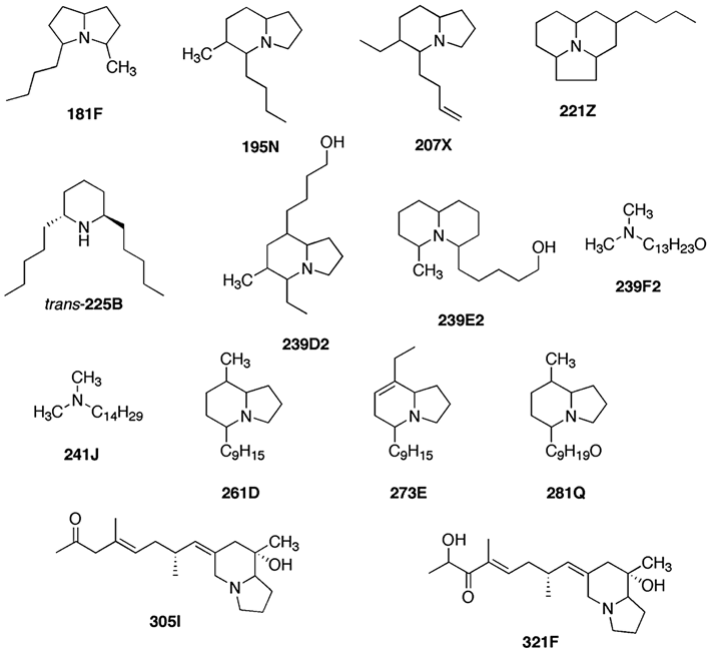
**Tentative structures of some of the new alkaloids found in skins of*M***. ***rubriventris.***

In Figure 3, we indicate tentative structures of 14 of the new alkaloids as determined by HRMS and analogy with known frog or toad skin alkaloid mass spectral fragmentation patterns. In the present study, slightly more than half of the total alkaloids we observed, fall into a molecular weight range below 240 a.m.u., which is lower than typically seen in dendrobatid or mantellid frog skin alkaloids.

In this paper, we continue the overall scheme of indicating alkaloids with a nominal molecular weight in bold and a bold letter that distinguishes alkaloids of the same nominal mass. We have abandoned however the continuation of alphabet codes for letters past “Z” with double letters such as AA, BB etc., as both being unwieldy and having limitations of 26 letter pairs. We have instead adopted A2, B2, etc., to replace double identical letters and if the occasion arises in the future we would then use A3, B3, etc. Consequently, an alkaloid, **249AA**, that already appears in the literature is now renamed as **249A2**. This nomenclature is used in the present work and will be used by us in any future work.

The following seven alkaloid code names, new since an earlier review (Daly, et al. 2005), were used in our earlier *M* paper (Daly et al. . *rubriventris*^2007^): **193M**, **247L**, **247M**, **251E2**, **255F**, **265B2** and **267Y** (**251EE** and **265BB** are now renamed as **251E2** and **265B2**). Five were unclassified and two were indolizidines. Curiously, none are seen in the current study.

By using a similar GC-column as ours (DB-5, HP-5, OV-5 and similar ones) and the same GC-program, we hope that other investigators will be able to replicate our results with their own samples of frog or toad skin alkaloids. They will be able to see and identify previously detected alkaloids, create their own corrected R_t_ and characterize new alkaloids. Although the mass spectra are going to be somewhat different than ours if using different instruments with different detectors, the general pattern will be identifiable by comparison with these 127 mass spectra we present in the Additional file 4 Figure S11-S26. Hopefully, they will also name the new alkaloids (identified or not, but at least characterized by MS and R_t_) following our code, adding to the list of more than 900 alkaloids detected in frog skin so far.

The complete electron-impact mass spectra of all alkaloids reported in this paper, 104 + 23 diastereomers, are shown in Additional file 4 Figures S11-S26, eight spectra per page in order of increasing MW, except for **195N** added at the end.

The present work extends the earlier study of *M*. *rubriventris* on extracts of combined skins and confirms now with single skin extracts, the extreme variability (both in time and location) of alkaloid contents in the skins of these toads. This point is expanded upon in the Discussion Section.

## Discussion

*Variation in Alkaloid Structures Found in Skins of M*. *rubriventris Toads*. One of our most unusual findings is the scarcity of alkaloids considered to derive from straight-chain precursors, here represented by a few examples only: a 3,5-disubstituted pyrrolizidine, 3,5-disubstituted indolizidines, a 4,6-disubstituted quinolizidine and a 2,6-disubstituted piperidine. The alkaloids having straight chains were significant classes in *M*. *stelzneri*, *M*. *cupreuscapularis* and *M*. *klappenbachi* but were also commented upon as being deficient in four earlier collections of *M* (Daly et al. . *rubriventris*^2007^) where 2,5-disubstituted decahydroquinolines were also missing as true of the present survey. These are all thought to originate from ants (Jones et al. 1999; Spande et al. 1999; Daly et al. 2000). To complicate this simple picture, examples of izidines of straight-chain structures such as **223AB**, a 3,5-disubstituted indolizidine and a 4,6-disubstituted quinolizidine, respectively, have been reported from oribatid mites (Saporito et al. and **237I**^2007^). Furthermore, pumiliotoxins, necessitating branched structures, were found, unexpectedly, in two tiny species of the formicine ant genera *Brachymyrmex* and *Nylanderia* () (Saporito et al. formerly *Paratrechina*^2004^).

In our present study, the great majority of alkaloids, e.g. tricylic structures, are either inferred or known to be of branched-chain structures and likely originate from mites (Saporito et al. 2007). Seven of the branched-chain alkaloids are of the known 5,6,8-trisubstituted indolizidine class. Table 3, derived from Table 1 lists the totals of three such branched-chain alkaloid classes: pumiliotoxins, tricyclic alkaloids and the sum of 5,8-disubstituted indolizidines and 5,6,8-trisubstituted indolizidines. No consistent pattern emerges of putative mite preferences although toads from the Salta sites #1-4 concentrate pumiliotoxins and from the Salta sites #1, 7, 8 are rich in tricyclic structures. The indolizidines appear at random among the ten skins.

**Table 3:** **Some alkaloid classes with branched chains found in*M***. ***rubriventris***^**a**^

Collection	1	2	3	4	5	6	7	8	9	10
PTX	20	16	16	16	8	4	5	11	7	4
Tri	11	8	4	9	5	7	16	10	2	2
5,8-I + 5,6,8-I	4	7	3	4	3	2	8	4	0	1

^a^Includes diastereomers.

Another interesting finding is that 23 of the total 127 alkaloids (see “x^*^ ", Table 1) are new diastereomers of previously reported alkaloids. We have never before found this many diastereomers of alkaloids in toad/frog skin extracts. Alkaloid **223T** occurs, for example, as four diastereomers in both skins from the El Cucho site. Two of these diastereomers have not been seen previously. The new diastereomers of known alkaloids are 9 from izidine structures, 8 from unclassified structures, 4 from the tricyclic class, and one each from pumiliotoxin and piperidine classes. A total of 6 diastereomers are seen with the combined 5,6,8-I and 5,8-I classes but there seems otherwise little association of diastereoisomerism with any structural class, nor is there any association of diastereoisomerism with diet species of toad / frog or time of year.

An unexpected finding is the occurrence of 1-acetamidoquinolizidine (code “Q”), named epiquinamide, in six of the ten skins. This alkaloid had previously been detected only in dendrobatid poison frogs of the species *Epipedobates tricolor* () of Ecuador (Fitch et al. now *Ameerega anthonyi*^2003^) but in very minor amounts. It has not been seen elsewhere until the present study.

The likely artifact, 4-aminobenzoic acid ethyl ester, probably originating as a sunscreen component, occurred in four extracts (# 1, 2, 7, 8), accompanied by the *N*-acetyl derivative, an anesthetic component, in extracts # 7 and 8. A likely contaminant, a toluamide insect repellent, was detected in extract # 3.

A curious difference with results reported with other *Melanophryniscus* studies is the accompaniment of PTX **251D** in every one of our extracts by many other pumiliotoxins, some in substantial or even major amounts, e.g. **237A**, **307A**, **307G**, **309A**, **323A** and **341A** (see chromatograms of Additional file 3 Figures S1-S10). These results stand in contrast to those of Mebs et al. (2005, 2007) who have found pumiliotoxin **251D** as the principal skin alkaloid in the three *Melanophryniscus* extracts they have examined from Uruguayan species and have reported traces only of six other accompanying pumiliotoxins, one a homopumiliotoxin. One of the pumiliotoxins was **267C** which we have not observed, although it was observed by us in a much earlier study of the Brazilian toad *M* (Daly, et al. .1984*moreirae*). Traces of uncharacterized indolizidines were also reported in *M* (Mebs et al. . *montevidensis*^2005^). We have not detected in the present study any of the homopumiliotoxin alkaloids such as **249F** earlier found with accompanying diastereomers in *M*. *klappenbachi* or *M*. *cupreuscapularis* (Daly et al. 2008a) although we did observe four of the pumiliotoxins (**251D**, **277B**, **307G** and **323A**) in one or the other collection. In the earlier survey of four collections of combined skins of *M*. *rubriventris* (Daly et al., we found many PTXs. However, **251D**^2007^) was found in only two of the four collections; one, a nine-skin extract from Canto del Monte, Salta and the other, a two-skin extract from Tiraxi, Jujuy (see Figure 2in Daly et al. 2007). Incidentally, it should be pointed out that a recent study on two tiny species ( frogs from Cuba, (Rodriguez et al. SVL ≈ 10 mm) of *Eleutherodactylus*^2010^) indicated they contain pumiliotoxins. Thus a fifth family of poison frogs can now be added to the anuran families Bufonidae, Dendrobatidae, Mantellidae and Myobatrachidae. Stomach contents of these eleutherodactylids indicated they had consumed mainly mites.

A striking observation in the current study is the large number (30) of alkaloids that occur in just one of the skins but none of the others. Table 4 indicates the frequency of alkaloids that occur once, twice, three times, etc. among the ten skins examined. As the third column indicates, 24 (80%) of these “singletons” have not been seen before in *any* of the four anuran families we have examined for alkaloids. Even the 24 alkaloids that occur only twice among the ten sites contain 14 new structures. That the new structures are found mainly restricted to one or two toads must indicate a dietary arthropod of restricted availability or preference.

**Table 4:** **Occurrence of any individual alkaloid among the ten*M***. ***rubriventris*skins examined**^**a**^

Frequency of occurrence	Total such alkaloids	New alkaloids among them
Once	30	24
Twice	24	14
Three times	9	3
Four times	17	8
Five times	5	1
Six times	5	0
Seven times	6	4
Eight times	3	2
Nine times	0	0
Ten times	3	0

^a^ Includes diastereomers counted together with the major alkaloid.

The large number of single occurrences of alkaloids we report here has not been seen previously among single frog skins of *Dendrobates* (*Oophaga* (Saporito et al. ) *pumilio*^2006^), *Mantella* species (Daly et al. 2008b; Clark et al. 2006toads (Mebs et al. ), or *Melanophryniscus montevidensis*^2005^of the family Myobatrachidae (Smith et al.). Since most of the poison anurans, except frogs of the genus *Pseudophryne*^2002^), do not synthesize any of their skin alkaloids, but rather sequester them from dietary arthropods (Saporito et al. 2009), this implies an extremely varied choice of prey items for *M*. *rubriventris*, the greatest we have yet seen. The diet of all analyzed populations of *M* contained several main prey categories with numbers ranging from 17 to 32 as described by Bonansea and Vaira (. *rubriventris*^2007^) and Quiroga et al. (2011). Dendrobatid frogs are capable in some species of metabolizing **251D**, by an enantioselective 7β-hydroxylation (Daly et al. to the more toxic allopumiliotoxin **267A**^2003^) and it is likely that some PTX-containing *Melanophryniscus* toads can also accomplish this, although this remains to be demonstrated. The Australian frogs of the genus *Pseudophryne* that have been studied, synthesize their pseudophrynamine alkaloids (-methyltryptamines). In the wild, they also accumulate pumiliotoxins from diet and the pseudophrynamines decline. When reared in captivity, however, PTX alkaloids are not found in skins (Smith et al. isoprenylated cyclized *N*^2002^).

Often the number of diastereomers detected of a given alkaloid in the current study is observed as one, two or even four, whereas previous work on dendrobatids or mantellids would yield mainly one or two diastereomers in any single extract (e.g. Garraffo et al. 1993b). The use of the term diastereomer refers to an alkaloid differing only on the spatial arrangement of the side-chains or functional groups but sharing the same skeleton and position of the groups as in another alkaloid. Diastereomers differ in most physical properties, including NMR and IR spectra but show very similar mass spectral fragmentation. Therefore, with GC-MS we observe for them different retention times but similar EIMS and the same molecular formula. In this study, with so many new alkaloids, related in structure to known alkaloids and diastereomers of known alkaloids, we have relied heavily on GC retention times in addition to EIMS. If a retention time differed by more than ± 0.15 min from the R_t_ previously tabulated (Daly et al. 2005) and the EIMS was very similar, we have considered it to be a new diastereomer, although in some cases it could be simply a positional isomer. In several instances, a new diastereomer was detected but none of the previously known diastereomers were found.

Table 1 indicates the variability of alkaloid numbers and profiles (including diastereomers) among the ten skins, ranging from a low of 24 alkaloids in skin # 5 to 46 in skin # 1. As noted from Tables 1 and 4, there is a curious frequency increase in four alkaloids being shared; seven of the 17 instances involve 4 occurrences of the same alkaloid shared only between sites # 1–4. In addition there are 13 cases where an alkaloid is shared with each of sites # 1–4 and one or more of the other sites (see Table 1). The four single skins of toads from the Jujuy province, whether from Los Paños (# 5–6) or El Cucho (# 9–10), have fewer alkaloids per skin than those from Salta (no statistical analysis done), ranging from 24–28 alkaloids per skin, although the alkaloid compositions vary greatly (see Additional file 3 Figures S5, S6 and S9, S10). The pair of skins # 7–8 collected roughly one year later at Tablada, also in Salta, differed considerably with one another.

### **Geographical variation of alkaloid profiles for the toad*M. rubriventris***

It is unexpected that two toad skin specimens from the same collection site (e.g. # 5, 6) would show such variation in alkaloid profiles as we have observed, although it must be stressed that the toad captures in this study were made during one of the usual “explosive” three-day breeding episodes in or near small ponds (Vaira 2005), and toads were not sampled in the nearby forested areas where they could also forage. Since the alkaloids are acquired and accumulated through the diet of small arthropods and persist for a long time within the toad skin, significant heterogeneity of skin alkaloids in the sampled toads could be expected, although we are still surprised at the extent seen here. Frequent migrations to and from such breeding ponds have recently been demonstrated for both males and females of the Brazilian toad *M*. *cambaraensis* (Santos et al. 2010b). This movement of the toads from sheltered areas to congregations around ponds for reproduction and back to the forest, could have important consequences for the foraging strategies and evolution of these species.

Table 5, that is derived from inspection of Table 1 and has not been statistically analyzed, illustrates that a greater number of alkaloids are shared in skins of the two specimens collected at the same site (or even in some cases at contiguous sites such as # 1 and 4 with 29 alkaloids in common) than between skins of toads collected at two more widely separated sites (Figure 2). This would argue that, even though feeding by the toads during reproduction is occurring near the ponds, the terrestrial foraging range of captured specimens is not great at this time and that a similar diet of arthropod prey is being consumed over a longer period than just during the breeding bouts (Quiroga et al. 2011). Complicating this observation somewhat are the data from sites # 5 and 6 that appear to deviate from the expected value and share nearly as many alkaloids with widely separated sites as the same site, e.g. # 4 and 5; # 4 and 6 and # 5 and 6 *all* share 14 alkaloids. The contiguous sites # 7 and 8 and # 9 and 10 share 24 and 20 alkaloids respectively.

**Table 5:** **Alkaloids shared between skins of*M***. ***rubriventris***

Site	1	2	3	4	5	6	7	8	9	10
1	---	28	26	29	13	9	17	19	10	7
2	---	---	28	29	14	11	12	15	10	7
3	---	---	---	29	11	9	7	11	12	8
4	---	---	---	---	14	14	15	15	11	7
5	---	---	---	---	---	14	8	10	9	5
6	---	---	---	---	---	---	8	8	7	6
7	---	---	---	---	---	---	---	24	4	3
8	---	---	---	---	---	---	---	---	7	4
9	---	---	---	---	---	---	---	---	---	20

Table 5 indicates the number of alkaloids, including diastereomers, shared between one skin and another, e.g. skin # 1 and skin # 2 have 28 alkaloids that are shared.

### T**emporal variation in skin alkaloids of*M. rubriventris*toads**

Significant temporal differences were noted with *M* collections from the same site sampled ten years apart in Córdoba province (Daly et al. . *stelzneri*^2007^). Relevant to this point, are our present studies of *M*. *rubriventris*, that indicate the roughly contiguous sites of El Nogalar (# 1–2) or Huaico Chico (# 3–4) and Canto del Monte in Salta province sampled six years apart show a much greater alkaloid overlap, than is evident between frogs collected in Tiraxi, Jujuy province in 2002 and at El Cucho, Jujuy (# 9–10) in 2008, where strangely only approximately 20% of the alkaloids are shared (Figure 2; Additional file 1: Table S14). The pair at the slightly more distant site of Los Paños (# 5–6) however shared more alkaloids, not less with the Tiraxi site. Alkaloids shared between Abra Colorada and either the El Cucho or Los Paños sites of Jujuy province are fewer for the former than for the latter (Additional file 1: Table S14). Although the numbers are limited in these *M*. *stelzneri* and *M*. *rubriventris* toad collections, the skin analyses we found, deviate from the consistency we have come to expect with dendrobatid or mantellid frogs collected from the same or contiguous sites. There is, however, no statistical analysis, since the experiments were not suitable.

Skins from either the Cedral de Baritú and/or the Canto del Monte sites in Salta province had respectively 5 and 8 of the pumiliotoxins seen in one or more of the present collections except that the previously reported PTX **289C** alkaloid is now absent. Nine of the other alkaloids (mainly izidines and tricyclics) were also seen in the present collections; however 4 of the other 19 alkaloids from the two 2002 collections were not found in the present three collection sites from Salta (see Additional file 1: Table S13). The situation with the Jujuy collections is more complex in that few of the alkaloids from the 2002 Abra Colorada site were found in the present Jujuy collections (# 5–6 and # 9–10, see Additional file 1: Table S14). However *all* of the alkaloids from the 2002 Tiraxi site were found in one or more of the current Jujuy collections. Also in the 2002 collections, significant alkaloid variability was noted with five Jujuy alkaloids not being shared with the Salta toads while nine alkaloids from Salta toads were not shared with the Jujuy toads (see Additional file 1: Tables S13, S14).

The current collections from Los Paños (# 5–6) and Tablada (# 7–8) were made approximately one year apart and are also from the most non-contiguous sites of our studies (separated by ca. 150 km), thus a geographical factor is also involved here. Relevant to this separation in space and time is the fact that they share only 16 of the total 32 alkaloids (averaged from skins # 5–8), mainly tricyclic structures and pumiliotoxins (Table 1), supporting the view that toads from non-contiguous sites collected a year apart will share even fewer alkaloids as the local prey items will have varied. Only five alkaloids were found in common in toad skins at every one of the four sites, # 5–8.

It should be emphasized that comparisons with either single skin or combined skins of dendrobatid or mantellid frogs collected from nearby sites at approximately the same time have always given alkaloid compositions that are comparable in amounts and profiles. Although it must be stressed that the comparisons within Additional file 1: Tables S13 and S14, are between single (A) and pooled (B) skins, there are many fewer alkaloids in common than we would have expected from sites that are fairly contiguous.

We have no explanation at this time as to why the bufonid *M* should deviate so significantly from the non-bufonid pattern and can only presume that the presently observed differences between the 2002 (Daly et al. *rubriventris*^2007^) and the current 2007/2008 collections of *M*. *rubriventris* from Salta and Jujuy provinces reflect both short and long term variation in dietary arthropod availability. For example, one or more unknown arthropods in the *Melanophryniscus* toads’ locales may have an erratic appearance due to seasonal conditions such as rainfall, temperatures, the timing of hatches, migration patterns of both toad and prey, etc.

### **Diet and skin alkaloids in*M. rubriventris***

A recent study on the level of individual variation in diet composition of different *M*. *rubriventris* populations showed that the most important prey categories in the diets of the populations studied consisted of the same ground-dwelling arthropods, but evidence was found also for individuals consuming different arrays of alternate prey types added at low frequencies in all populations (Quiroga et al. 2011).

Stomach content or gastrointestinal tract analysis, although corroborative, are not conclusively reliable of exact dietary frog or toad prey preferences. Studies on three *Melanophryniscus* species indicated myrmicine ants to predominate in the cases of *M*. *cupreuscapularis* and *M*. *klappenbachi*, with acari (unidentified mites) being absent in the former and minor in the latter (Daly et al. 2008a). Three other studies examined the stomach contents of four populations of *M* from NW Argentina with a substantial number (13–60) of specimens examined and particular attention paid to arthropods (Bonansea and Vaira . *rubriventris*^2007^; Daly et al. 2007; Quiroga et al. 2011). Samples observed with both low and high proportions of mites, yielded PTXs as predominant alkaloids in the skin, so no direct correlation with branched-chain alkaloids and mites can be made in these cases. Ants were equivalent in numbers to mites in Abra Colorada and Canto del Monte, greatly preponderant in Cedral de Baritú, but minor in Tiraxi (Figure 2, Daly et al. 2007). Incident to the current 10-toad collection is examination of stomach contents of *M*. *rubriventris* by a non-destructive technique using flushing. This finds the prey types to be mostly ants, mites and collembolans (Quiroga et al. 2011). Stomach flushing furthermore has indicated the toad’s stomachs are empty during the night, when sporadic breeding still occurs (M. F. Quiroga and M. Vaira unpub. data). This statement is supported by the following two experiments:

, ranging in snout-to-vent length from 25 to 50 mm. The results indicate that prey items pass out of a frog stomach in 10–14 h after being ingested (Woolbright & Stewart, 1) A laboratory test of stomach passage time was performed in a similarly sized frog, *Eleutherodactylus coqui*^1987^). So, given that *Melanophryniscus rubriventris* is active early in the morning (around 7 am) we can expect that in these toads, foraging during the daylight, the stomach would be empty by midnight (see next).

2) During 2007/2008 some of us (MFQ and MV) collected 71 *M* toads and analyzed the stomach contents by the stomach-flushing method (Leger and Sullivan, . *rubriventris*^1979^; Solế et al. 2005) with approximately equal number of toads taken at 3 pm, 7 pm and 1 am the next day, defined as afternoon, evening and night, respectively. By flushing, the stomach eliminates all the contents (full stomach) in one bolus or almost nothing when the stomachs are empty. The proportions of full stomachs over all the frogs taken in each period, afternoon, evening and night, were 66%, 48% and 9%, respectively. Therefore, it seems clear to state that toads feed mainly during the morning until afternoon hours, although we found a few specimens still active at night (around 1 to 2 am).

The major problem with inferences from stomach contents is that such results represent the last meal or two of the toad, while the observed skin alkaloids are accumulated over a much longer time, likely an adult’s lifetime. Also the softer bodied prey items will be digested more rapidly than the hard-bodied mites and ants. Nevertheless, stomach content analyses do provide presumptive evidence of the dietary preferences of the poison frogs (Clark et al. 2005; Woodhead et al. 2007; Saporito et al. 2012) and toads so far examined. Of more limited application has been the analysis of toad feces using electron microscopy techniques to identify undigested body parts of prey (Mebs et al. 2005).

### A sex-link with alkaloids in *M. rubriventris* toads?

Of the ten skins examined, nine were from males and only one from a female (#8, Tablada site). While earlier studies of the dendrobatid frog *Dendrobates* (*Oophaga*) *pumilio* (Saporito et al. 2008) indicated some significant differences with sex in alkaloid profiles in both amounts and components, the present study, reveals no obvious differences in variety of alkaloids when all the toad males are considered. The *M* collection of 2001 reported in 2007 (Daly et al. . *stelzneri*^2007^) examined two combined male and two combined female skins and the results neither in amounts (male, 9 major/minor alkaloids; female 8 major/minor alkaloids) nor variety (male 23 alkaloids total; female, 22 alkaloids total) resembled the results found in studies with mantellid (Daly et al. 2008b) or dendrobatid frogs (Saporito et al. 2010), where the slightly larger and presumably wider ranging females had skin alkaloids in both larger amounts and greater variety. The lack of a sex link seen with currently investigated toad alkaloid profiles, although from very limited samples (in fact, only one female toad), suggests that the male and female *Melanophriniscus* toads may not separate significantly during foraging.

## Conclusion

The present ten collections were made during the lengthy, hot, spring-summer breeding season of these toads (November to February) where toads were captured as pairs in small (ca. 4 m x 4 m) areas with ponds (Additional file 2: Figure S27). The Salta and Jujuy regions where these toads are found are of a typical subtropical humid montane/woodland forest that is mature and well-structured. Moisture and occasional fog arise from ascending air currents and promote a lush epiphytic growth of bromeliads, ferns, mosses and liverworts. Temporary ponds and streams where breeding occurs arise from runoff from higher elevations during the rainy season (October to April) when average yearly rainfall is over 2 m, but are short-lived due to the steep slopes and porous soils (Vaira, 2005). Several breeding periods occur during the rainy season.

The fact that specimens collected from the same site have more alkaloids shared than toads from different sites, suggests that during the approximately three-day breeding period, arthropods are being consumed at or near the site, but overlaying this fresh input from a limited area will be a larger background of previous alkaloid sequestration that likely represents foraging from a more extensive area in the forest as supported by some preliminary observations of ours. The varying profiles of contents of stomach analyses also implies this. At the moment, it is not clear how extensive is the territorial range of the toads that just happen to be collected at the same time in the same small pond. Proximity of the collected toads in the present study, in any case, is misleading since foraging during intermittent breeding, both close to and more remote from such ponds, has been observed by us. Work is underway to determine more exactly the territorial range of toads on land or adjacent swamp areas before and during their typical three-day breeding bouts in the ponds.

Of the 48 previously reported alkaloids (i.e. those having codes followed by no asterisk in Table 1, Additional file 1 Tables S1-S10) observed in the present survey of the skin alkaloids from *M*. *rubriventris* toads, most were seen before in more than one of the other anuran families. The Venn diagram of Additional file 5 Figure S28 indicates that 22 of the 48 alkaloids were seen earlier in skin extracts of dendrobatid and/or mantellid poison frogs, while only 9 alkaloids were previously reported from bufonids alone. Thus, it appears that we are beginning to find more similarity in alkaloid structures between these poison toads of South America and the poison frog families of both hemispheres. This implicates common dietary prey and the common evolution of an alkaloid uptake system between toad and frog. The six previously reported bufonid alkaloids shared with dendrobatids include one 5,8-disubstituted indolizidine, one izidine and one tricyclic, all likely alkaloids with skeletal branch points and of probable mite origin. Interestingly, there are currently no known mantellid alkaloids shared only with bufonids. There are, however, 11 alkaloids shared among bufonids, mantellids and dendrobatids. Only two (**223H**, **223AB**) are known to have structures with a linear carbon-chain; the rest have branch points.

Very recent work (Grant et al. 2012) on eleven *M*. *simplex* skins from three sites in Brazil indicates the occurrence of many new alkaloids or diastereomers of known dendrobatid alkaloids as we have observed with the Argentinian *M*. *rubriventris*. Fourteen of the total of 47 alkaloids or new diastereomers they report are shared with our collections but the overall pattern they observe has many more alkaloids (eleven) likely sequestered from ants. Pumiliotoxins and various izidines dominate their collections but they also include nine alkaloids of unclassified structures, two that we also report.

 species and concluded that the observed strongly diurnal reproduction preference likely preceded the development of sequestration of toxic alkaloids in these and other bufonids and in poison frogs in general (Santos and Grant Santos and Grant have recently studied in detail the reproduction of a Brazilian *Melanophryniscus*^2010^). What is not clear at the moment is at what stage in evolution, did coloration begin to play a role to advertise toxicity.

## Electronic supplementary material

Additional file 1 Table S1-S10.: The R_t_ (obs) of the known alkaloids in the present study was corrected to compare with the corresponding retention times of our most recent summary (Daly et al.2005). A simple plot with observed vs. corrected retention times of the known alkaloids, selected from several of the tables below, generates a straight line; the corrected retention times for the unknowns are obtained using the observed values and interpolating within this line. Both values, R_t_ (obs.) and R_t_ (corr.), were fairly reproducible among the tables S1-S10 for any repeated alkaloid. **Table S11**: Collection information on skins of *Melanophyriscus rubriventris*. **Table S12**: GC-eims ion current intensities. **Table S13**: Effect of proximity between 2002 and 2008 collections-Salta province. **Table S14**: Effect of proximity between 2002 and 2008 collections-Jujuy province. (DOC 594 KB)

Additional file 2 Figure S27.: Typical ponds where collections were made: a) Cedral de Baritú, Salta; b) Los Paños, Jujuy. The blue parallelogram indicates the average collection area. (ZIP 9 MB)

Additional file 4 Figures S11-S26.: Mass spectra of all the alkaloids (127) found in this study, 8 spectra per page (total of 16 pages) in increasing order of MW, with the exception of alkaloid **195N** added at the end. (ZIP 2 MB)

Additional fle 3 Figures S1-S10.: Total mass spectral ion current chromatograms for the alkaloid extracts of toad skin samples #1-10. (ZIP 12984 kb) (ZIP 9566 kb) (ZIP 13 MB)

Additional file 5 Figure S28.: Venn diagram showing occurrence and overlaps of previously known anuran skin alkaloids (N = 48) detected in the Argentinian toad *Melanophryniscus rubriventris* of this study with those previously seen in bufonid toads, dendrobatid or mantellid frogs. See Supplemental Information of Daly et al. 2005 for alkaloids and sources. The asterisk in the dendrobatid/mantellid alkaloid overlap indicates two occurrences also in myobatrachid frogs of Australia. (ZIP 177 KB)
