# Supplementary material for: Alkaloids from single skins of the Argentinian toad Melanophryniscus rubriventris (ANURA, BUFONIDAE): An unexpected variability in alkaloid profiles and a profusion of new structures
Source: Springerplus. 2012 Nov 23;1(1):51. doi: 10.1186/2193-1801-1-51 (PMC3625416; doi:10.1186/2193-1801-1-51)
Supplement: Supplementary file 1 — Additional file 1 Table S1-S10.: The Rt (obs) of the known alkaloids in the present study was corrected to compare with the corresponding retention times of our most recent summary (Daly et al.2005). A simple plot with observed vs. corrected retention times of the known alkaloids, selected from several of the tables below, generates a straight line; the corrected retention times for the unknowns are obtained using the observed values and interpolating within this line. Both values, Rt (obs.) and Rt (corr.), were fairly reproducible among the tables S1-S10 for any repeated alkaloid. Table S11: Collection information on skins of Melanophyriscus rubriventris. Table S12: GC-eims ion current intensities. Table S13: Effect of proximity between 2002 and 2008 collections-Salta province. Table S14: Effect of proximity between 2002 and 2008 collections-Jujuy province. (DOC 594 KB) [file 40064_2012_198_MOESM1_ESM.doc]

Supplemental Information:

[Tables S1-S10]

The R_t_ (obs) of the known alkaloids in the present study was corrected to compare with the corresponding retention times of our most recent summary (Daly et al.2005). A simple plot with observed vs. corrected retention times of the known alkaloids, selected from several of the tables below, generates a straight line; the corrected retention times for the unknowns are obtained using the observed values and interpolating within this line. Both values, R_t_ (obs.) and R_t_ (corr.), were fairly reproducible among the tables S1-S10 for any repeated alkaloid.

Because of some imprecision in the published GC retention times which were accumulated over many years with a variety of capillary columns, some glass but generally fused-silica columns, we have allowed a generous +/- 0.15 min between the literature value and this study's corrected GC retention times before concluding that an alkaloid is a new alkaloid or a diastereomer of a previously reported alkaloid. These are indicated in Tables S1-S10 by an asterisk after the code or the corrected retention time, respectively.

| Table S1. Data for Toad # 1 ^a^ | | | | |
| --- | --- | --- | --- | --- |
| R_t_ (obs.) | R_t_ (corr.) ^b^ | Alkaloid ^c^ | Class | Comments |
| 4.63 | 3.66 | **169A** * | Unclass | CI 170; C_11_H_23_N |
| 5.96 | 5.02 | **183C** | Unclass | CI 184; C_12_H_25_N; M-CH_3_ = C_11_H_22_N |
| 6.73 | 5.80 | **193N** * | Tri | CI 194; C_13_H_23_N |
| 6.92 | 5.99 | **195G** | 5,6,8-I | CI 196; C_13_H_25_N |
| 7.59 | 6.68* | **207N** | Unclass | CI 208; C_14_H_25_N  IR ν_C=CH-_ 3015 cm^-1^ |
| 8.14 | 7.24 | **207X** * ^d^ | Unclass | C_14_H_25_N |
| 8.29 | 7.39* | **205G** | Iz | C_14_H_23_N |
| 8.74 | 7.85 | **205H** | Tri | CI 206; C_14_H_23_N |
| 8.84 | 7.95 | **193O** * | Tri | CI 194; C_13_H_23_N analog of **193C** |
| 9.05 | 6.17 | **221M** | Tri | CI 222 |
| 9.32 | 8.44 | **223AB** (5*E*,9*Z*) | 3,5-I | CI 224 |
| 9.38 | 8.45 | **221Q** | 5,6,8-I | CI 222 |
| 9.67 | 8.80 | **203B** | Tri | CI 204 |
| 9.75 | 8.92 | **223X** | 5,6,8-I | CI 224 |
| 9.79 | 8.95 | **223H** | 3,5-P |  |
| 10.20 | 9.34 | **221R** | Iz | C_14_H_23_NO |
| 10.43 | 9.58 | **253W** * | Unclass | CI 254 |
| 10.70 | 9.85 | **237A** | PTX | CI 238; C_15_H_27_NO |
| 10.80 | 9.95 | **196** | Q |  |
| 10.82 | 9.96 | **225P** * | Tri |  |
| 11.76 | 10.93 | **251D** | PTX |  |
| 11.88, 12.25 | 11.06 < 11.43 | **251F2** * | Tri | CI 252; C_16_H_29_NO |
| 12.39 | 11.58 | **247Q** * | Unclass |  |
| 12.95 | 12.15 | **247R** * | Tri |  |
| 13.06, 13.69 | 12.26* < 12.90 | **249P** | Unclass |  |
| 13.39 | 12.59 | **267R** | 5,6,8-I |  |
| 13.45 | 12.66 | **281P** * | Unclass |  |
| 13.71 | 12.92 | **277G** | PTX | C_18_H_31_NO |
| 13.81 | 13.02 | **265E2** * | PTX | CI 266; C_16_H_27_NO_2_ |
| 14.08 | 13.29 | **263U*** | Tri | CI 264 |
| 14.24 | 13.46 | **265D2** * | Tri |  |
| 14.42, 14.59 | 13.65 < 13.81 | **291G** | PTX |  |
| 15.26 | 14.46 | **283G** * | Iz | CI 284; HRMS on 2 fragments |
| 16.01 | 15.26 | **309L** * | PTX |  |
| 16.19 | 15.45 | **309A** | PTX |  |
| 16.29, 16.32 | 15.55, 15.58 | **307G** | PTX | IR ν_OH_, 3649, 3542; B.b. at 2798 > 2751; ν_C=C_, 1653 cm^-1^ |
| 16.52 | 15.78 | **307A** | PTX |  |
| 16.64 | 15.91 | **305B** | PTX |  |
| 16.71 | 15.99 | **305I** * ^d^ | PTX | C_19_H_31_NO_2_ |
| 16.84 | 16.12 | **307E** | PTX |  |
| 17.36 | 16.65 | **341A** | aPTX |  |
| 17.45 | 16.74 | **337D** * | PTX | C_20_H_35_NO_2_ |
| 18.07 | 17.37 | **323A** | PTX |  |

^a^ Male, collected 11/21/2007 at El Nogalar, Salta, Argentina.

^b^ GC retention times (R_t_) are within ± 0.15 min. of lit. values.

^c^ Alkaloid codes with an asterisk are previously unreported; a corrected R_t_ with an asterisk indicates an unreported diastereomer.

^d^ A structure has been tentatively proposed. See Fig. 3 (main body).

The symbols < and > indicate relative intensities less than and greater than, respectively. B.b. = Bohlmann band in the IR spectra.

| Table S2. Data for Toad # 2 ^a^ | | | | |
| --- | --- | --- | --- | --- |
| R_t_ (obs.) | R_t_ (corr.) ^b^ | Alkaloid ^c^ | Class | Comments |
| 6.41 | 5.43 | **181F** * ^d^ | Iz | C_12_H_23_N |
| 6.83 | 5.85 | **193N** * | Tri |  |
| 7.04 | 6.07 | **195G** | 5,6,8-I |  |
| 7.58 | 6.61* | **207N** | Unclass | C_14_H_25_N |
| 7.98 | 7.08* | **207J** | Tri | C_14_H_25_N |
| 8.14 | 7.19 | **207X** * ^d^ | Unclass | C_14_H_25_N |
| 8.30 | 7.35* | **205G** | Iz | C_14_H_23_N; deoxy **221R** |
| 8.56 | 7.62 | **203C** * | Iz | C_14_H_21_N;≈ **205G** or **221R** |
| 8.77 | 7.83 | **205H** | Tri |  |
| 9.06 | 8.12 | **223T** | Unclass |  |
| 9.34 | 8.41 | **221Q** | 5,6,8-I |  |
| 9.68 | 8.75 | **203B** | Tri | C_14_H_21_N |
| 10.22, 11.49 | 9.30 > 10.60* | **221R** | Iz | C_14_H_23_NO IR, ν_OH-_ ν_C=CH2_ ν_C=CH-_ 3640, 3072, 3027 cm^-1^; OAc deriv. |
| 10.69 | 9.78 | **237A** | PTX |  |
| 10.80 | 9.90 | **196** | Q |  |
| 11.22 | 10.32 | **203D** * | Unclass | C_13_H_17_NO; no OAc deriv. |
| 11.36 | 10.47 | **235D2** * | Tri | also in #7 |
| 11.75 | 10.86 | **251D** | PTX |  |
| 12.95 | 12.09 | **247R** * | Tri |  |
| 13.39 | 12.53 | **267R** | 5,6,8-I |  |
| 13.70 | 12.85 | **277G** | PTX |  |
| 13.81 | 12.96 | **265E2** * | PTX |  |
| 14.07 | 13.23 | **263U*** | Tri |  |
| 14.24 | 13.40 | **265D2** * | Tri |  |
| 14.47, 14.59 | 13.62 < 13.75 | **291G** | PTX |  |
| 14.47 | 13.63* | **275D** | De-5,8-I | CI 276; C_19_H_33_N |
| 14.77 | 13.94 | **273E** * ^d^ | De-5,8-I | CI 274; C_19_H_31_N |
| 15.26 | 14.46 | **283G** * | Iz | CI 284; HRMS on 2 fragments. |
| 15.57, 15.70 | 14.75, 14.88 (1:1) | **281Q** * ^d^ | 5,8-I | CI 282; C_18_H_35_NO |
| 16.01 | 15.20 | **309L** * | PTX |  |
| 16.18, 16.72 | 15.37 < 15.92 | **305I** * ^d^ | PTX |  |
| 16.29 | 15.48 | **307G** | PTX |  |
| 16.51 | 15.71 | **307A** | PTX |  |
| 16.64 | 15.84* | **305B** | PTX |  |
| 16.85 | 16.05 | **307E** | PTX |  |
| 17.37 | 16.58 | **341A** | aPTX |  |
| 17.46 | 16.68 | **337D** * | PTX | C_20_H_35_NO_3_ |
| 18.07 | 17.30 | **323A** | PTX |  |

^a^ Male, collected 11/21/2007 at El Nogalar, Salta, Argentina.

^b^ GC retention times (R_t_) are within ± 0.15 min. of lit. values.

^c^ Alkaloid codes with an asterisk are previously unreported; a corrected R_t_ with an asterisk indicates an unreported diastereomer.

^d^ A structure has been tentatively proposed. See Fig. 3 (main body).

The symbols < and > indicate relative intensities less than and greater than, respectively.

| Table S3. Data for Toad # 3 ^a^ | | | | |
| --- | --- | --- | --- | --- |
| R_t_ (obs.) | R_t_ (corr.) ^b^ | Alkaloid ^c^ | Class | Comments |
| 6.77, 6.92 | 5.84, 5.99 | **193N** * | Tri |  |
| 7.03 | 6.10 | **195G** | 5,6,8-I |  |
| 7.58 | 6.66 | **207N** | Unclass |  |
| 8.13 | 7.22 | **207X** * ^d^ | Unclass |  |
| 8.30 | 7.39* | **205G** | Iz |  |
| 8.90 | 8.02 | **211U** * | Unclass | related to **211G** |
| 9.06, 9.33, 9.67 | 8.16, 8.43*, 8.79 | **223T** | Unclass |  |
| 9.81 | 8.92 | **221Z** * ^d^ | Tri | C_15_H_27_N |
| 10.21 | 9.33 | **221R** | Iz |  |
| 10.44 | 9.57 | **253W** * | Unclass | C_15_H_27_NO_2_ |
| 10.60 | 9.73 | **231B** | 5,6,8-I |  |
| 10.69 | 9.83 | **237A** | PTX |  |
| 11.74 | 10.89 | **251D** | PTX |  |
| 11.85 | 11.00* | **253O** | Unclass |  |
| 12.95 | 12.13 | **247R** * | Tri |  |
| 13.39 | 12.58 | **267R** | 5,6,8-I |  |
| 13.70 | 12.89 | **277G** | PTX |  |
| 13.81 | 13.00 | **265E2** * | PTX |  |
| 14.07 | 13.27 | **263U*** | Tri |  |
| 14.46, 14.59 | 13.67 < 13.79 | **291G** | PTX |  |
| 15.26 | 14.46 | **283G** * | Iz | CI 284; HRMS on 2 fragments |
| 16.00 | 15.23 | **309L** * | PTX |  |
| 16.17, 16.71 | 15.40 < 15.95 | **305I** * ^d^ | PTX |  |
| 16.28 | 15.52 | **307G** | PTX |  |
| 16.50 | 15.77 | **307A** | PTX |  |
| 16.64 | 15.88 | **305B** | PTX |  |
| 16.84 | 16.08 | **307E** | PTX |  |
| 17.38 | 16.63 | **341A** | aPTX |  |
| 17.45 | 16.71 | **337D** * | PTX |  |
| 18.07 | 17.34 | **323A** | PTX |  |

^a^ Male, collected 11/21/2007 at Huaico Chico, Salta, Argentina.

^b^ GC retention times (R_t_) are within ± 0.15 min. of lit. values.

^c^ Alkaloid codes with an asterisk are previously unreported; a corrected R_t_ with an asterisk indicates an unreported diastereomer.

^d^ A structure has been tentatively proposed. See Fig. 3 (main body).

The symbols < and > indicate relative intensities less than and greater than, respectively.

| Table S4. Data for Toad # 4 ^a^ | | | | |
| --- | --- | --- | --- | --- |
| R_t_ (obs.) | R_t_ (corr.) ^b^ | Alkaloid ^c^ | CIass | Comments |
| 6.00 | 5.03 | **183C** | Unclass | CI 184; C_12_H_25_N |
| 6.85 | 5.90 | **193N*** | Tri | CI 194 |
| 7.04 | 6.10 | **195G** | 5,6,8-I |  |
| 7.80 | 6.87 | **193C** | Tri |  |
| 7.91 | 6.98* | **207J** | Tri |  |
| 8.14 | 7.22 | **207X** * ^d^ | Unclass | C_14_H_25_N; HRMS on 4 fragments |
| 8.33 | 7.41* | **205G** | Iz |  |
| 8.50 | 7.59 | **209T** * | Unclass | related to **223T** |
| 8.85 | 7.94 | **193O** * | Tri |  |
| 9.07, 9.67 | 8.16 < 8.77 | **223T** | Unclass |  |
| 9.19, 9.30 | 8.29 (5*Z*,9*Z*) < 8.40 (5*E*,9*Z*) | **223AB** | 3,5-I |  |
| 9.36 | 8.46 | **221Q** | 5,6,8-I |  |
| 9.67 | 8.77 | **203B** | Tri |  |
| 9.80 | 8.91 | **221Z** * ^d^ | Tri |  |
| 10.20 | 9.32 | **221R** | Iz |  |
| 10.39 | 9,51 | **223E2** * | Tri |  |
| 10.44 | 9.56 | **253W** * | Unclass |  |
| 10.58 | 9.71 | **231B** | 5,6,8-I |  |
| 11.43 | 10.57 | **236** | Spiro |  |
| 11.75 | 10.90 | **251D** | PTX |  |
| 12.56 | 11.73 | **239E2*** ^d^ | 4,6-Q | C_15_H_29_NO; also in # 5, # 6 |
| 12.95 | 12.12 | **247R*** | Tri |  |
| 13.39 | 12.57 | **267R** | 5,6,8-I |  |
| 13.70 | 12.89 | **277G** | PTX |  |
| 13.81 | 13.00 | **265E2** * | PTX |  |
| 14.08 | 13.27 | **263U** * | Tri | CI 264 |
| 14.25 | 13.44 | **265D2** * | Tri | CI 266 |
| 14.46, 14,59 | 13.66 < 13.80 | **291G** | PTX | CI 292, 292 |
| 15.07 | 14.28 | **261I** * | Unclass | CI 262; also in # 6, # 7 |
| 15.26 | 14.46 | **283G** * | Iz | CI 284; HRMS on 2 fragments |
| 15.37 | 14.59 | **277B** | PTX | CI 278 |
| 16.00 | 15.23 | **309L** * | PTX | CI 310; also in # 1-3 |
| 16.16, 16.72 | 15.40 < 15.95 | **305I** * ^d^ | PTX | CI 306, 306; also in # 2, # 3 |
| 16.28 | 15.52 | **307G** | PTX | CI 308 |
| 16.51 | 15.75 | **307A** | PTX | CI 308 |
| 16.64 | 15.88 | **305B** | PTX | CI 306 |
| 16.84 | 16.09 | **307E** | PTX | CI 308 |
| 17.37 | 16.63 | **341A** | aPTX | CI 342 |
| 17.46 | 16.72 | **337D** * | PTX | CI 338 |
| 18.07 | 17.35 | **323A** | PTX |  |
| 24.53 | 23.80 | **374A** * | Unclass | CI 375; also in # 7, # 8 |

^a^ Male, collected 11/21/2007 at Huaico Chico, Salta, Argentina.

^b^ GC retention times (R_t_) are within ± 0.15 min. of lit. values.

^c^ Alkaloid codes with an asterisk have not been reported previously; a corrected R_t_ with an asterisk indicates an unreported diastereomer.

^d^ A structure has been tentatively proposed. See Fig. 3 (main body).

The symbols < and > indicate relative intensities less than and greater than, respectively.

| Table S5. Data for Toad # 5 ^a^ | | | | |
| --- | --- | --- | --- | --- |
| R_t_ (obs.) | R_t_ (corr.) ^b^ | Alkaloid ^c^ | Class | Comments |
| 6.74 | 5.80 | **193N** * | Tri | CI 194 |
| 7.80 | 6.86* | **193G** | 5,6,8-I |  |
| 7.91 | 6.98 | **207J** | Tri |  |
| 8.32 | 7.39 | **221A2** * | Tri |  |
| 8.42 | 7.49 | **209N** | Iz | CI 210 |
| 10.21 | 9.32 | **221R** | Iz |  |
| 10.42 | 9.53 | **225Q** * | Iz | CI 226 |
| 10.69 | 9.80 | **237A** | PTX |  |
| 10.80 | 9.91 | **196** | Q | CI 197 |
| 10.91 | 10.03* | **225B** ^d^ | Pip | CI 226; C_15_H_31_N *trans* isomer |
| 11.75 | 10.88 | **251D** | PTX |  |
| 12.05 | 11.19 | **239D2** * ^d^ | 5,6,8-I | CI 240; C_15_H_29_NO |
| 12.56 | 11.70 | **239E2** * ^d^ | 4,6-Q |  |
| 13.38 | 12.54 | **267R** | 5,6,8-I |  |
| 14.07 | 13.24 | **263U** * | Tri |  |
| 14.24 | 13.42 | **265D2** * | Tri |  |
| 14,57 | 13.75 | **291G** | PTX |  |
| 15.26 | 14.48 | **283G** * | Iz | CI 284; HRMS on 2 fragments |
| 15.37 | 14.57 | **277B** | PTX |  |
| 16.27 | 15.48 | **307G** | PTX |  |
| 17.37 | 16.60 | **341A** | aPTX |  |
| 17.55 | 16.78 | **379** ^e^ | PTX | CI 380 |
| 18.09 | 17.35 | **323A** | PTX | CI 324 |

^a^ Male, collected 1/8/2008 at Los Paños, Jujuy, Argentina.

^b^ GC retention times (R_t_) are within ± 0.15 min of lit values.

^c^ Alkaloids with an asterisk are previously unreported; a corrected R_t_ with an asterisk indicates a previously unreported diastereomer.

^d^ A structure has been tentatively proposed. See Fig. 3 (main body).

^e^ Dimethylsiloxane derivative of **323A**; a GC artifact.

| Table S6. Data for Toad # 6 ^a^ | | | | |
| --- | --- | --- | --- | --- |
| R_t_(obs.) | R_t_(corr.) ^b^ | Alkaloid ^c^ | Class | Comments |
| 6.80, 6.97 | 5.75, 6.05 | **193N*** | Tri |  |
| 7.60 | 6.58 | **195N*** ^d^ | Iz | CI 196 |
| 7.92 | 6.90* | **195G** | 5,6,8-I | CI 196 |
| 7.92 | 6.90* | **207J** | Tri | CI 208 |
| 8.47 | 7.46 | **209N** | Iz |  |
| 9.11, 9.73 | 8.20, 8.76* | **223T** | Unclass | CI 224 |
| 10.32 | 9.37 | **237W** * | Unclass | CI 238 |
| 10.48, 10.52 | 9.54, 9.58 | **225Q** * | Iz | CI 226 |
| 10.59 | 9.65* | **211P** | Iz | CI 212; C_13_H_25_NO; analog of **247R** (# 3) |
| 10.80 | 9.87 | **196** | Q |  |
| 10.98 | 10.05* | **225B** ^d^ | Pip | CI 226; likely *trans* |
| 11.24 | 10.32 | **267Z** * | Unclass | CI 268; C_16_H_29_NO_2_ |
| 11.46 | 10.55 | **236** | Spiro |  |
| 11.75 | 10.84 | **251D** | PTX |  |
| 12.05 | 11.15 | **239D2** * ^d^ | 5,6,8-I | CI 240; C_15_H_29_NO |
| 12.30 | 11.41* | **237O** | Tri | C_15_H_27_NO |
| 12.52 | 11.70 | **247S** * | Unclass | CI 248 |
| 12.63 | 11.75 | **239E2** * ^d^ | 4,6-Q | CI 240 |
| 12.96 | 12.16 | **247R** * | Tri | CI 248 |
| 14.08 | 13.24 | **263U** * | Tri | CI 264 |
| 14.26 | 13.43 | **265D2** * | Tri | CI 266 |
| 14.57 | 13.75 | **291G** | PTX |  |
| 15.06 | 14.25 | **261I** * | Unclass | CI 262; C_17_H_27_NO; also in # 4, # 7 |
| 16.28 | 15.50 | **307G** | PTX |  |
| 18.07 | 17.35 | **323A** | PTX |  |

^a^ Male, collected 1/8/2008 at Los Paños, Jujuy, Argentina.

^b^ GC retention times (R_t_) are within ± 0.15 min. of lit. values.

^c^ Alkaloid codes with an asterisk are previously unreported; a corrected R_t_ with an asterisk indicates an unreported diastereomer.

^d^ A structure has been tentatively proposed. See Fig. 3 (main body).

| Table S7. Data for Toad # 7 ^a^ | | | | |
| --- | --- | --- | --- | --- |
| R_t_(obs.) | R_t_(corr.) ^b^ | Alkaloid ^c^ | CIass | Comments |
| 5.91 | 5.00 | **183C** | Unclass |  |
| 7.69 | 6.80 | **209T** | Unclass | analog of **223T** |
| 8.00 | 7.11 | **207Y** * | Tri |  |
| 8.53 | 7.65 | **199B** * | Unclass | CI 200 |
| 8.76 | 7.88 | **205H** | Tri | CI 206; C_14_H_23_N |
| 8.92 | 8.04 | **193O** * | Tri |  |
| 9.04 | 8.17 | **221S** | Tri | CI 222; C_15_H_27_N; also in # 8 |
| 9.35 | 8.47 | **221Q** | 5,6,8-I | CI 222; C_15_H_27_N |
| 9.35 | 8.47 | **223X** | 5,6,8-I | CI 224; C_15_H_29_N |
| 9.57 | 8.70* | **235E** | 5,6,8-I | CI 236 |
| 9.69 | 8.80* | **203B** | Tri | CI 204; |
| 9.80 | 8.93 (*cis*) | **223H** | 3,5-P | CI 224 |
| 10.07 | 9.21 | **205M** * | Tri | CI 206; C_14_H_23_N |
| 10.22 | 9.36 | **205K** | Tri | C_14_H_23_N |
| 10.22 | 9.36 | **221R** | Iz | co-chrom. with **205K** |
| 10.31 | 9.45 | **207U** | Tri | CI 208; C_13_H_21_NO |
| 10.45 | 9.59 (*trans*) | **223H** | 3,5-P | CI 224 |
| 10.61 | 9.75 | **207Z** * | Tri | CI 208; C_14_H_25_N |
| 11.08 | 10.22 | **221B2** * | Tri | CI 222 |
| 11.36 | 10.50 | **235D2** * | Tri | CI 236; also in #2 |
| 11.75 | 10.91 | **251D** | PTX | CI 252 |
| 11.87, 12.01, 12.61 | 11.03*, 11.17, 11.77* | **251S** | 5,6,8-I | C_16_H_29_NO |
| 12.23 | 11.39* | **237O** | Tri | CI 238; C_15_H_27_NO; also in # 6, # 8 |
| 12.27, 13.33 | 11.56* | **261D** ^d^ | 5,8-I | CI 262; C_18_H_31_N |
| 12.56 | 11.72 | **231J** | Unclass | CI 232; C_16_H_25_N; *m/z* 146, C_10_H_12_N; *m/z* 131, C_9_H_9_N |
| 12.73 | 11.90 | **249C2** * | Unclass | CI 250; C_16_H_27_NO |
| 12.95 | 12.10 | **247R** * | Tri | CI 248; C_17_H_29_N |
| 13.07, 13.69 | 12.23* < 12.87 | **249P** | Unclass | C_16_H_27_NO |
| 13.39 | 12.56 | **267R** | 5,6,8-I | CI 268; C_17_H_33_NO; HRMS *m/z* 168, C_10_H_18_NO |
| 14.08 | 13.26 | **263U** * | Tri | CI 264 |
| 14.25 | 13.43 | **265D2** * | Tri | CI 266 |
| 14.98 | 14.22 | **263W** * | Tri | CI 264 |
| 15.03 | 14.22 | **263V** * | Unclass | CI 264; C_17_H_29_NO |
| 15.06 | 14.25 | **261I** * | Unclass | CI 262; C_17_H_27_NO |
| 15.37 | 14.56 | **277B** | PTX | CI 278; C_17_H_27_NO_2_ |
| 16.29 | 15.49 | **307G** | PTX | CI 308 |
| 17.54 | 16.76 | **379** ^e^ | PTX | CI 380 |
| 18.19 | 17.49 | **323A** | PTX |  |
| 23.31, 23.58 | 22.59 > 22.86 | **380A** * | Unclass | CI 381, 381; C_26_H_40_N_2_; DHQ- dimers? |
| 23.58 | 23.80 | **374A** * | Unclass | CI 375; also in # 4, # 8 |

^a^ Male, collected 12/9/2008 at Tablada, Salta, Argentina.

^b^ GC retention times (R_t_) are within ± 0.15 min. of lit. values.

^c^ Alkaloids with an asterisk are previously unreported; a corrected R_t_ with an asterisk indicates an unreported diastereomer.

^d^ A structure has been tentatively proposed. See Fig. 3 (main body).

^e^ Dimethylsiloxane derivative of **323A**; a GC artifact.

The symbols < and > indicate relative intensities less than and greater than, respectively.

| Table S8. Data for Toad # 8 ^a^ | | | | |
| --- | --- | --- | --- | --- |
| R_t_(obs.) | R_t_(corr.) ^b^ | Alkaloid ^c^ | CIass | Comments |
| 6.87 | 5.97 | **195G** | 5,6,8-I | CI 196 |
| 8.00 | 7.11 | **207Y** * | Tri | CI 208 |
| 8.76 | 7.88 | **205H** | Tri | CI 206 |
| 8.91 | 8.03 | **235E2** * | Unclass | C_16_H_29_N |
| 9.06 | 8.19* | **221S** | Tri | CI 222; C_15_H_27_N, also in # 7 |
| 9.36 | 8.49 | **221Q** | 5,6,8-I | CI 222; C_15_H_27_N |
| 9.67 | 8.80 | **203B** | Tri | CI 204 |
| 10.31 | 9.45 | **207U** | Tri | CI 208 |
| 10.71 | 9.85 | **237A** | PTX | CI 238 |
| 11.18 | 10.33 | **241J** * ^d^ | Amine | C_16_H_35_N |
| 11.75 | 10.91 | **251D** | PTX | CI 252; |
| 12.00 | 11.15 | **251S** | 5,6,8-I | CI 252; C_16_H_29_NO |
| 12.25 | 11.44* | **237O** | Tri | CI 238 |
| 12.39 | 11.58* | **261D** ^d^ | 5,8-I | CI 262; C_18_H_31_N |
| 12.56 | 11.72 | **231J** | Unclass | CI 232 |
| 12.72 | 11.89 | **249D2** * | Unclass | CI 250; also in # 7 |
| 12.97 | 12.14 | **247R** * | Tri | CI 248 |
| 13.06, 13.69 | 12.23*, 12,87 | **249P** | Unclass | CI 250, 250 |
| 14.08 | 13.26 | **263U** * | Tri | CI 264 |
| 14.24 | 13.43 | **265D2** * | Tri | CI 266 |
| 14.97 | 14.21 | **263W** * | Tri | CI 264; C_17_H_29_NO |
| 15.03 | 14.22 | **263V** * | Unclass | CI 264 |
| 15.06 | 14.25 | **261I** * | Unclass | CI 262 |
| 15.26 | 14.46 | **283G** * | Iz | CI 284; HRMS on 2 fragments |
| 15.37 | 14.56 | **277B** | PTX | CI 278; mixed with CI 290 |
| 16.28 | 15.48 | **307G** | PTX | CI 308 |
| 16.38 | 15.59 | **309A** | PTX | CI 310 |
| 16.48 | 15.75 | **307A** | PTX |  |
| 16.71 | 15.92 | **305I** * ^d^ | PTX | CI 306; also in # 2 |
| 17.37 | 16.58 | **341A** | aPTX | CI 342 |
| 17.54 | 16.76 | **379** ^e^ | PTX | CI 380 |
| 17.61 | 16.84 | **321F** * ^d^ | PTX | C_19_H_31_NO_3_; related to **323A** |
| 18.06 | 17.28 | **323A** | PTX | CI 324 |
| 24.52 | 23.80 | **374A** * | Unclass |  |

^a^ Female, collected 12/9/2008 at Tablada, Salta, Argentina.

^b^ GC retention times (R_t_) are within ± 0.15 min. of lit. values.

^c^ Alkaloid codes with an asterisk are previously unreported; a corrected R_t_ with an asterisk indicates an unreported diastereomer.

^d^ A structure has been tentatively proposed. See Fig. 3 (main body).

^e^ Dimethylsiloxane derivative of **323A**; a GC artifact.

| Table S9. Data for Toad # 9 ^a^ | | | | |
| --- | --- | --- | --- | --- |
| R_t_(obs.) | R_t_(corr.) ^b^ | Alkaloid ^c^ | Class | Comments |
| 6.65 | 5.60* | **195L** | Unclass | CI 196; C_13_H_25_N |
| 7.41, 7.59 | 6.37***,** 6.56* | **207N** | Unclass | CI 208, 208 |
| 7.83 | 6.81 | **193C** | Tri | CI 194 |
| 8.89, 9.04. 9.29. 9.65 | 7.92*, 8.06, 8.32*, 8.69, | **223T** | Unclass | CI 224, 224, 224, 224 |
| 9.23 | 8.25 | **221Z** * ^d^ | Tri | CI 222; C_15_H_27_N |
| 10.23 | 9.29 | **225O** * | Unclass | CI 226; C_14_H_27_NO |
| 10.44 | 9.49 | **253W** * | Unclass | CI 254; C_15_H_27_NO_2_; also HRMS on fragments |
| 10.70 | 9.76 | **237A** | PTX | CI 238 |
| 10.79 | 9.85 | **196** | Q | CI 197 |
| 11.02 | 10.08 | **239F2** * ^d^ | Amine | CI 240; C_15_H_29_NO |
| 11.66 | 10.75* | **253O** | Unclass | CI 254; C_15_H_27_NO_2_;; HRMS on 2 fragments |
| 11.74 | 10.83 | **251D** | PTX | CI 252 |
| 12.38, 12.94 | 11.49, 12.07 | **267A2** * | Unclass | CI 268, 268; C_15_H_25_NO_3_ |
| 14.57 | 13.74 | **291G** | PTX | CI 292 |
| 15.26 | 14.46 | **283G** * | Iz | CI 184; HRMS on 2 fragments |
| 15.36 | 14.56 | **277B** | PTX | CI 278 |
| 16.28 | 15.50 | **307G** | PTX | CI 308 |
| 17.39 | 16.64 | **341A** | aPTX | IR ν_OH_ 3569, 3550 cm^-1^; B.b. at 2817 cm^-1^ |
| 17.62 | 16.88 | **348A** * | Unclass | CI 349; C_22_H_40_N_2_O |
| 18.08 | 17.35 | **323A** | PTX |  |
| 18.83 | 18.13 | **364A** * | Unclass | CI 365; C_22_H_40_N_2_O_2_ |

^a^ Male, collected 1/18/2008 at El Cucho, Jujuy, Argentina.

^b^ GC retention times (R_t_) are within ± 0.15 min. of lit. values.

^c^ Alkaloid codes with an asterisk are previously unreported; a corrected R_t_ with an asterisk indicates an unreported diastereomer.

^d^ A structure has been tentatively proposed. See Fig. 3 (main body).

B.b. = Bohlmann band in the IR spectra.

| Table S10. Data for Toad # 10 ^a^ | | | | |
| --- | --- | --- | --- | --- |
| R_t_(obs.) | R_t_(corr.) ^b^ | Alkaloid ^c^ | Class | Comments |
| 5.96 | 5.06* | **183C** | Unclass | CI 184; C_12_H_25_N |
| 6.35 | 5.41 | **181F** * ^d^ | Iz | CI 182; C_12_H_23_N |
| 6.65 | 5.60* | **195L** | Unclass | CI196; C_13_H_25_N; also in # 9 |
| 7.42 | 6.38* | **207N** | Unclass | CI 208 |
| 7.85 | 6.83 | **193C** | Tri | CI 194 |
| 8.25 | 7.24 | **209B** | 5,8-I | CI 210; C_14_H_27_N |
| 8.79 | 7.80 | **193O** * | Tri | CI 194 |
| 8.91, 9.05, 9.26, 9.66 | 7.92*, 8.06, 8.30*, 8.69 | **223T** | Unclass | CI 224; C_15_H_29_N (for all four) |
| 9.23 | 8.24 | **221Z** * ^d^ | Tri | CI 222; C_15_H_27_N |
| 9.81 | 8.84 (*cis*) | **223H** | 3,5-P | CI 224 |
| 10.28 | 9.33 | **225O** * | Unclass | CI 226; C_14_H_27_NO |
| 10.44 | 9.49 | **253W** * | Unclass | CI 254 |
| 10.70 | 9.76 | **237A** | PTX | CI 238 |
| 10.80 | 9.87 | **196** | Q | CI 197 |
| 11.02 | 10.09 | **239F2** * ^d^ | Unclass | CI 240 |
| 11.23 | 10.31 | **267Z** * | Unclass | CI 240; C_16_H_29_NO_2_; HRMS also on fragments |
| 11.68 | 10.77* | **253O** | Unclass | CI 254 |
| 11.75 | 10.84 | **251D** | PTX | CI 252 |
| 12.01 | 11.11 | **267B2** * | Unclass | CI 268; analog of **267Z** |
| 12.38, 12.94 | 11.49, 12.06 | **267A2** * | Unclass | CI 268, 268; C_15_H_25_NO_3_; both isomers also in # 9 |
| 17.40 | 16.65 | **341A** | aPTX |  |
| 17.63 | 16.89 | **348A** * | Unclass | CI 349; C_22_H_40_N_2_O; also in # 9 |
| 18.09 | 17.37 | **323A** | PTX |  |

^a^ Male, collected 1/18/2008 at El Cucho, Jujuy, Argentina.

^b^ GC retention times (R_t_) are within ± 0.15 min. of lit. values.

^c^ Alkaloid codes with an asterisk are previously unreported; a corrected R_t_ with an asterisk indicates an unreported diastereomer.

^d^ A structure has been tentatively proposed. See Fig. 3 (main body).

| Table S11 Collection Information on Skins of *Melanophyriscus rubriventris* | | | | | | |
| --- | --- | --- | --- | --- | --- | --- |
| Skin # | Voucher^a^ | Sex,  SVL (mm) | Site (locality) | Collection Date | GPS coordinates (lat./long.) | Elevation (m) |
| 1 | MV 512 | m, 39.3 | El Nogalar, Salta  (Los Toldos) | 11-21-2007 | S 22° 16.737′  W 64° 43.107′ | 1635 |
| 2 | 516 | m, 37.6 |
| 3 | 543 | m, 37.2 | Huaico Chico, Salta  (Nacientes) | 11-21-2007 | S 22° 16.365′  W 64° 42.590′ | 1715 |
| 4 | 546 | m, 35.7 |
| 5 | - | m, not det. | Los Paños, Jujuy  (La Almona) | 1-8-2008 | S 24° 18.206′  W 65° 24.968′ | 1696 |
| 6 | - | m, not det. |
| 7 | 692 | m. 36 | Tablada, Salta  (Chorro Negro) | 12-9-2008 | S 23° 5.153′  W 64° 51.720′ | 1725 |
| 8 | 703 | f, 38.3 |
| 9 | 529 | m, 37.9 | El Cucho, Jujuy  (Finca Los Bolsones) | 1-18-2008 | S 24° 4.936′  W 65° 11.875′ | 1271 |
| 10 | 533 | m, 33.7 |

^a^ Voucher samples with these codes are deposited with Museo de Ciencias Naturales de la Universidad Nacional de Salta, Argentina.

Below are tabulated the GC-MS total ion currents observed with each toad sample.

| Table S12. GC-EIMS Ion Current Intensities | |
| --- | --- |
| Skin # | Total Ion Current  (x 10^7^) |
| 1 | 3.72 |
| 2 | 5.75 |
| 3 | 3.60 |
| 4 | 3.71 |
| 5 | 2.98 |
| 6 | 1.69 |
| 7 | 9.21 |
| 8 | 4.10 |
| 9 | 2.96 |
| 10 | 3.41 |

**Molecular formulae by HRMS and selected HRMS data on 25 new alkaloids and some MS fragments (previously unreported) from three known alkaloids (205K, 223X, and 275D) detected in Melanophryniscus rubriventris:**

**181F**. Iz C_12_H_23_N. R_t_ 5.43. HRMS: *m/z* 124 (C_8_H_14_N).

**203C**. Iz. C_14_H_21_N. R_t_ 7.62. HRMS: *m/z* 146 (15, C_10_H_12_N), 134 (100, C_9_H_12_N).

**203D**. Unclass. C_13_H_17_NO. R_t_ 10.32. HRMS: *m/z* 188 (C_12_H_14_NO); 160 (C_11_H_12_O); 159 (C_11_H_11_O).

**205K**. Tri. C_14_H_23_N. R_t_ 9.36. HRMS: *m/z* 134 (C_9_H_12_N).

**207X**. Unclass. C_14_H_25_N. R_t_ 7.19. HRMS: *m/z* 164 (20, C_11_H_18_N), 152 (100, C_10_H_18_N), 136 (47, C_9_H_14_N), 134 (40, C_9_H_12_N).

**207Z**. Tri. C_14_H_25_N. R_t_ 9.75. HRMS: *m/z* 192 (78, C_13_H_22_N), 178 (100, C_12_H_20_N), 164 (88, C_11_H_18_N), 138 (84, C_9_H_16_N), 110 (96, C_7_H_12_N), 58 (56, C_3_H_8_N).

**223X**. 5,6.8-I. C_15_H_29_N. R_t_ 8.92.

**225O**. Unclass. C_14_H_27_NO. R_t_ 9.31. HRMS: *m/z* 194 (100, C_13_H_24_N).

**241J**. Amine. C_16_H_35_N. R_t_ 10.33. HRMS: *m/z* 138 (16, C_9_H_16_N).

**249P**. Unclass. C_16_H_27_NO. R_t_ 11.90. HRMS: *m/z* 151 (C_10_H_17_N).

**249C2**. Unclass. C_16_H_27_NO. R_t_ 11.90. HRMS: *m/z* 234 (26, C_15_H_24_NO), 190 (100, C_13_H_20_N), 150 (80, C_10_H_16_N), 148 (92, C_10_H_14_N).

**251F2**. Tri. C_16_H_29_NO. R_t_ 11.43 (maj. isomer). HRMS: *m/z* 164 (70, C_11_H_18_N), 150 (46, C_10_H_16_N).

**253W**. Unclass. C_15_H_27_NO_2_. R_t_ 9.49. HRMS: *m/z* 194 (92, C_13_H_24_N), 193 (63, C_13_H_23_N), 178(50, C_12_H_20_N).

**261I**. Unclass. C_17_H_27_NO. R_t_ 14.25. HRMS: *m/z* 218 (22, C_15_H_24_N), 204 (100, C_14_H_22_N).

**263V.** Unclass. C_17_H_29_NO. R_t_ 14.22. HRMS: *m/z* 234 (22, C_15_H_24_NO), 204 (100, C_14_H_22_N), 151 (40, C_10_H_17_N).

**263W**. Tri. C_17_H_29_NO. R_t_ 14.21. HRMS: *m/z* 248 (20, C_16_H_26_NO), 246 (40, C_17_H_28_N), 220 (62, C_14_H_22_NO), 190 (100, C_13_H_20_N).

**267Z**. Unclass. C_16_H_29_NO_2_. R_t_ 10.32. HRMS: *m/z* 252 (92, C_15_H_26_NO_2_), 166 (58, C_11_H_20_N), 150 (100, C_10_H_16_N).

**267A2**. Unclass. C_15_H_25_NO_3_. R_t_ 11.49 ≈ 12.07. HRMS: *m/z* 224 (100, C_12_H_18_NO_3_).

**273E**. De-5,8-I. C_19_H_31_N. R_t_ 14.00. HRMS: *m/z* 244 (6, C_17_H_26_N), 202 (2, C_14_H_20_N), 150 (100, C_10_H_16_N), 148 (73, C_10_H_14_N), 120 (20, C_8_H_10_N).

**275D**. De-5,8-I. C_19_H_33_N. R_t_ 13.63 (new isomer). HRMS: *m/z* 275 (5, C_19_H_32_N), 260 (4), 246 (7), 231 (5), 151 (10), 150 (100, C_10_H_16_N), 148 (46, C_10_H_14_N), 120 (10, C_8_H_10_N).

**281Q**. 5,8-I. C_18_H_35_NO. R_t_ 14.75. HRMS: *m/z* 138 (100, C_9_H_16_N).

**283G**. Iz. (M**^+.^** not detected, ‘C_17_H_33_NO_2_’). R_t_ 14.46. HRMS: *m/z* 184 (100, C_10_H_18_NO_2_), 126 (46, C_7_H_12_NO).

**305I**. PTX. C_19_H_31_NO_2_. R_t_ 15.99. HRMS: *m/z* 262 (66, C_17_H_28_NO), 247 (100, C_16_H_25_NO), 206 (86, C_13_H_20_NO).

**321F**. PTX. C_19_H_31_NO_3_. R_t_ 16.84. HRMS *m/z* 193 (100, C_12_H_19_NO).

**348A**. Unclass. C_22_H_40_N_2_O. R_t_ 16.88. HRMS: *m/z* 304 (65, C_20_H_34_NO), 288 (40, C_19_H_30_NO), 260 (45, C_18_H_30_N), 234 (100, C_15_H_24_NO), 218 (44, C_14_H_22_N_2_), 143 (40, C_8_H_19_N_2_), 114 (70, C_7_H_16_N).

**364A**. Unclass. C_22_H_40_N_2_O_2_. R_t_ 18.13. HMS: *m/z* 346 (44, C_22_H_38_N_2_O), 302 (36, C_20_H_32_NO), 143 (44, C_8_H_19_N_2_), 114 (75, C_7_H_16_N).

**374A**. Unclass. (M**^+.^** not detected, ‘C_23_H_38_N_2_O_2_’. R_t_ 23.80. HRMS: *m/z* 261 (70, C_17_H_13_N_2_O), 230 (26), 229 (100, C_16_H_9_N_2_), 82 (10, C_5_H_8_N).

**380A**. Unclass. C_26_H_40_N_2_.

| Table S13. Effect of Proximity between 2002 and 2008 collections-Salta Province | | | | | | | | |
| --- | --- | --- | --- | --- | --- | --- | --- | --- |
| Alkaloid^a^ | Cedral de Baritú  2002 (A) | Canto del Monte  2002 (B) | R.N. El  Nogalar  # 1-2008 | R.N. El  Nogalar  # 2-2008 | Huaico Chico  # 3-2008 | Huaico Chico  # 4-2008 | Tablada  # 7-2008 | Tablada  # 8-2008 |
| **183C** |  | x | x |  |  | x | x |  |
| **191E** |  | x |  |  |  |  |  |  |
| **195G** | x | x | x | x | x | x |  | x |
| **195L** |  | x |  |  |  |  |  |  |
| **203B** | x | x | x | x |  | x | x | x |
| **205G** | x |  | x | x | x | x |  |  |
| **205H** (2) | x | x/x | x | x |  |  | x | x |
| **207C** |  | x |  |  |  |  |  |  |
| **207O** |  | x |  |  |  |  |  |  |
| **207U** | x |  |  |  |  |  | x | x |
| **209M** (2) |  | x/x |  |  |  |  |  |  |
| **221Q** |  | x | x | x |  | x | x | x |
| **221R** (2) | x |  | x | x/x | x | x | x |  |
| **221S** |  | x | x |  |  |  | x | x |
| **223T** (2) |  | x/x |  | x | x/x | x/x |  |  |
| **223AB** |  | x (*Z,Z*) | x (*Z,Z*) |  |  | x/x (*Z,Z*)/ (*E,Z*) |  |  |
| **237A** | x | x | x | x | x |  |  | x |
| **251D** |  | x | x | x | x | x | x | x |
| **277B** |  | x |  |  |  | x | x | x |
| **277G** |  | x | x | x | x | x |  |  |
| **289C** | x | x |  |  |  |  |  |  |
| **291G** (2) | x | x | x/x | x/x | x/x | x/x |  |  |
| **307A** | x | x | x | x | x | x |  | x |
| **307G** (2) | x | x | x/x | x | x | x | x | x |
| **309A** | x | x | x |  |  |  |  | x |
| Totals | 12 | 25 | 18 | 15 | 12 | 17 | 10 | 12 |
| Current alkaloids shared with A | | | 10 | 9 | 7 | 7 | 5 | 7 |
| Current alkaloids shared with B | | | 13 | 10 | 9 | 12 | 8 | 11 |

^a^ “(2)” indicates two diastereomers were detected; both were counted.

| Table S14. Effect of Proximity between 2002 and 2008 collections-Jujuy Province | | | | | | |
| --- | --- | --- | --- | --- | --- | --- |
| Alkaloid^a^ | Abra Colorada  2002 (A) | Tiraxi  2002 (B) | El Cucho  # 9-2008 | El Cucho  # 10-2008 | Los Paños  # 5-2008 | Los Paños  # 6-2008 |
| **183C** |  | x |  | x |  |  |
| **191D** | x |  |  |  |  |  |
| **193C** (2) |  | x/x | x | x |  |  |
| **195G** | x | x |  |  |  | x |
| **205F** | x |  |  |  |  |  |
| **207T** (2) | x/x |  |  |  |  |  |
| **209N** |  | x |  |  | x | x |
| **221Q** |  | x |  |  |  |  |
| **221R** |  | x |  |  | x |  |
| **221W** | x |  |  |  |  |  |
| **237O** |  | x/x |  |  |  | x |
| **251D** |  | x | x | x | x | x |
| **261D** (2) |  | x/x |  |  |  |  |
| **267A** |  | x |  |  |  |  |
| **267R** |  | x |  |  | x |  |
| **273C** | x |  |  |  |  |  |
| **275H** (2) |  | x/x |  |  |  |  |
| **277B** (2) | x | x/x | x |  | x |  |
| **291G** |  | x | x |  | x | x |
| **307G** (2) | x | x/x | x |  | x | x |
| **319F** |  | x |  |  |  |  |
| **323A** | x | x | x | x | x | x |
| Totals | 10 | 23 | 6 | 4 | 8 | 7 |
| Current alkaloids shared with A | | | 3 | 1 | 3 | 3 |
| Current alkaloids shared with B | | | 6 | 4 | 8 | 7 |

^a^ “(2)” indicates two diastereomers were detected; both were counted.
