# Supplementary figures and images for: Alkaloids from single skins of the Argentinian toad Melanophryniscus rubriventris (ANURA, BUFONIDAE): An unexpected variability in alkaloid profiles and a profusion of new structures
Source: Springerplus. 2012 Nov 23;1(1):51. doi: 10.1186/2193-1801-1-51 (PMC3625416; doi:10.1186/2193-1801-1-51)

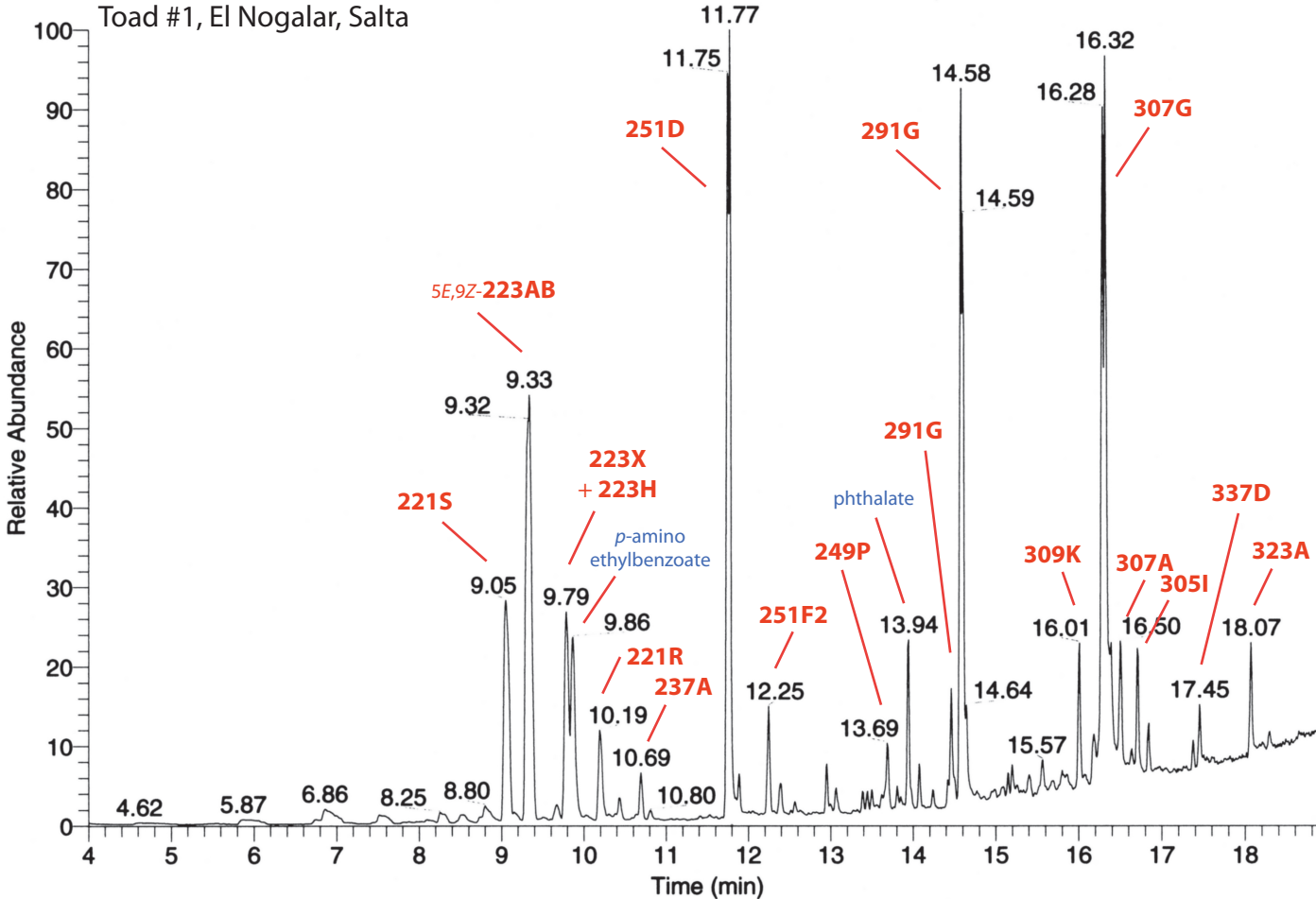

Supplement: Supplementary file 2 — Additional file 2 Figure S27.: Typical ponds where collections were made: a) Cedral de Baritú, Salta; b) Los Paños, Jujuy. The blue parallelogram indicates the average collection area. (ZIP 9 MB) [file 40064_2012_198_MOESM2_ESM.zip › add2/1118854145799791_fig1.pdf]

Toad #10, Cucho, Jujuy

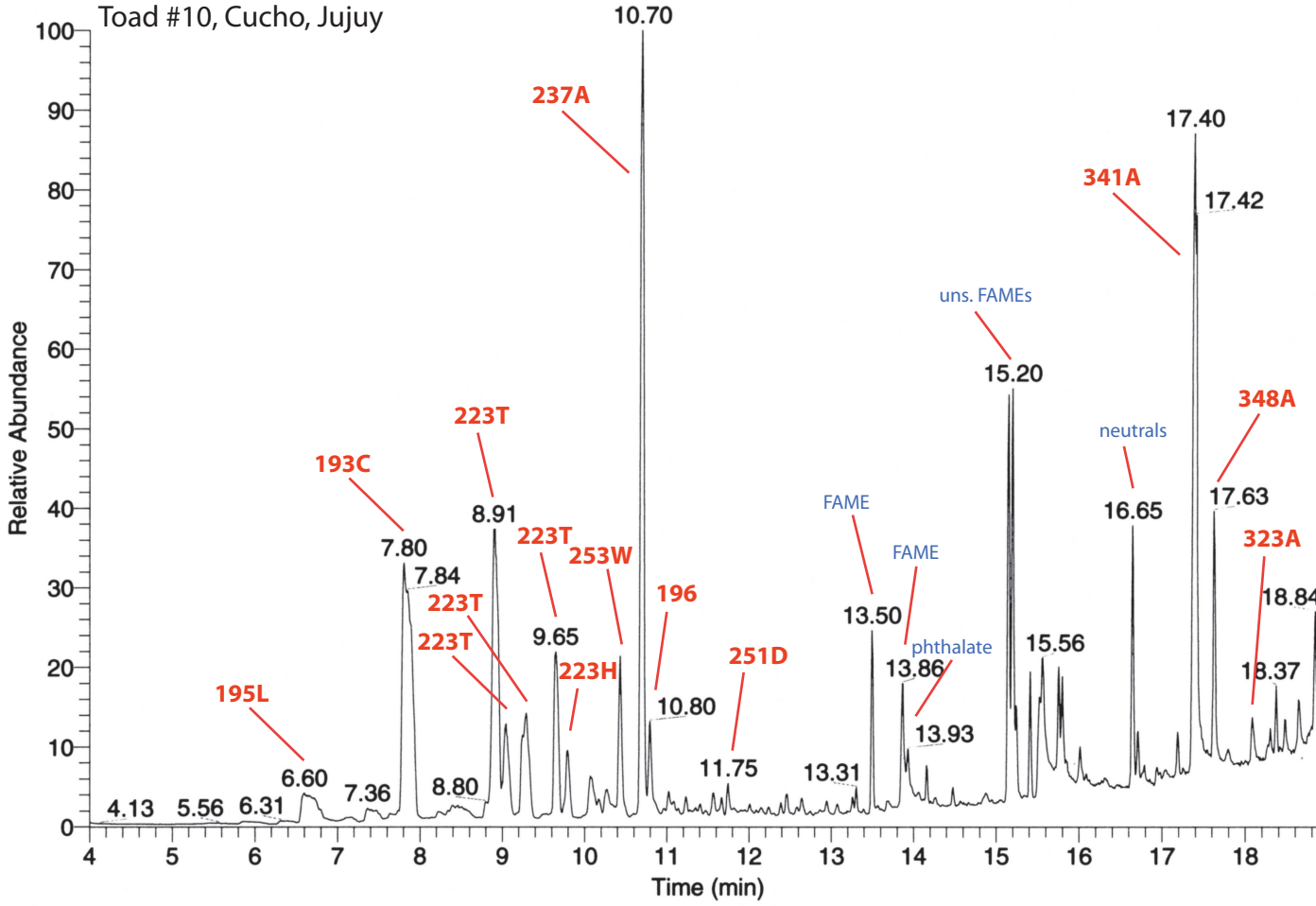

Supplement: Supplementary file 2 — Additional file 2 Figure S27.: Typical ponds where collections were made: a) Cedral de Baritú, Salta; b) Los Paños, Jujuy. The blue parallelogram indicates the average collection area. (ZIP 9 MB) [file 40064_2012_198_MOESM2_ESM.zip › add2/1118854145799791_fig10.pdf]

Toad #2, El Nogalar, Salta

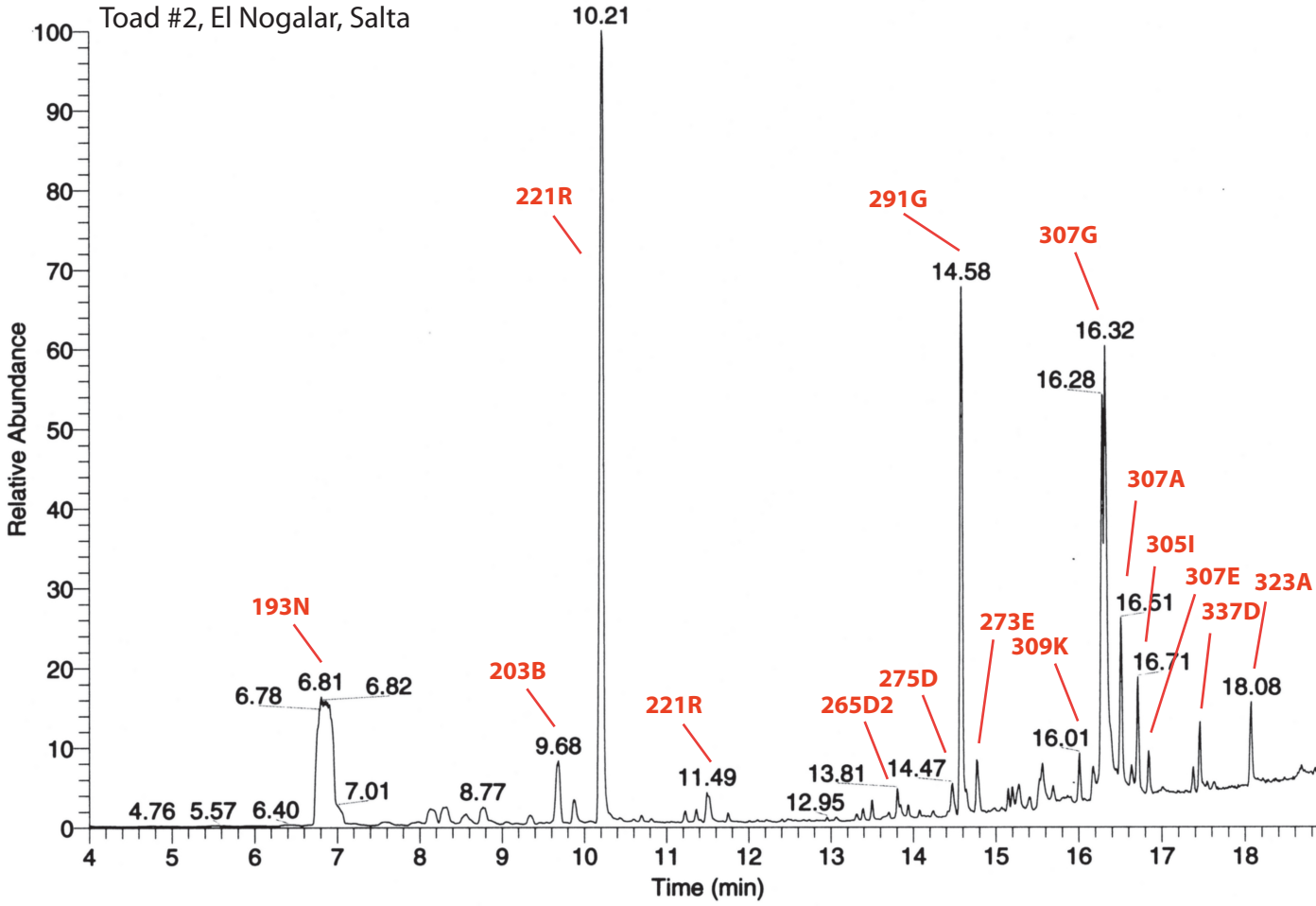

Supplement: Supplementary file 2 — Additional file 2 Figure S27.: Typical ponds where collections were made: a) Cedral de Baritú, Salta; b) Los Paños, Jujuy. The blue parallelogram indicates the average collection area. (ZIP 9 MB) [file 40064_2012_198_MOESM2_ESM.zip › add2/1118854145799791_fig2.pdf]

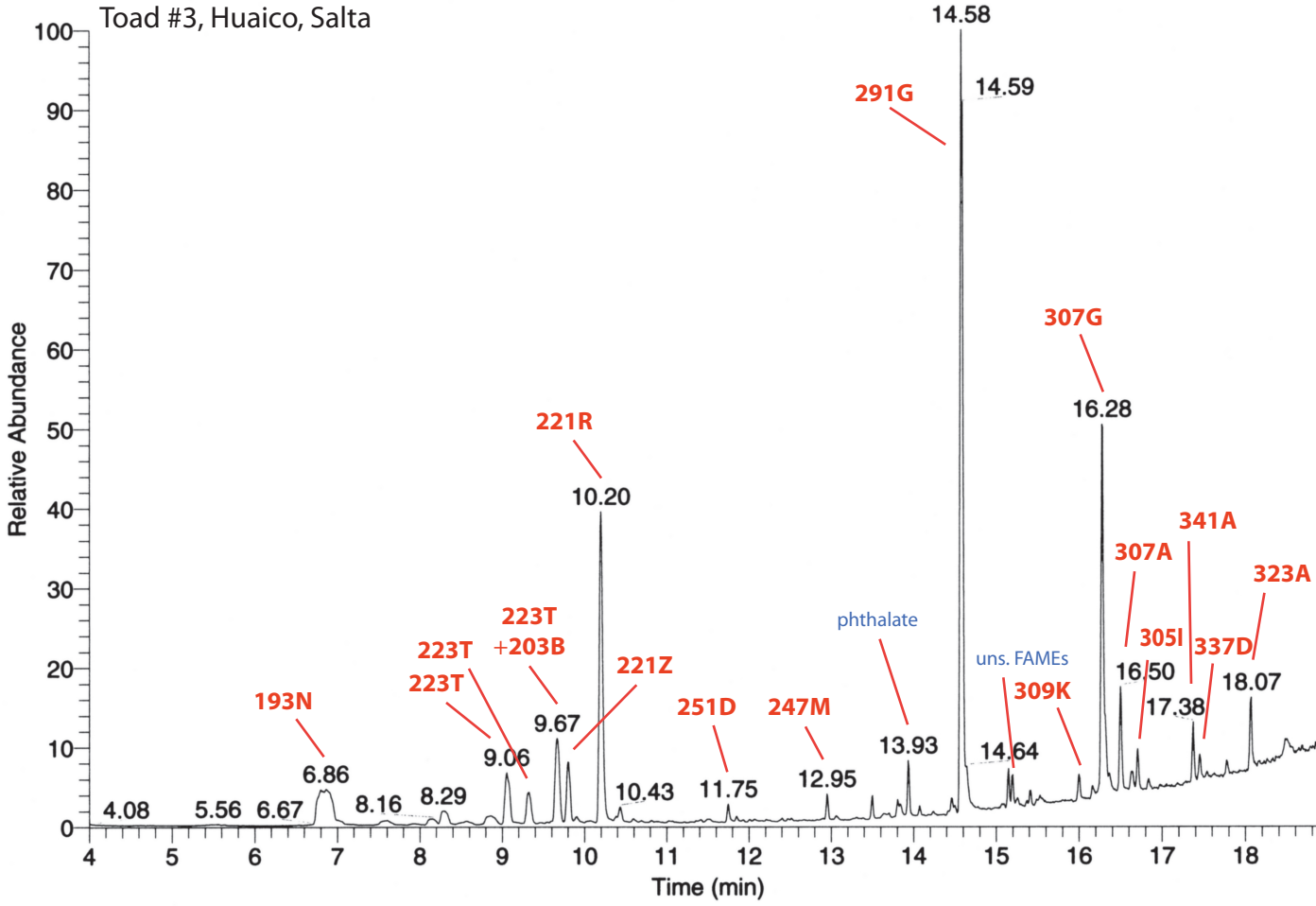

Supplement: Supplementary file 2 — Additional file 2 Figure S27.: Typical ponds where collections were made: a) Cedral de Baritú, Salta; b) Los Paños, Jujuy. The blue parallelogram indicates the average collection area. (ZIP 9 MB) [file 40064_2012_198_MOESM2_ESM.zip › add2/1118854145799791_fig3.pdf]

Toad #4, Huaico, Salta

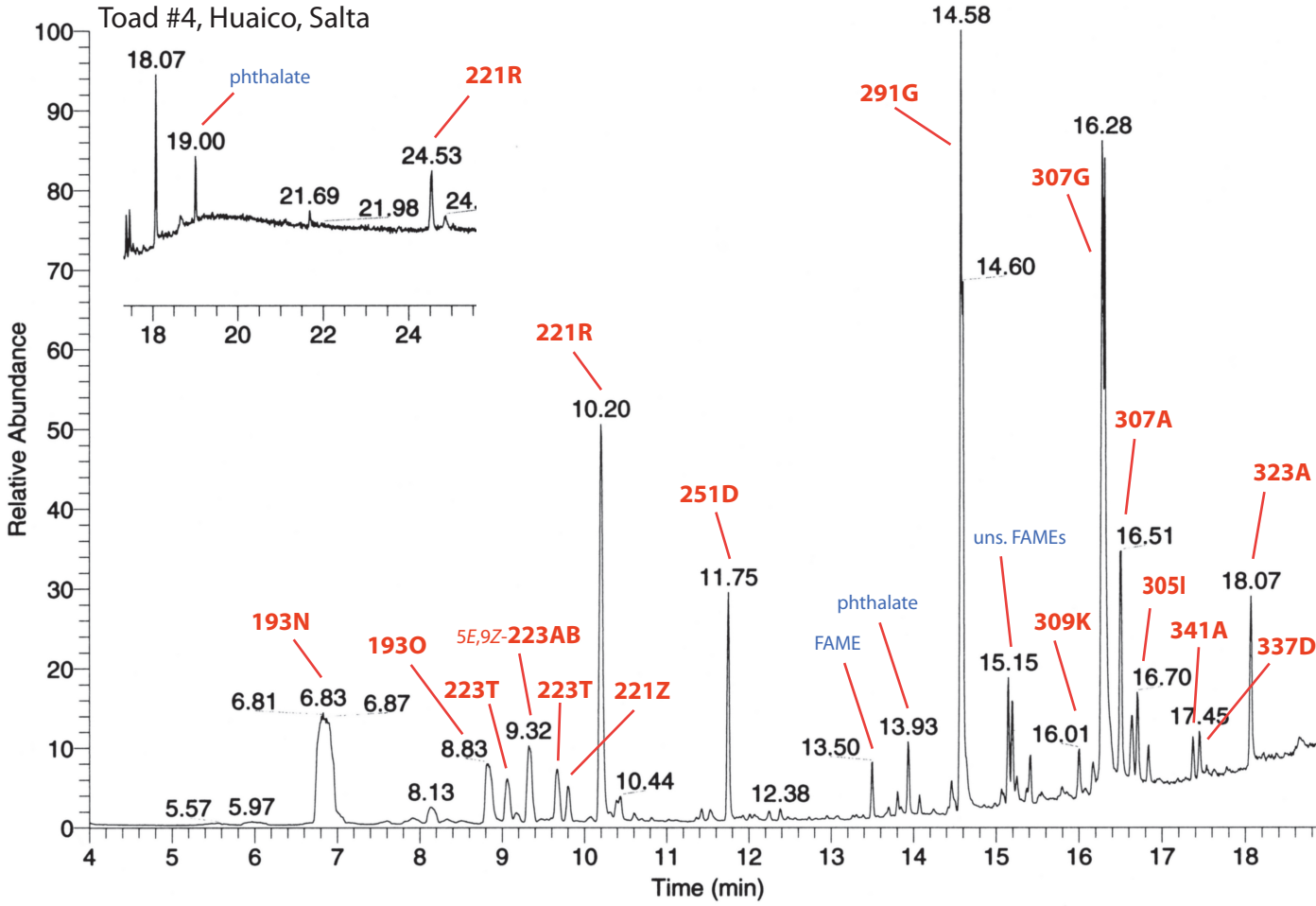

Supplement: Supplementary file 2 — Additional file 2 Figure S27.: Typical ponds where collections were made: a) Cedral de Baritú, Salta; b) Los Paños, Jujuy. The blue parallelogram indicates the average collection area. (ZIP 9 MB) [file 40064_2012_198_MOESM2_ESM.zip › add2/1118854145799791_fig4.pdf]

Toad #5, Los Paños, Jujuy

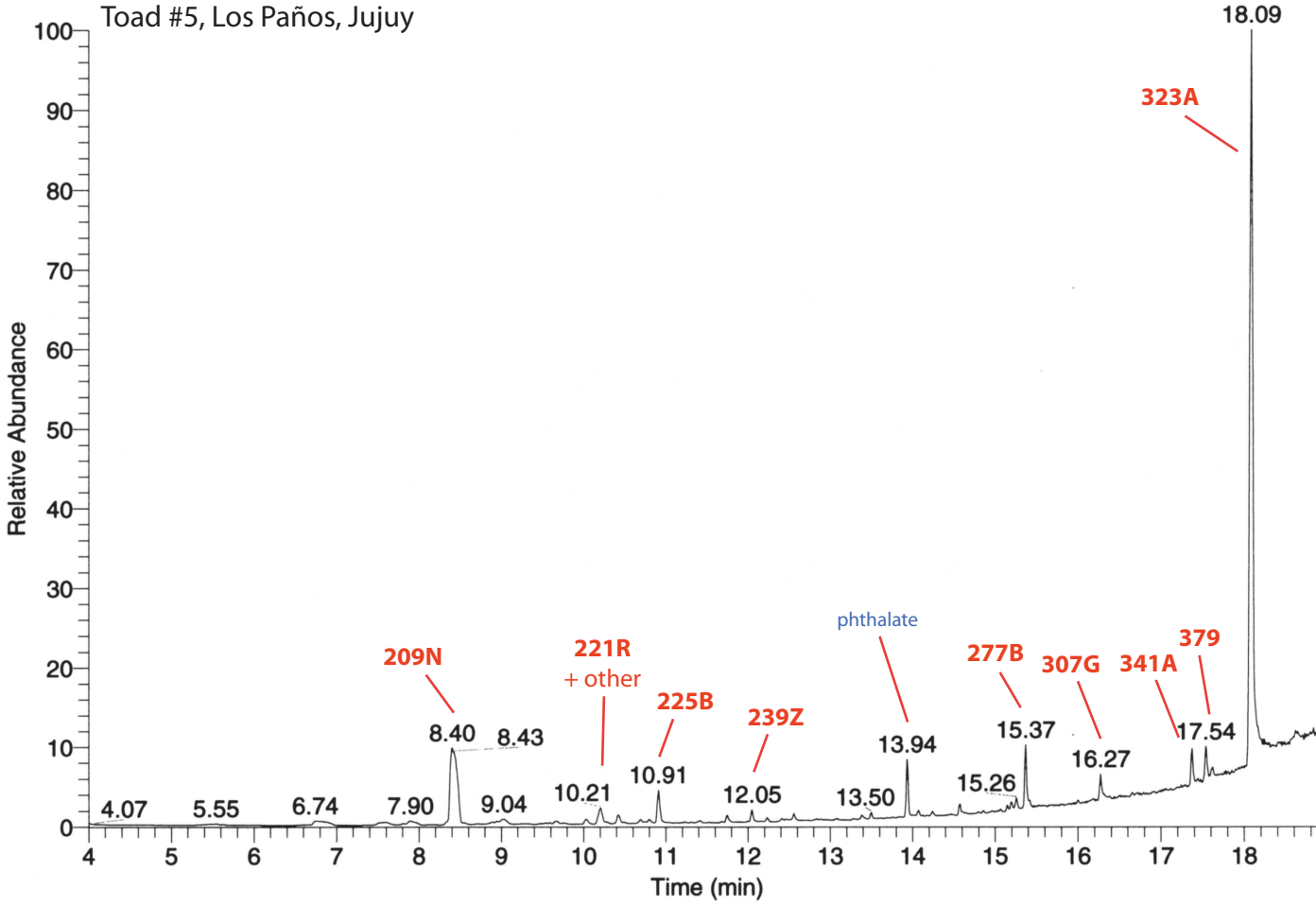

Supplement: Supplementary file 2 — Additional file 2 Figure S27.: Typical ponds where collections were made: a) Cedral de Baritú, Salta; b) Los Paños, Jujuy. The blue parallelogram indicates the average collection area. (ZIP 9 MB) [file 40064_2012_198_MOESM2_ESM.zip › add2/1118854145799791_fig5.pdf]

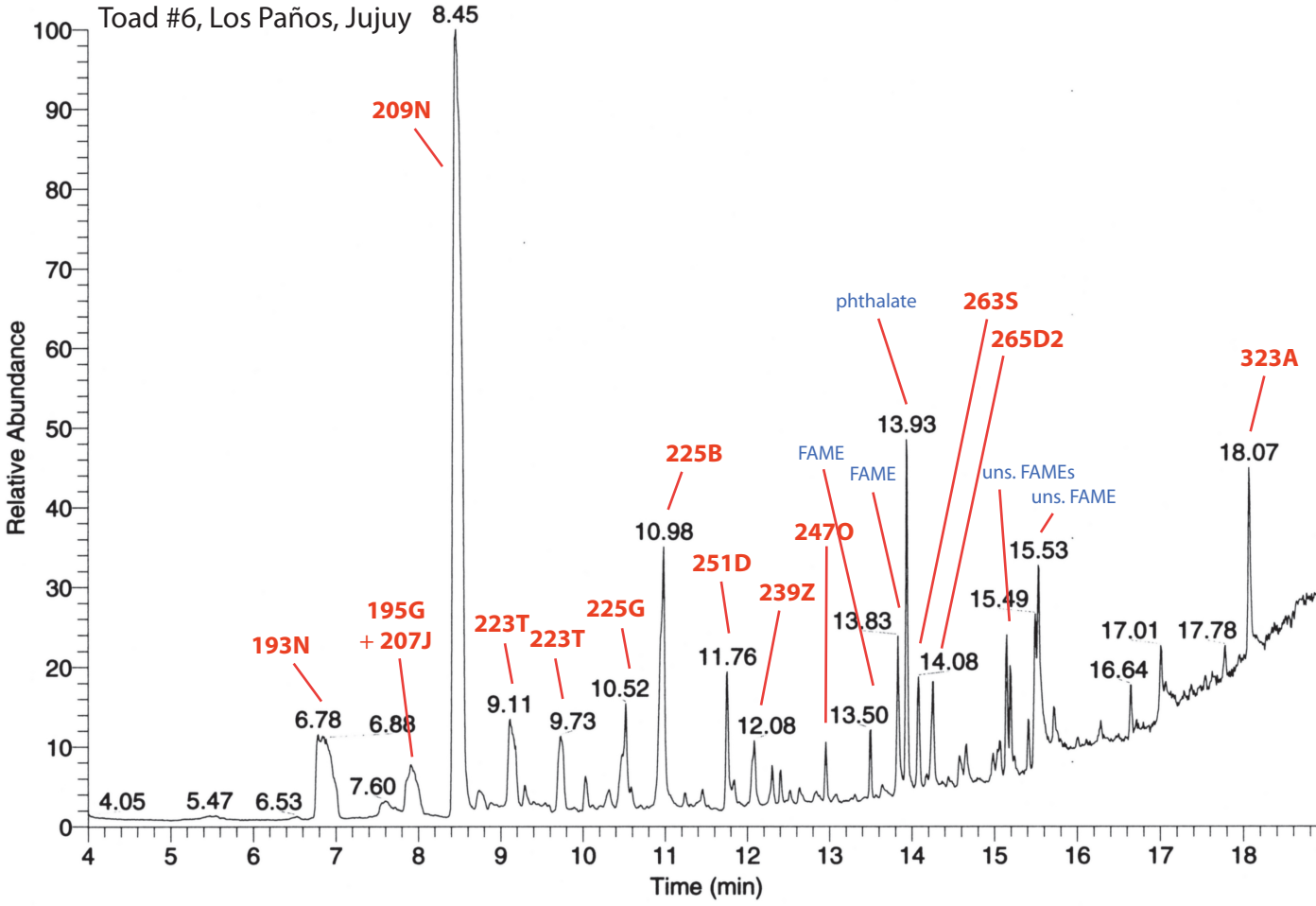

Supplement: Supplementary file 2 — Additional file 2 Figure S27.: Typical ponds where collections were made: a) Cedral de Baritú, Salta; b) Los Paños, Jujuy. The blue parallelogram indicates the average collection area. (ZIP 9 MB) [file 40064_2012_198_MOESM2_ESM.zip › add2/1118854145799791_fig6.pdf]

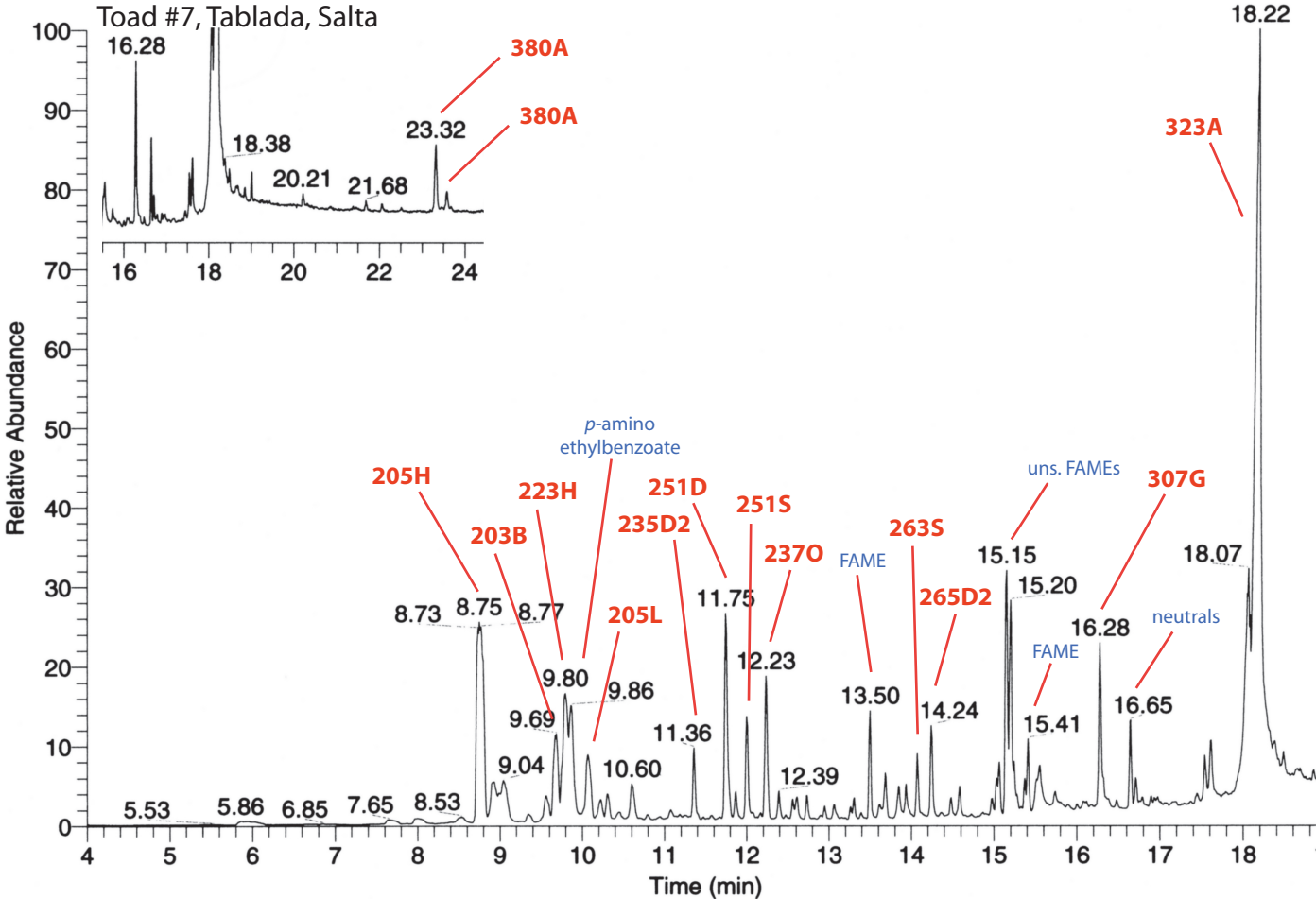

Supplement: Supplementary file 2 — Additional file 2 Figure S27.: Typical ponds where collections were made: a) Cedral de Baritú, Salta; b) Los Paños, Jujuy. The blue parallelogram indicates the average collection area. (ZIP 9 MB) [file 40064_2012_198_MOESM2_ESM.zip › add2/1118854145799791_fig7.pdf]

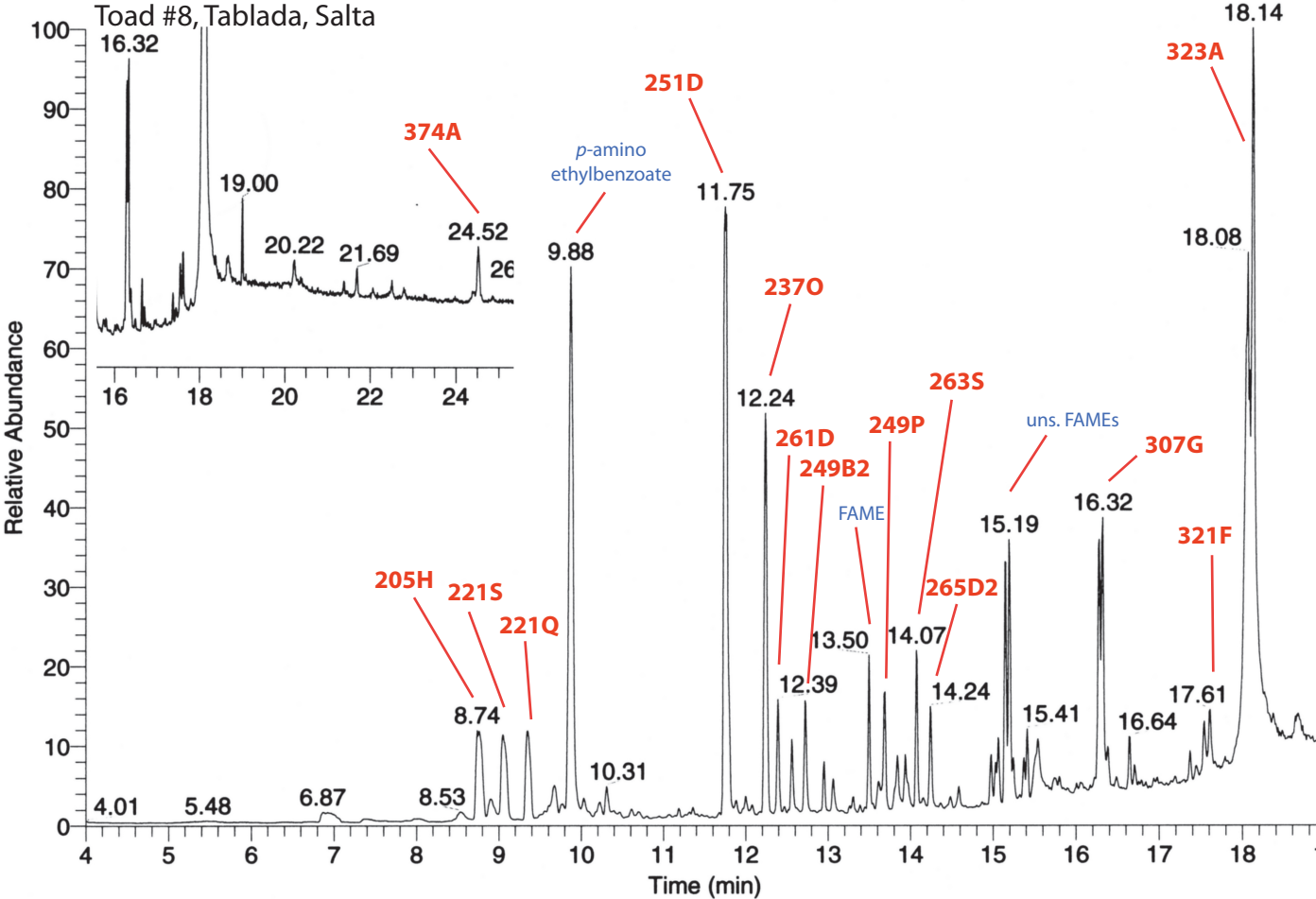

Supplement: Supplementary file 2 — Additional file 2 Figure S27.: Typical ponds where collections were made: a) Cedral de Baritú, Salta; b) Los Paños, Jujuy. The blue parallelogram indicates the average collection area. (ZIP 9 MB) [file 40064_2012_198_MOESM2_ESM.zip › add2/1118854145799791_fig8.pdf]

Toad #9, Cucho, Jujuy

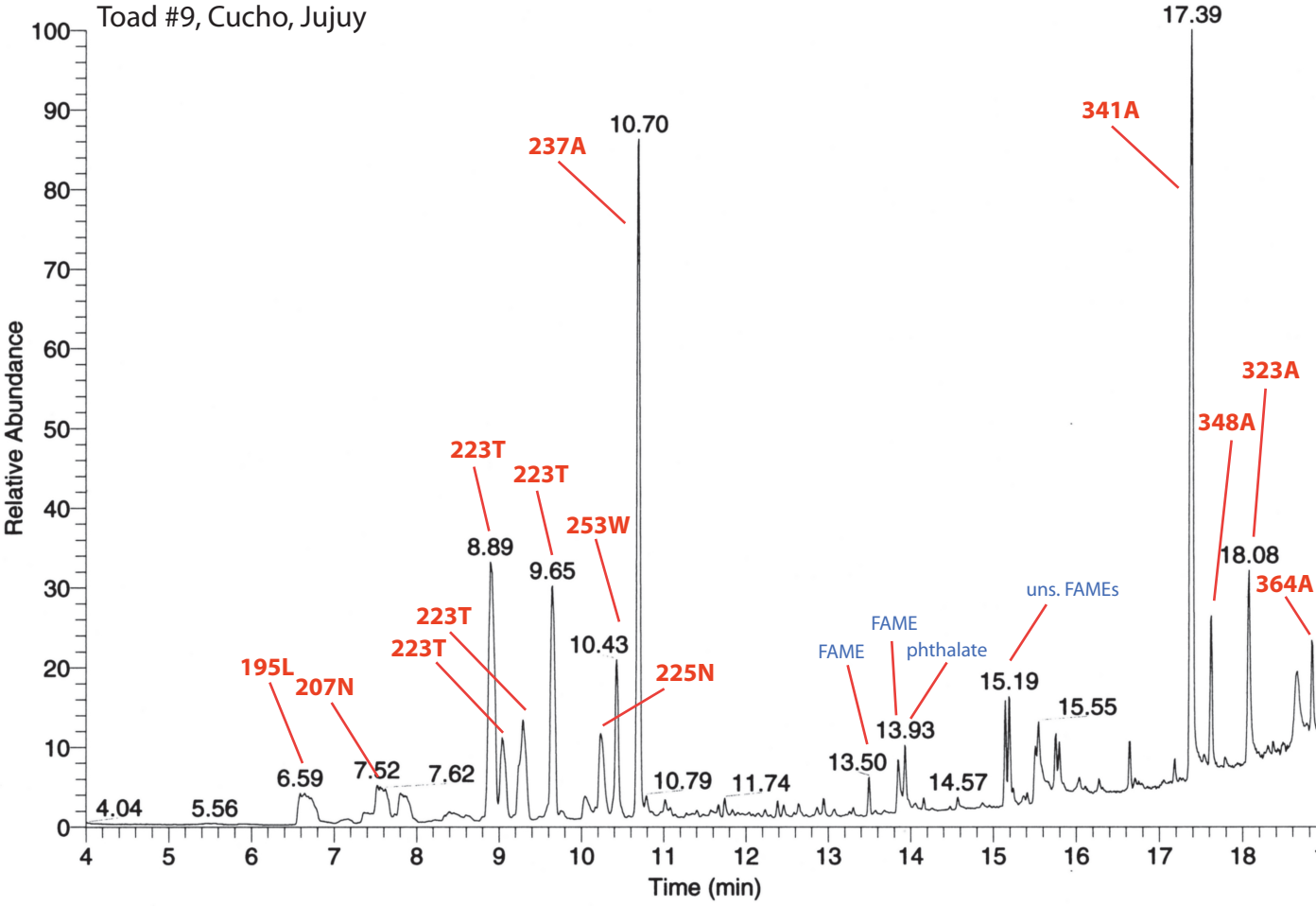

Supplement: Supplementary file 2 — Additional file 2 Figure S27.: Typical ponds where collections were made: a) Cedral de Baritú, Salta; b) Los Paños, Jujuy. The blue parallelogram indicates the average collection area. (ZIP 9 MB) [file 40064_2012_198_MOESM2_ESM.zip › add2/1118854145799791_fig9.pdf]

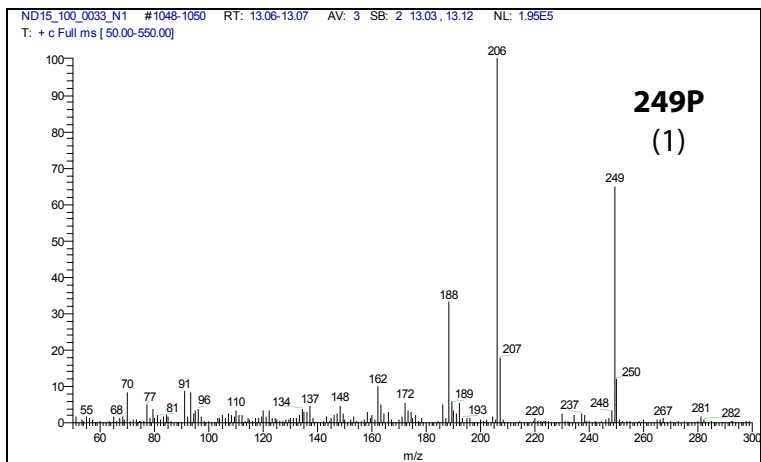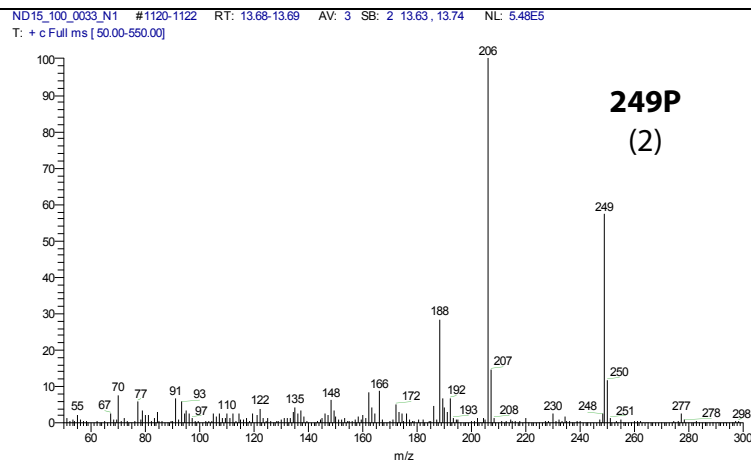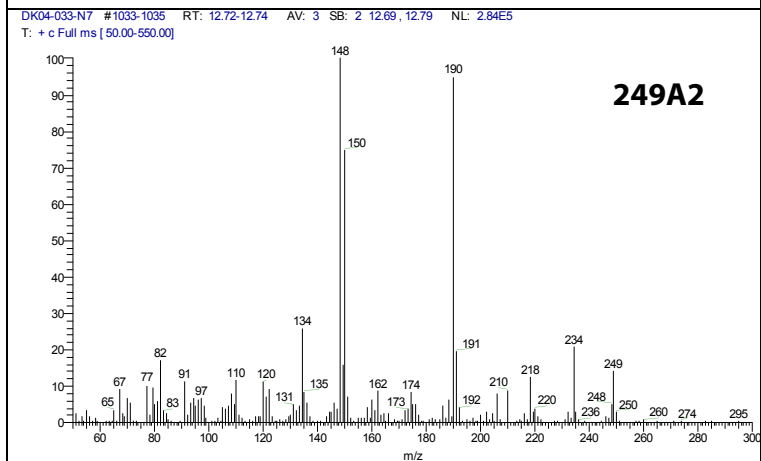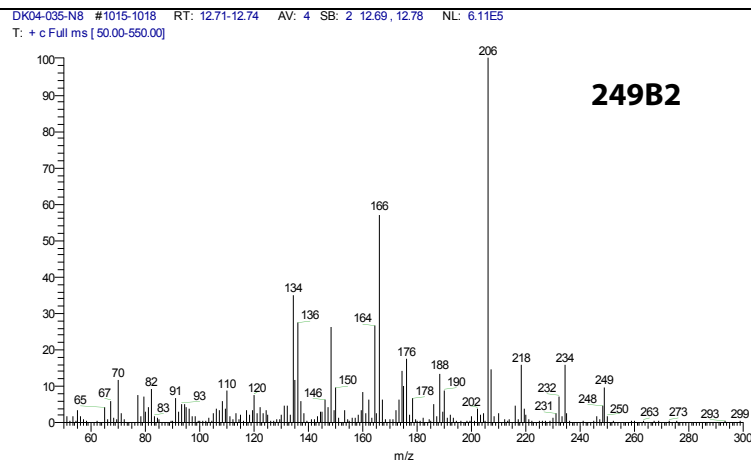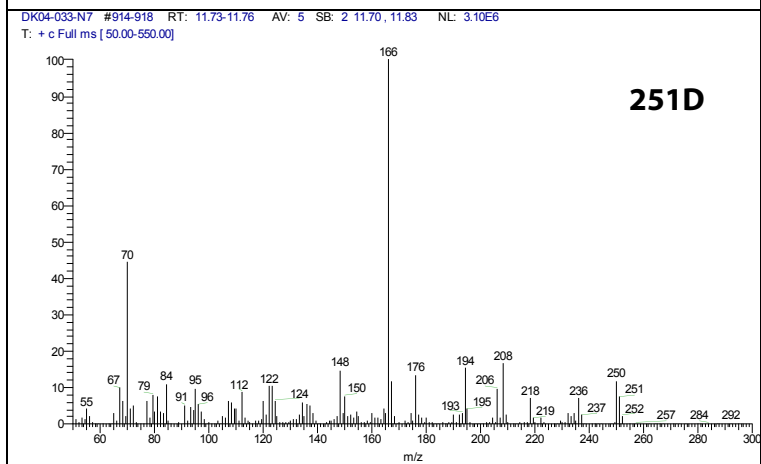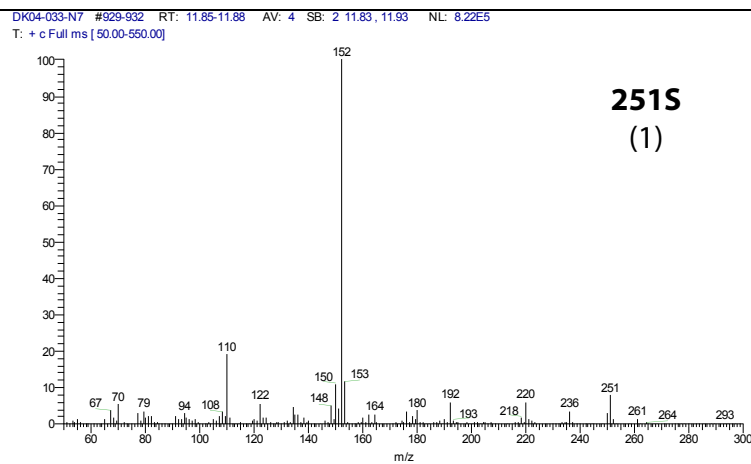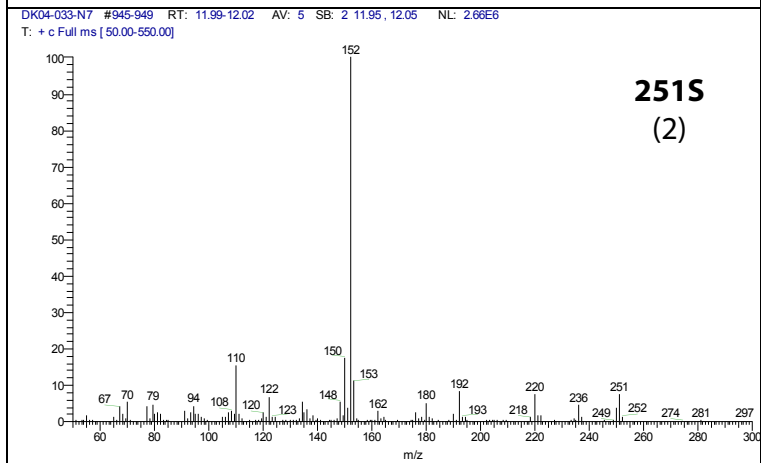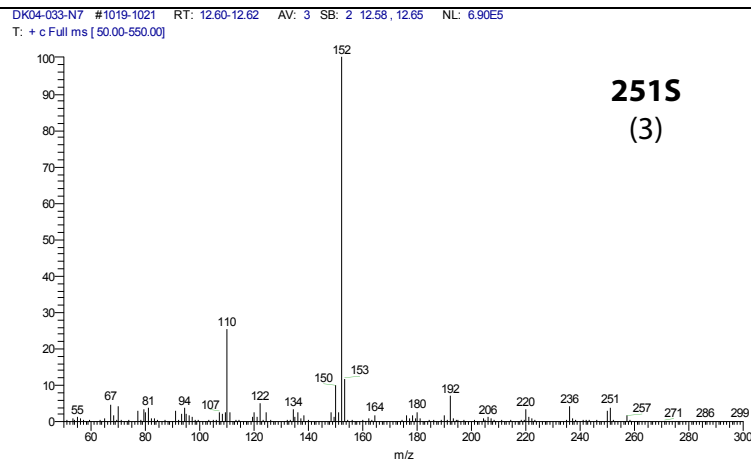

Supplement: Supplementary file 4 — Additional fle 3 Figures S1-S10.: Total mass spectral ion current chromatograms for the alkaloid extracts of toad skin samples #1-10. (ZIP 12984 kb) (ZIP 9566 kb) (ZIP 13 MB) [file 40064_2012_198_MOESM4_ESM.zip › add3/1118854145799791_fig20.pdf]

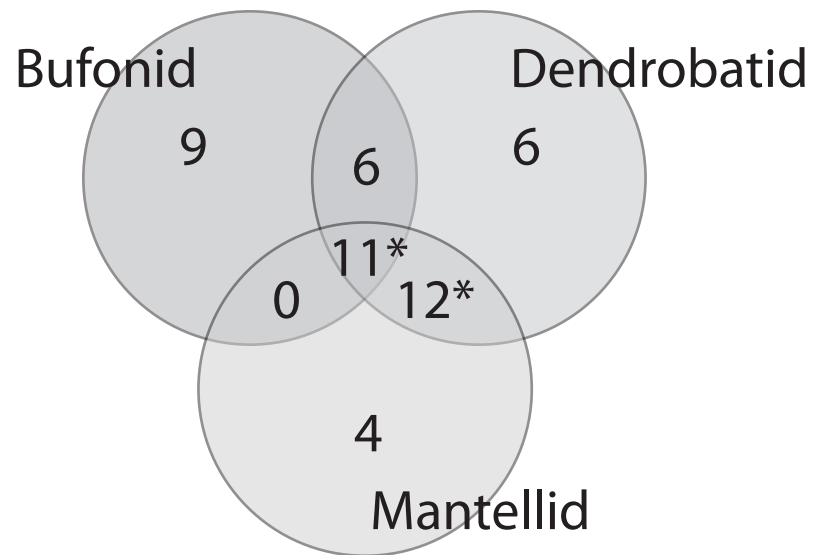

Supplement: Supplementary file 5 — Additional file 5 Figure S28.: Venn diagram showing occurrence and overlaps of previously known anuran skin alkaloids (N = 48) detected in the Argentinian toad Melanophryniscus rubriventris of this study with those previously seen in bufonid toads, dendrobatid or mantellid frogs. See Supplemental Information of Daly et al. 2005 for alkaloids and sources. The asterisk in the dendrobatid/mantellid alkaloid overlap indicates two occurrences also in myobatrachid frogs of Australia. (ZIP 177 KB) [file 40064_2012_198_MOESM5_ESM.zip › add5/1118854145799791_fig28.pdf]
