# Supplementary material for: Alkaloids from single skins of the Argentinian toad Melanophryniscus rubriventris (ANURA, BUFONIDAE): An unexpected variability in alkaloid profiles and a profusion of new structures
Source: Springerplus. 2012 Nov 23;1(1):51. doi: 10.1186/2193-1801-1-51 (PMC3625416; doi:10.1186/2193-1801-1-51)
Supplement: Supplementary file 4 — Additional fle 3 Figures S1-S10.: Total mass spectral ion current chromatograms for the alkaloid extracts of toad skin samples #1-10. (ZIP 12984 kb) (ZIP 9566 kb) (ZIP 13 MB) [file 40064_2012_198_MOESM4_ESM.zip › add3/1118854145799791_fig11.pdf]

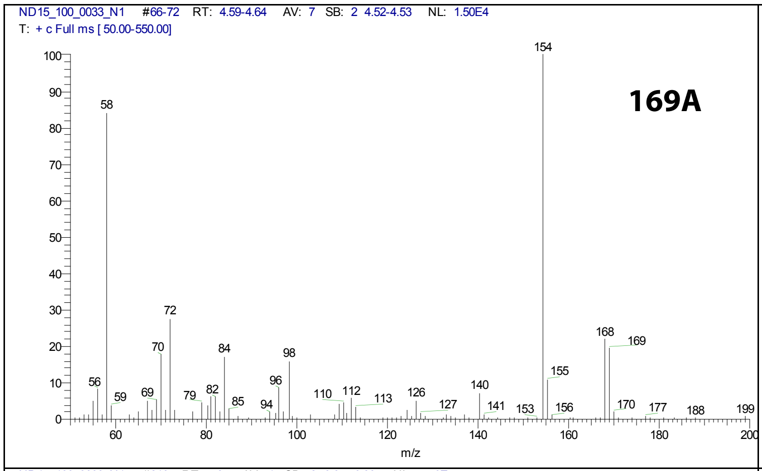
ND15_100_0033_N1 #66-72 RT: 4.59-4.64 AV: 7 SB: 2 4.52-4.53 NL: 1.50E4
T: + c Full ms [50.00-550.00]
169A
m/z

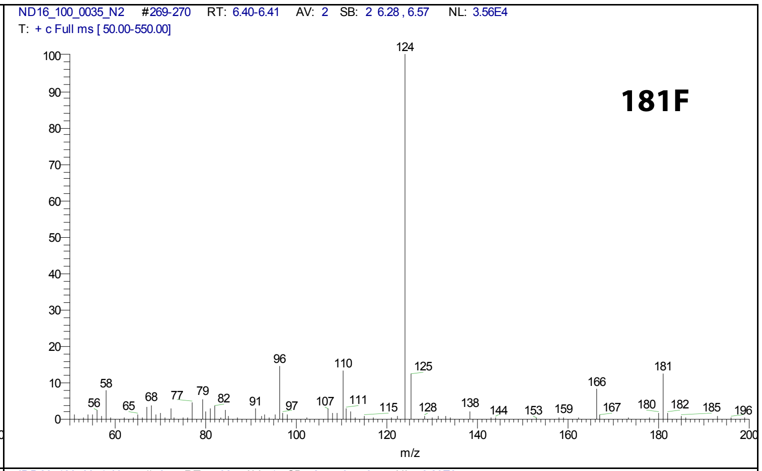
ND16_100_0035_N2 #269-270 RT: 6.40-6.41 AV: 2 SB: 2 6.28 , 6.57 NL: 3.56E4
T: + c Full ms [50.00-550.00]
181F
m/z

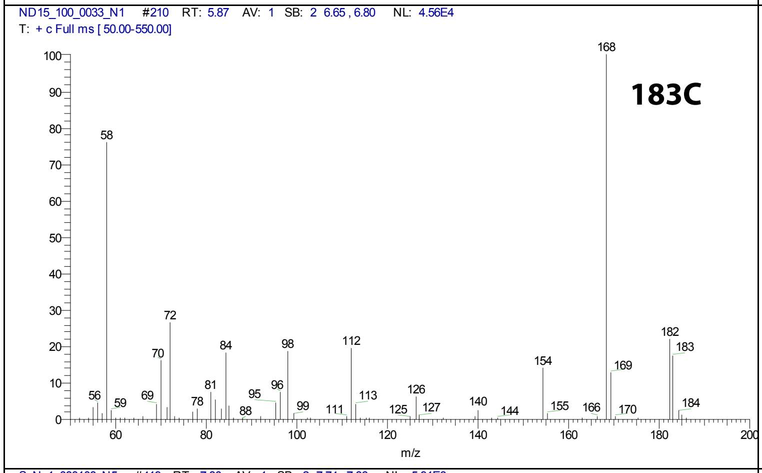
ND15_100_0033_N1 #210 RT: 5.87 AV: 1 SB: 2 6.65 , 6.80 NL: 4.56E4
T: + c Full ms [50.00-550.00]
183C
m/z

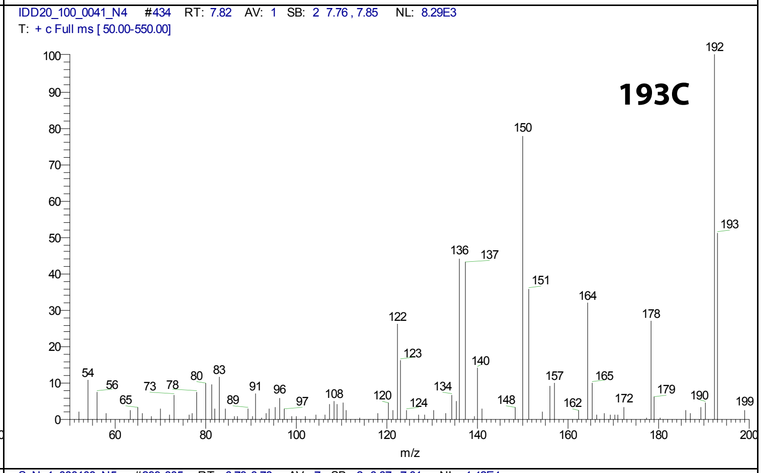
IDD20_100_0041_N4 #434 RT: 7.82 AV: 1 SB: 2 7.76 , 7.85 NL: 8.29E3
T: + c Full ms [50.00-550.00]
193C
m/z

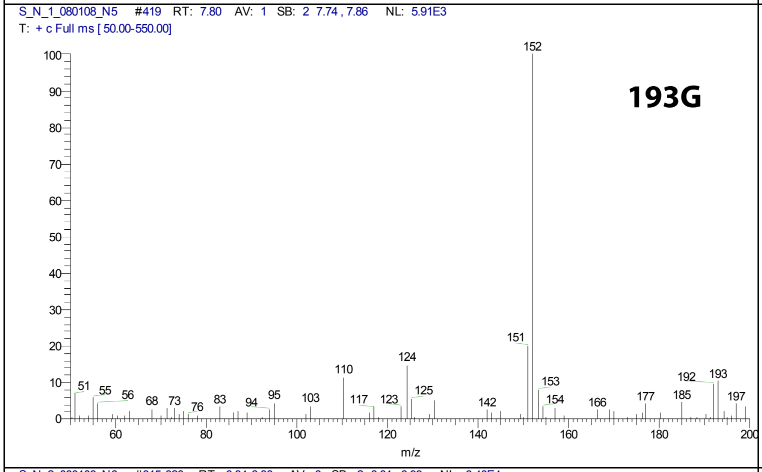
S_N_1_080108_N5 #419 RT: 7.80 AV: 1 SB: 2 7.74 , 7.86 NL: 5.91E3
T: + c Full ms [50.00-550.00]
193G
m/z

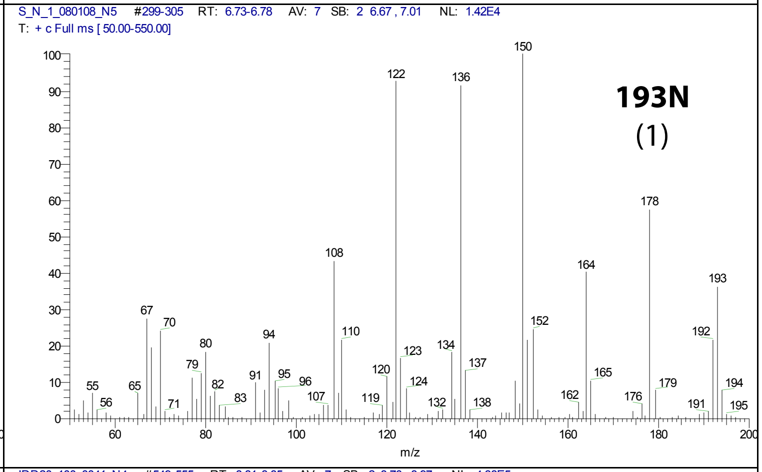
S_N_1_080108_N5 #299-305 RT: 6.73-6.78 AV: 7 SB: 2 6.67 , 7.01 NL: 1.42E4
T: + c Full ms [50.00-550.00]
193N
(1)
m/z

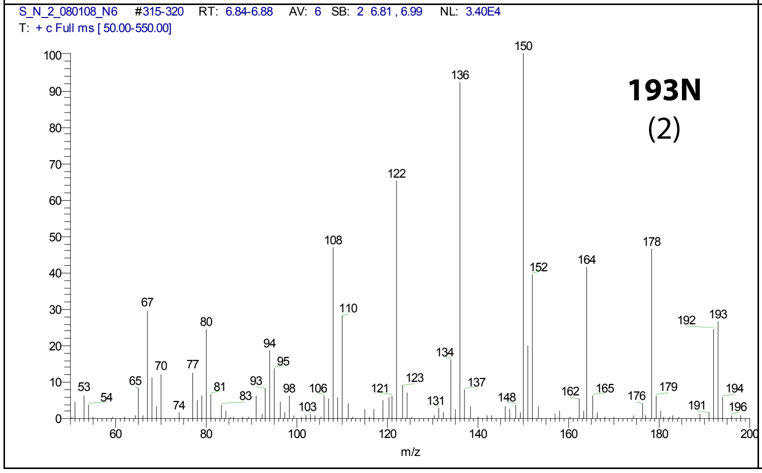
S_N_2_080108_N6 #315-320 RT: 6.84-6.88 AV: 6 SB: 2 6.81 , 6.99 NL: 3.40E4
T: + c Full ms [50.00-550.00]
193N
(2)
m/z

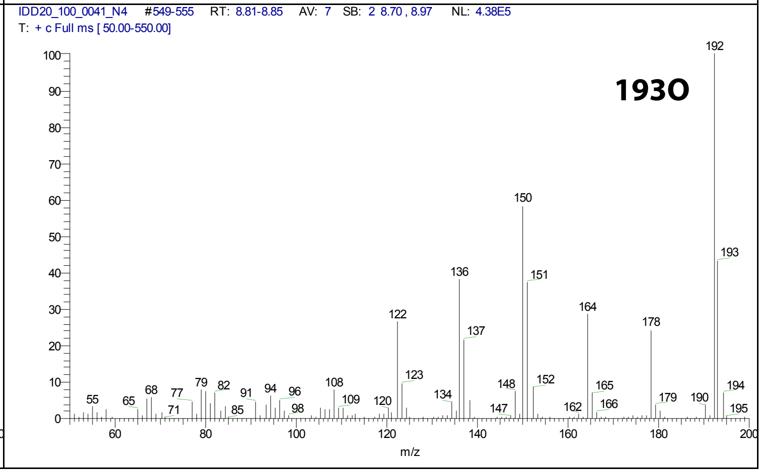
IDD20_100_0041_N4 #549-555 RT: 8.81-8.85 AV: 7 SB: 2 8.70 , 8.97 NL: 4.38E5
T: + c Full ms [50.00-550.00]
193O
m/z
