# Supplementary material for: Alkaloids from single skins of the Argentinian toad Melanophryniscus rubriventris (ANURA, BUFONIDAE): An unexpected variability in alkaloid profiles and a profusion of new structures
Source: Springerplus. 2012 Nov 23;1(1):51. doi: 10.1186/2193-1801-1-51 (PMC3625416; doi:10.1186/2193-1801-1-51)
Supplement: Supplementary file 4 — Additional fle 3 Figures S1-S10.: Total mass spectral ion current chromatograms for the alkaloid extracts of toad skin samples #1-10. (ZIP 12984 kb) (ZIP 9566 kb) (ZIP 13 MB) [file 40064_2012_198_MOESM4_ESM.zip › add3/1118854145799791_fig12.pdf]

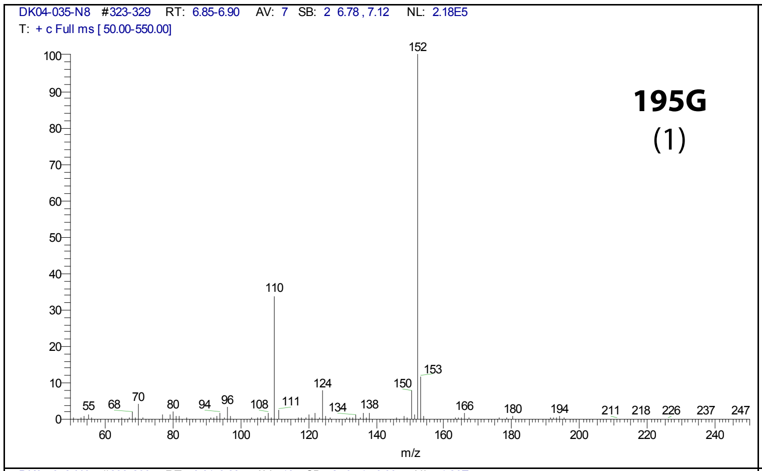
DK04-035-N8 #323-329 RT: 6.85-6.90 AV: 7 SB: 2 6.78 , 7.12 NL: 2.18E5
T: + c Full ms [50.00-550.00]
195G
(1)
152
110
124
150
153
55 68 70 80 94 96 108 111 134 138 166 180 194 211 218 226 237 247
m/z

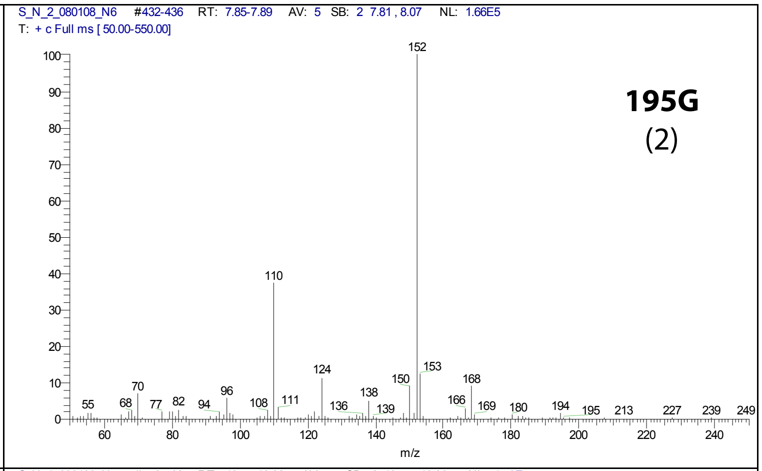
S_N_2_080108_N6 #432-436 RT: 7.85-7.89 AV: 5 SB: 2 7.81 , 8.07 NL: 1.66E5
T: + c Full ms [50.00-550.00]
195G
(2)
152
110
124
150 153
168
55 68 70 77 82 94 96 108 111 136 138 139 166 169 180 194 195 213 227 239 249
m/z

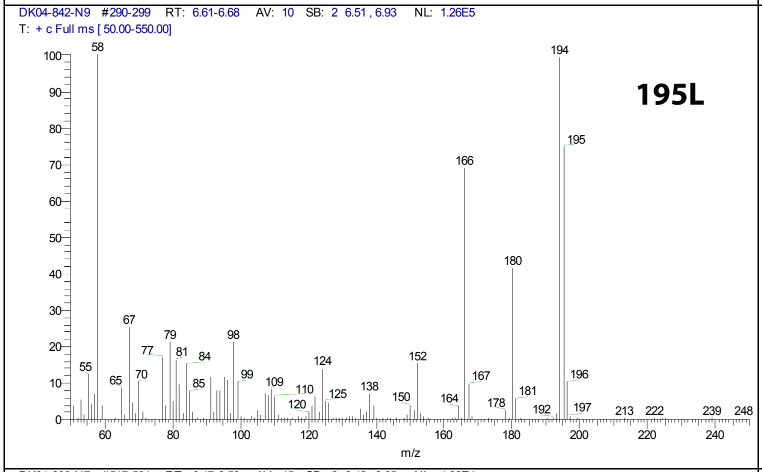
DK04-842-N9 #290-299 RT: 6.61-6.68 AV: 10 SB: 2 6.51 , 6.93 NL: 1.26E5
T: + c Full ms [50.00-550.00]
195L
58
194
195
166
180
67 79 98
77 81 84 124 152
55 65 70 85 99 109 110 120 125 138 150 164 167 178 181 192 196 197 213 222 239 248
m/z

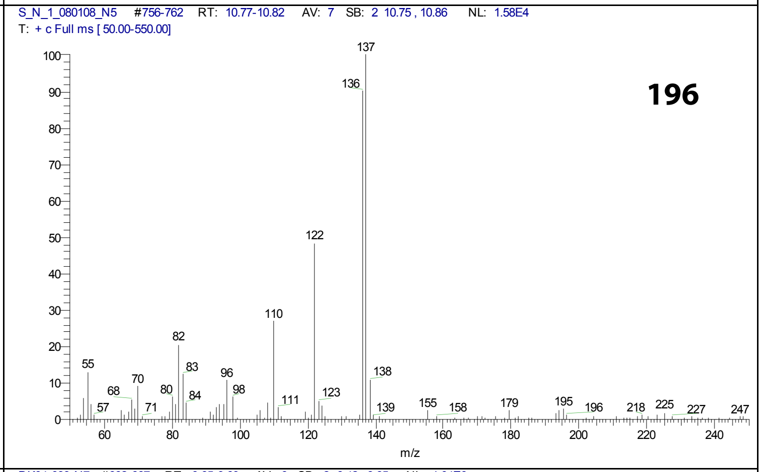
S_N_1_080108_N5 #756-762 RT: 10.77-10.82 AV: 7 SB: 2 10.75 , 10.86 NL: 1.58E4
T: + c Full ms [50.00-550.00]
196
137
136
122
110
82
55 57 68 70 71 80 83 84 96 98 111 123 138 139 155 158 179 195 196 218 225 227 247
m/z

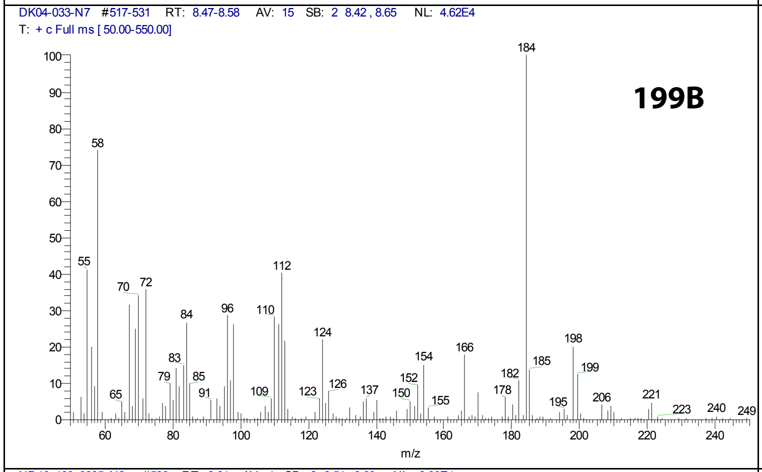
DK04-033-N7 #517-531 RT: 8.47-8.58 AV: 15 SB: 2 8.42 , 8.65 NL: 4.62E4
T: + c Full ms [50.00-550.00]
199B
184
58
55
112
70 72
84 96 110
124
166 198
83 154 185
79 85 152 182 199
65 91 109 123 126 137 150 155 178 195 206 221 223 240 249
m/z

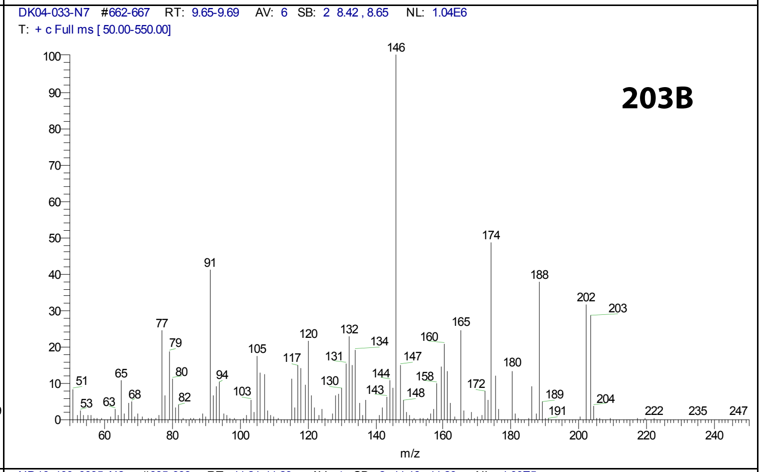
DK04-033-N7 #662-667 RT: 9.65-9.69 AV: 6 SB: 2 8.42 , 8.65 NL: 1.04E6
T: + c Full ms [50.00-550.00]
203B
146
174
91
188
202 203
77 132 165
79 120 134 160
105 117 131 147 180
51 80 94 144 158 172
53 63 65 68 82 103 130 143 148 189 191 204 222 235 247
m/z

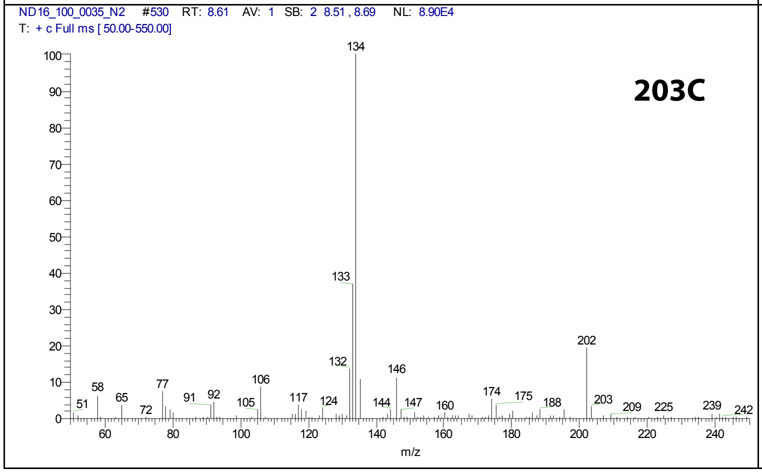
ND16_100_0035_N2 #530 RT: 8.61 AV: 1 SB: 2 8.51 , 8.69 NL: 8.90E4
T: + c Full ms [50.00-550.00]
203C
134
133
202
132 146
106 174 175
51 58 65 72 77 91 92 105 117 124 144 147 160 188 203 209 225 239 242
m/z

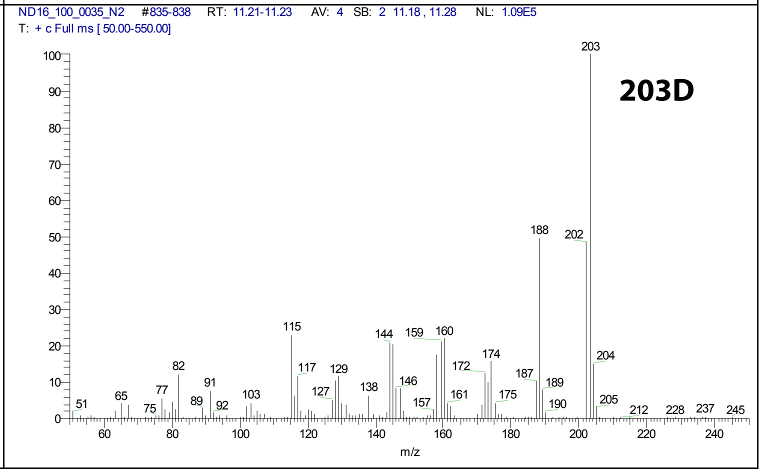
ND16_100_0035_N2 #835-838 RT: 11.21-11.23 AV: 4 SB: 2 11.18 , 11.28 NL: 1.09E5
T: + c Full ms [50.00-550.00]
203D
203
188 202
115
144 159 160
82 117 129 146 172 174 187 189 204
51 65 75 77 89 91 92 103 127 138 157 161 175 190 205 212 228 237 245
m/z
