# Supplementary material for: Alkaloids from single skins of the Argentinian toad Melanophryniscus rubriventris (ANURA, BUFONIDAE): An unexpected variability in alkaloid profiles and a profusion of new structures
Source: Springerplus. 2012 Nov 23;1(1):51. doi: 10.1186/2193-1801-1-51 (PMC3625416; doi:10.1186/2193-1801-1-51)
Supplement: Supplementary file 4 — Additional fle 3 Figures S1-S10.: Total mass spectral ion current chromatograms for the alkaloid extracts of toad skin samples #1-10. (ZIP 12984 kb) (ZIP 9566 kb) (ZIP 13 MB) [file 40064_2012_198_MOESM4_ESM.zip › add3/1118854145799791_fig13.pdf]

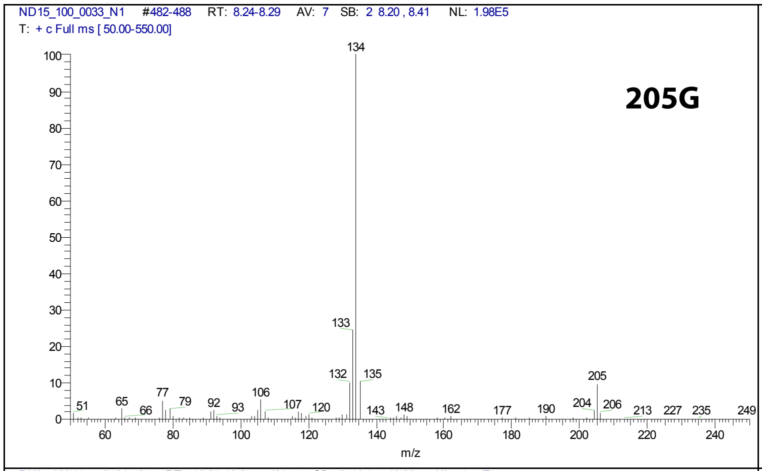
ND15_100_0033_N1 #482-488 RT: 8.24-8.29 AV: 7 SB: 2 8.20 , 8.41 NL: 1.98E5
T: + c Full ms [50.00-550.00]
205G
51 65 66 77 79 92 93 106 107 120 132 133 134 135 143 148 162 177 190 204 205 206 213 227 235 249
m/z

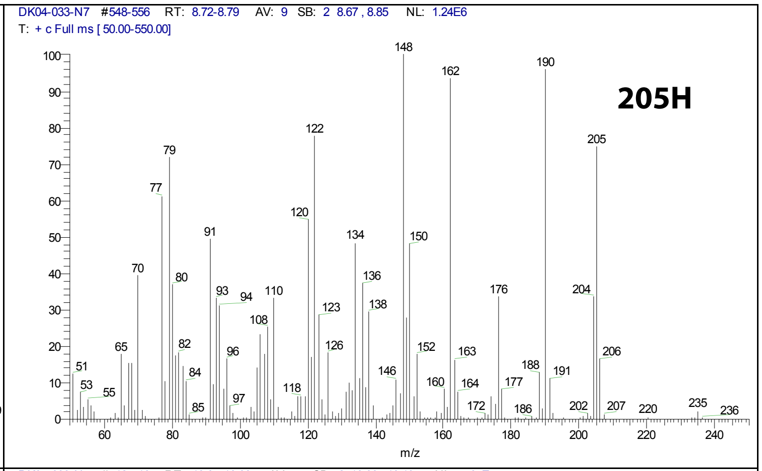
DK04-033-N7 #548-556 RT: 8.72-8.79 AV: 9 SB: 2 8.67 , 8.85 NL: 1.24E6
T: + c Full ms [50.00-550.00]
205H
51 53 55 65 70 77 79 80 82 84 85 91 93 94 96 97 108 110 118 120 122 123 126 134 136 138 146 148 150 152 160 162 163 164 172 176 177 186 188 190 191 202 204 205 206 207 220 235 236
m/z

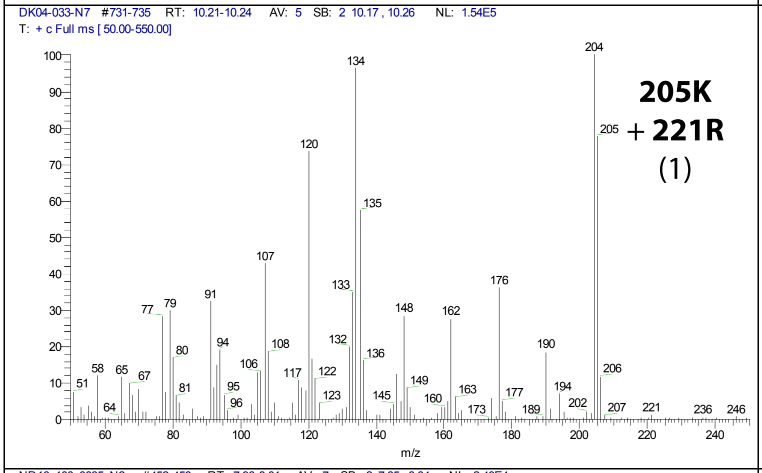
DK04-033-N7 #731-735 RT: 10.21-10.24 AV: 5 SB: 2 10.17 , 10.26 NL: 1.54E5
T: + c Full ms [50.00-550.00]
205K
+ 221R
(1)
51 58 64 65 67 77 79 80 81 91 94 95 96 106 107 108 117 120 122 123 132 133 134 135 136 145 148 149 160 162 163 173 176 177 189 190 194 202 204 205 206 207 221 236 246
m/z

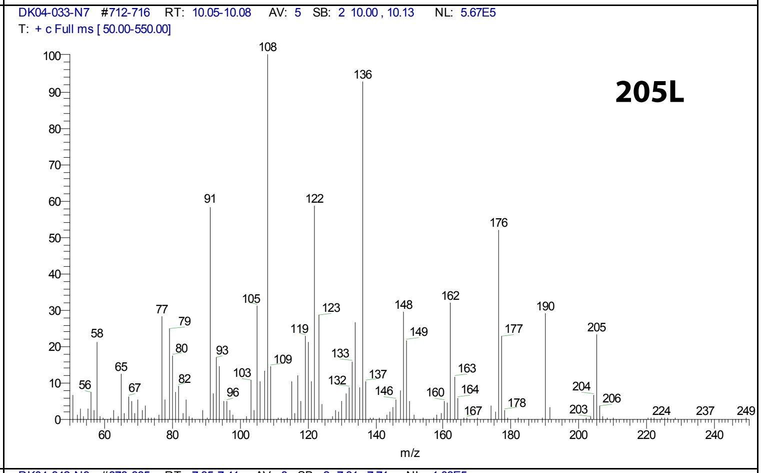
DK04-033-N7 #712-716 RT: 10.05-10.08 AV: 5 SB: 2 10.00 , 10.13 NL: 5.67E5
T: + c Full ms [50.00-550.00]
205L
56 58 65 67 77 79 80 82 91 93 96 103 105 108 109 119 122 123 132 133 136 137 146 148 149 160 162 163 164 167 176 177 178 190 203 204 205 206 224 237 249
m/z

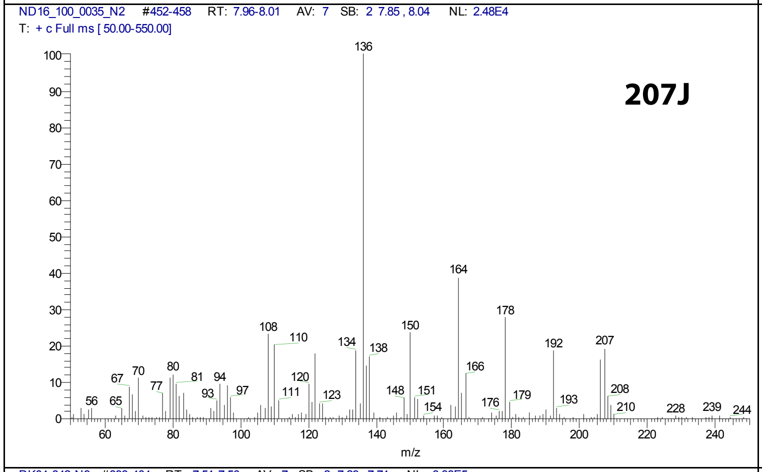
ND16_100_0035_N2 #452-458 RT: 7.96-8.01 AV: 7 SB: 2 7.85 , 8.04 NL: 2.48E4
T: + c Full ms [50.00-550.00]
207J
56 65 67 70 77 80 81 93 94 97 108 110 111 120 123 134 136 138 148 150 151 154 164 166 176 178 179 192 193 207 208 210 228 239 244
m/z

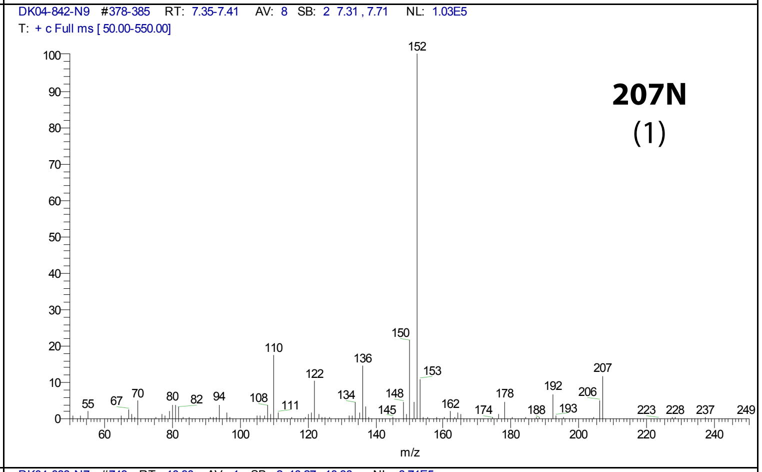
DK04-842-N9 #378-385 RT: 7.35-7.41 AV: 8 SB: 2 7.31 , 7.71 NL: 1.03E5
T: + c Full ms [50.00-550.00]
207N
(1)
55 67 70 80 82 94 108 110 111 122 134 136 145 148 150 152 153 162 174 178 188 192 193 206 207 223 228 237 249
m/z

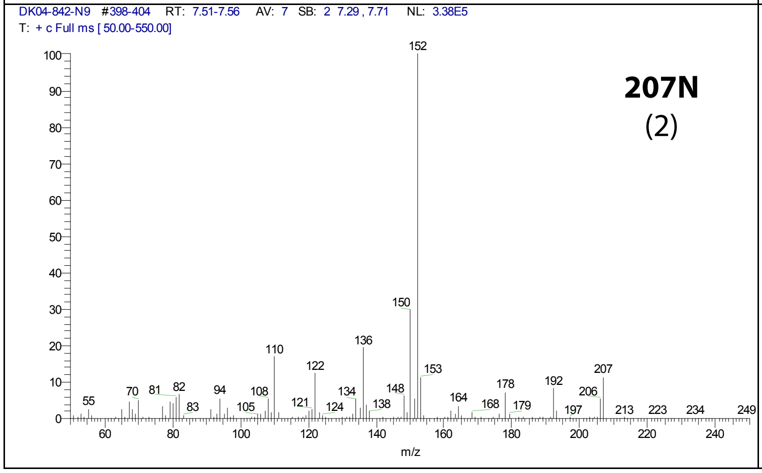
DK04-842-N9 #398-404 RT: 7.51-7.56 AV: 7 SB: 2 7.29 , 7.71 NL: 3.38E5
T: + c Full ms [50.00-550.00]
207N
(2)
55 70 81 82 83 94 105 108 110 121 122 124 134 136 138 148 150 152 153 164 168 178 179 192 197 206 207 213 223 234 249
m/z

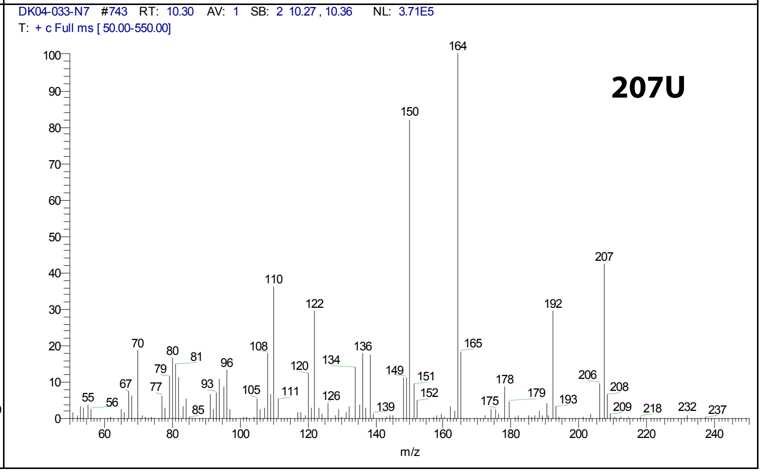
DK04-033-N7 #743 RT: 10.30 AV: 1 SB: 2 10.27 , 10.36 NL: 3.71E5
T: + c Full ms [50.00-550.00]
207U
55 56 67 70 77 79 80 81 85 93 96 105 108 110 111 120 122 126 134 136 139 149 150 151 152 164 165 175 178 179 192 193 206 207 208 209 218 232 237
m/z
