# Supplementary material for: Alkaloids from single skins of the Argentinian toad Melanophryniscus rubriventris (ANURA, BUFONIDAE): An unexpected variability in alkaloid profiles and a profusion of new structures
Source: Springerplus. 2012 Nov 23;1(1):51. doi: 10.1186/2193-1801-1-51 (PMC3625416; doi:10.1186/2193-1801-1-51)
Supplement: Supplementary file 4 — Additional fle 3 Figures S1-S10.: Total mass spectral ion current chromatograms for the alkaloid extracts of toad skin samples #1-10. (ZIP 12984 kb) (ZIP 9566 kb) (ZIP 13 MB) [file 40064_2012_198_MOESM4_ESM.zip › add3/1118854145799791_fig14.pdf]

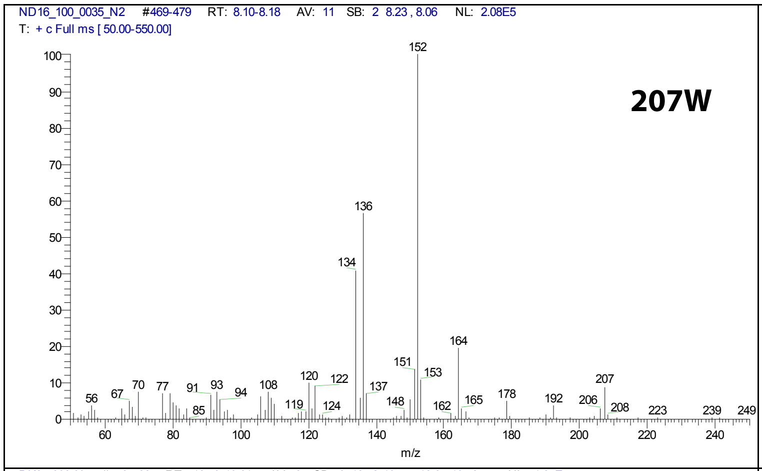
ND16_100_0035_N2 #469-479 RT: 8.10-8.18 AV: 11 SB: 2 8.23 , 8.06 NL: 2.08E5
T: + c Full ms [50.00-550.00]
207W
m/z

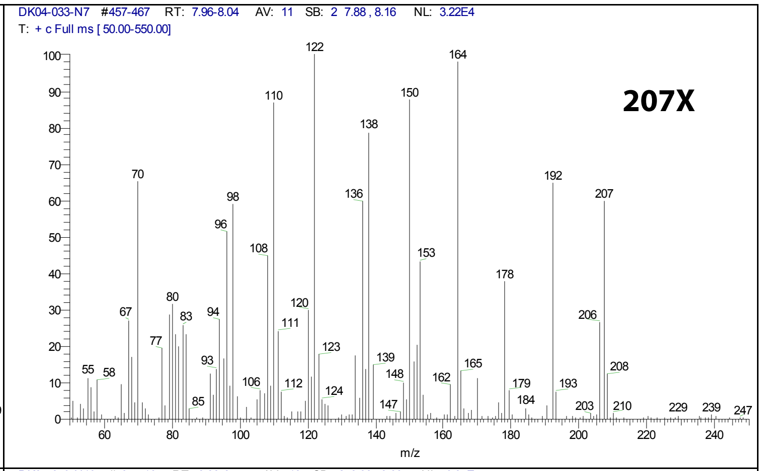
DK04-033-N7 #457-467 RT: 7.96-8.04 AV: 11 SB: 2 7.88 , 8.16 NL: 3.22E4
T: + c Full ms [50.00-550.00]
207X
m/z

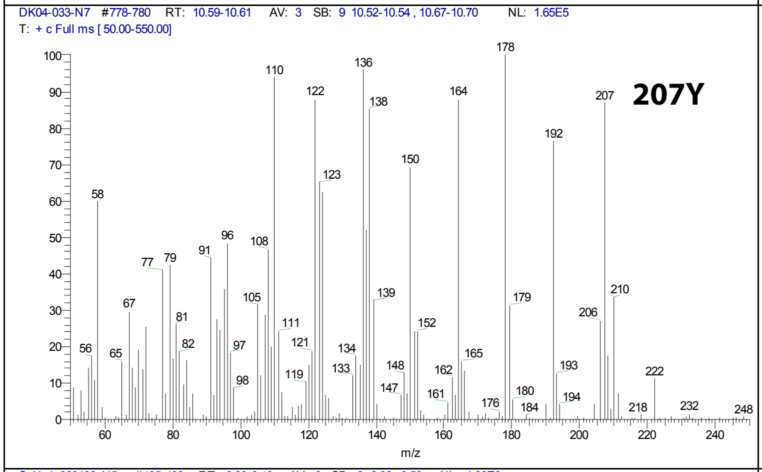
DK04-033-N7 #778-780 RT: 10.59-10.61 AV: 3 SB: 9 10.52-10.54 , 10.67-10.70 NL: 1.65E5
T: + c Full ms [50.00-550.00]
207Y
m/z

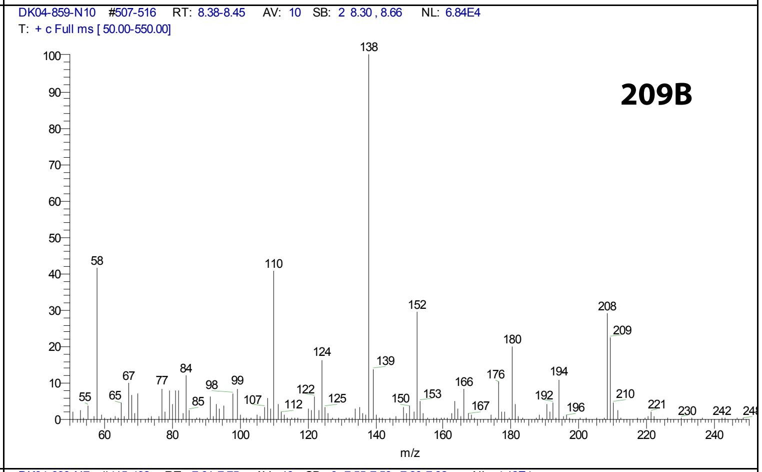
DK04-859-N10 #507-516 RT: 8.38-8.45 AV: 10 SB: 2 8.30 , 8.66 NL: 6.84E4
T: + c Full ms [50.00-550.00]
209B
m/z

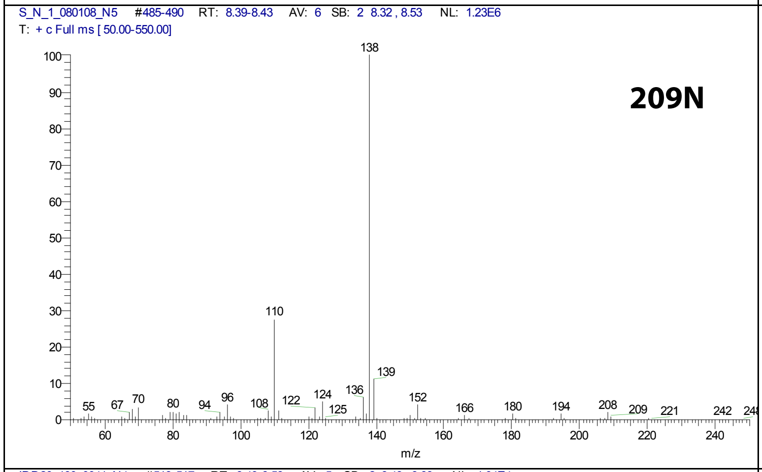
S_N_1_080108_N5 #485-490 RT: 8.39-8.43 AV: 6 SB: 2 8.32 , 8.53 NL: 1.23E6
T: + c Full ms [50.00-550.00]
209N
m/z

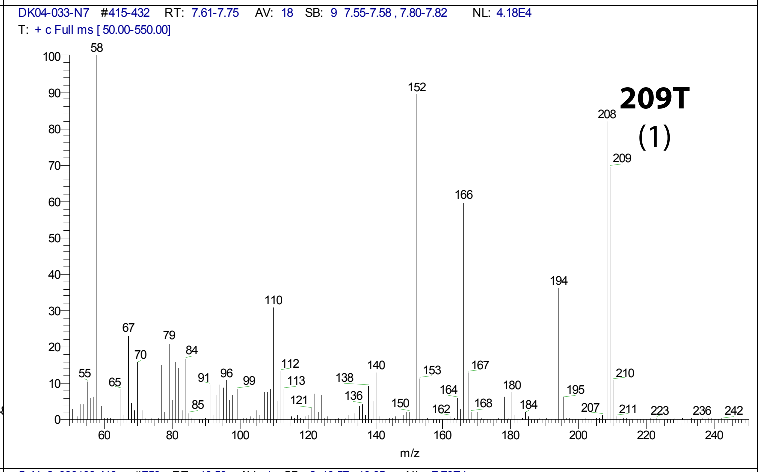
DK04-033-N7 #415-432 RT: 7.61-7.75 AV: 18 SB: 9 7.55-7.58 , 7.80-7.82 NL: 4.18E4
T: + c Full ms [50.00-550.00]
209T
(1)
m/z

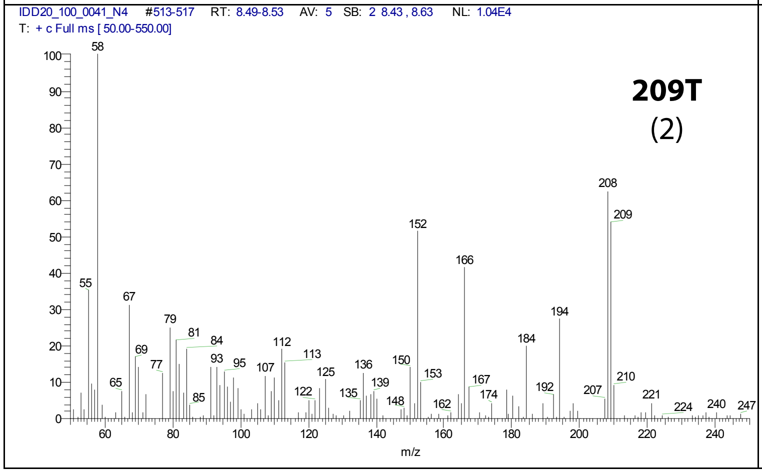
IDD20_100_0041_N4 #513-517 RT: 8.49-8.53 AV: 5 SB: 2 8.43 , 8.63 NL: 1.04E4
T: + c Full ms [50.00-550.00]
209T
(2)
m/z

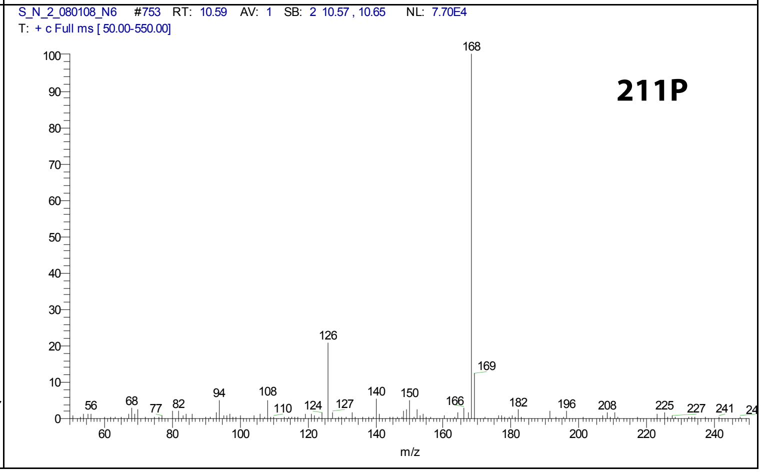
S_N_2_080108_N6 #753 RT: 10.59 AV: 1 SB: 2 10.57 , 10.65 NL: 7.70E4
T: + c Full ms [50.00-550.00]
211P
m/z
