# Supplementary material for: Alkaloids from single skins of the Argentinian toad Melanophryniscus rubriventris (ANURA, BUFONIDAE): An unexpected variability in alkaloid profiles and a profusion of new structures
Source: Springerplus. 2012 Nov 23;1(1):51. doi: 10.1186/2193-1801-1-51 (PMC3625416; doi:10.1186/2193-1801-1-51)
Supplement: Supplementary file 4 — Additional fle 3 Figures S1-S10.: Total mass spectral ion current chromatograms for the alkaloid extracts of toad skin samples #1-10. (ZIP 12984 kb) (ZIP 9566 kb) (ZIP 13 MB) [file 40064_2012_198_MOESM4_ESM.zip › add3/1118854145799791_fig15.pdf]

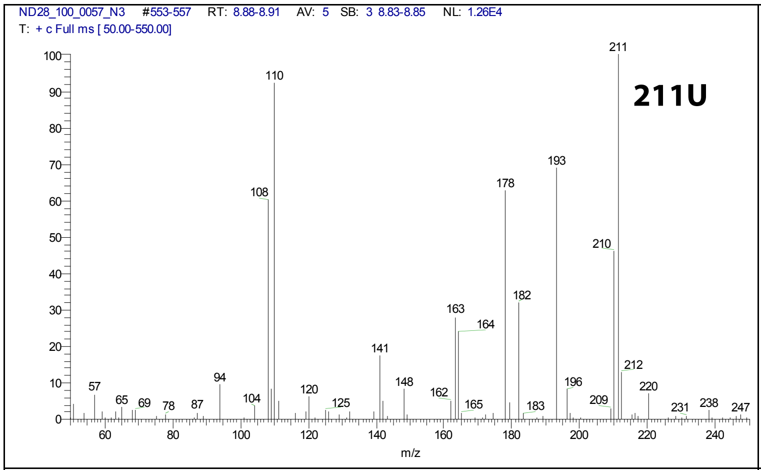
ND28_100_0057_N3 #553-557 RT: 8.88-8.91 AV: 5 SB: 3 8.83-8.85 NL: 1.26E4
T: + c Full ms [50.00-550.00]
211U
57 65 69 78 87 94 104 108 110 120 125 141 148 162 163 164 165 178 182 183 193 196 209 210 211 212 220 231 238 247
m/z

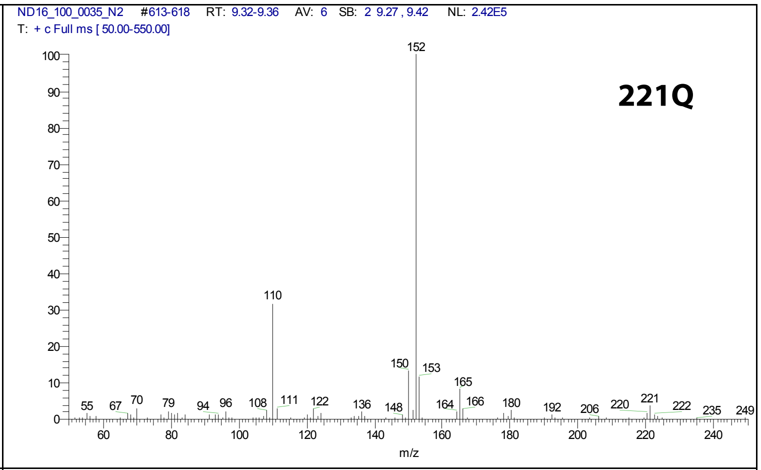
ND16_100_0035_N2 #613-618 RT: 9.32-9.36 AV: 6 SB: 2 9.27 , 9.42 NL: 2.42E5
T: + c Full ms [50.00-550.00]
221Q
55 67 70 79 94 96 108 110 111 122 136 148 150 152 153 164 165 166 180 192 206 220 221 222 235 249
m/z

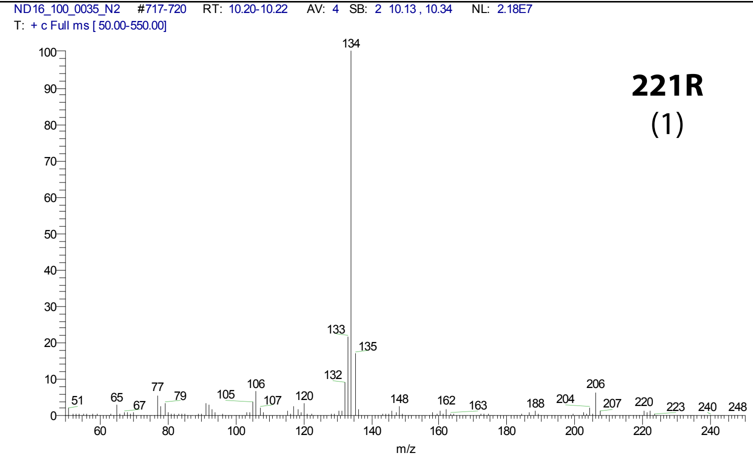
ND16_100_0035_N2 #717-720 RT: 10.20-10.22 AV: 4 SB: 2 10.13 , 10.34 NL: 2.18E7
T: + c Full ms [50.00-550.00]
221R
(1)
51 65 67 77 79 105 106 107 120 132 133 134 135 148 162 163 188 204 206 207 220 223 240 248
m/z

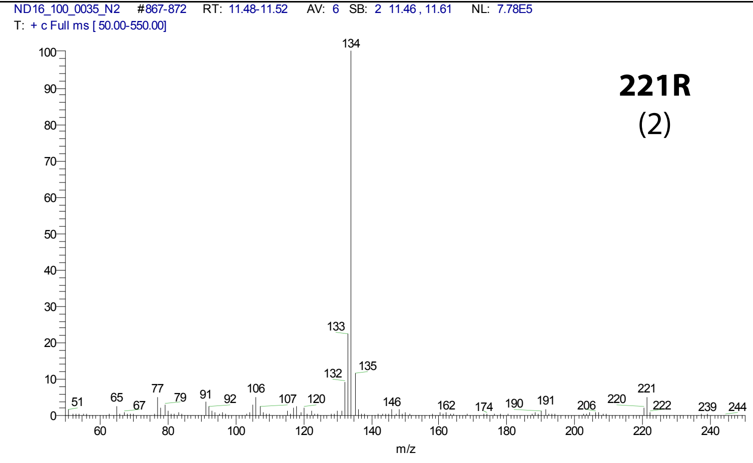
ND16_100_0035_N2 #867-872 RT: 11.48-11.52 AV: 6 SB: 2 11.46 , 11.61 NL: 7.78E5
T: + c Full ms [50.00-550.00]
221R
(2)
51 65 67 77 79 91 92 106 107 120 132 133 134 135 146 162 174 190 191 206 220 221 222 239 244
m/z

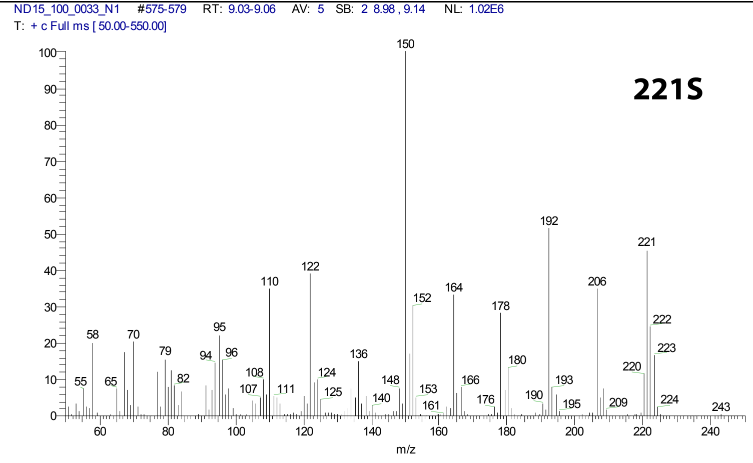
ND15_100_0033_N1 #575-579 RT: 9.03-9.06 AV: 5 SB: 2 8.98 , 9.14 NL: 1.02E6
T: + c Full ms [50.00-550.00]
221S
55 58 65 70 79 82 94 95 96 107 108 110 111 122 124 125 136 140 148 150 152 153 161 164 166 176 178 180 190 192 193 195 206 209 220 221 222 223 224 243
m/z

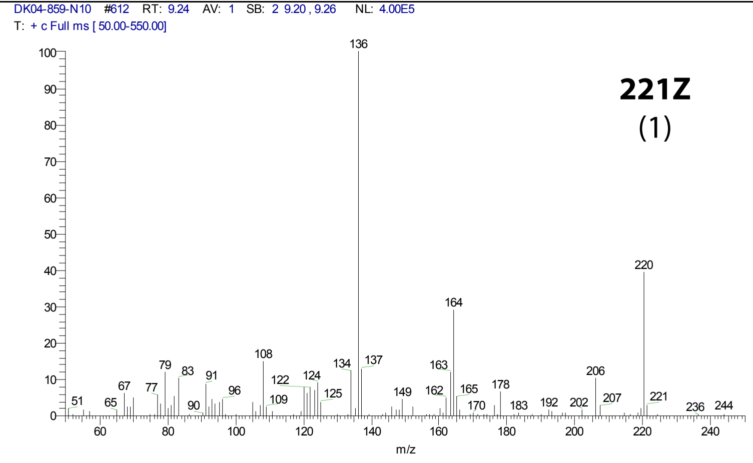
DK04-859-N10 #612 RT: 9.24 AV: 1 SB: 2 9.20 , 9.26 NL: 4.00E5
T: + c Full ms [50.00-550.00]
221Z
(1)
51 65 67 77 79 83 90 91 96 108 109 122 124 125 134 136 137 149 162 163 164 165 170 178 183 192 202 206 207 220 221 236 244
m/z

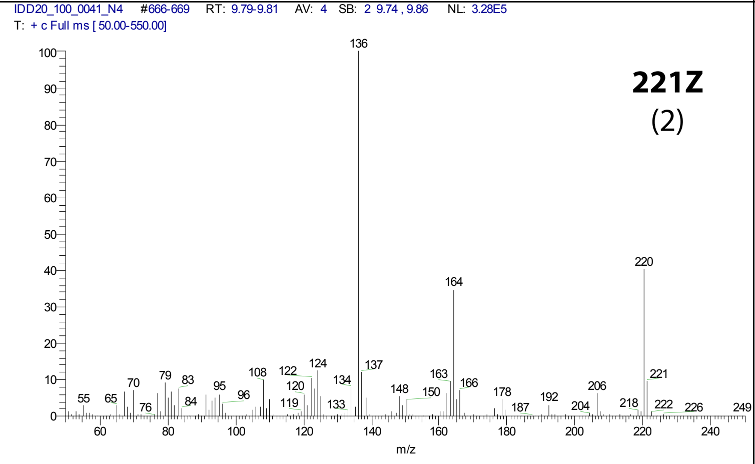
IDD20_100_0041_N4 #666-669 RT: 9.79-9.81 AV: 4 SB: 2 9.74 , 9.86 NL: 3.28E5
T: + c Full ms [50.00-550.00]
221Z
(2)
55 65 70 76 79 83 84 95 96 108 119 120 122 124 133 134 136 137 148 150 163 164 166 178 187 192 204 206 218 220 221 222 226 249
m/z

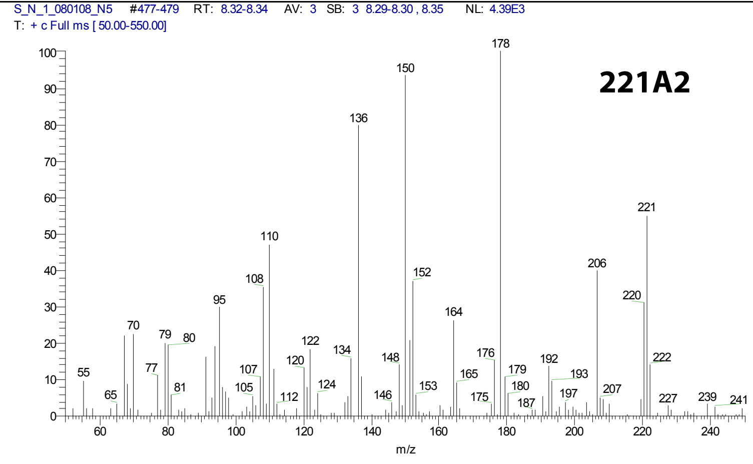
S_N_1_080108_N5 #477-479 RT: 8.32-8.34 AV: 3 SB: 3 8.29-8.30 , 8.35 NL: 4.39E3
T: + c Full ms [50.00-550.00]
221A2
55 65 70 77 79 80 81 95 105 107 108 110 112 120 122 124 134 136 146 148 150 152 153 164 165 175 176 178 179 180 187 192 193 197 206 207 220 221 222 227 239 241
m/z
