# Supplementary material for: Alkaloids from single skins of the Argentinian toad Melanophryniscus rubriventris (ANURA, BUFONIDAE): An unexpected variability in alkaloid profiles and a profusion of new structures
Source: Springerplus. 2012 Nov 23;1(1):51. doi: 10.1186/2193-1801-1-51 (PMC3625416; doi:10.1186/2193-1801-1-51)
Supplement: Supplementary file 4 — Additional fle 3 Figures S1-S10.: Total mass spectral ion current chromatograms for the alkaloid extracts of toad skin samples #1-10. (ZIP 12984 kb) (ZIP 9566 kb) (ZIP 13 MB) [file 40064_2012_198_MOESM4_ESM.zip › add3/1118854145799791_fig16.pdf]

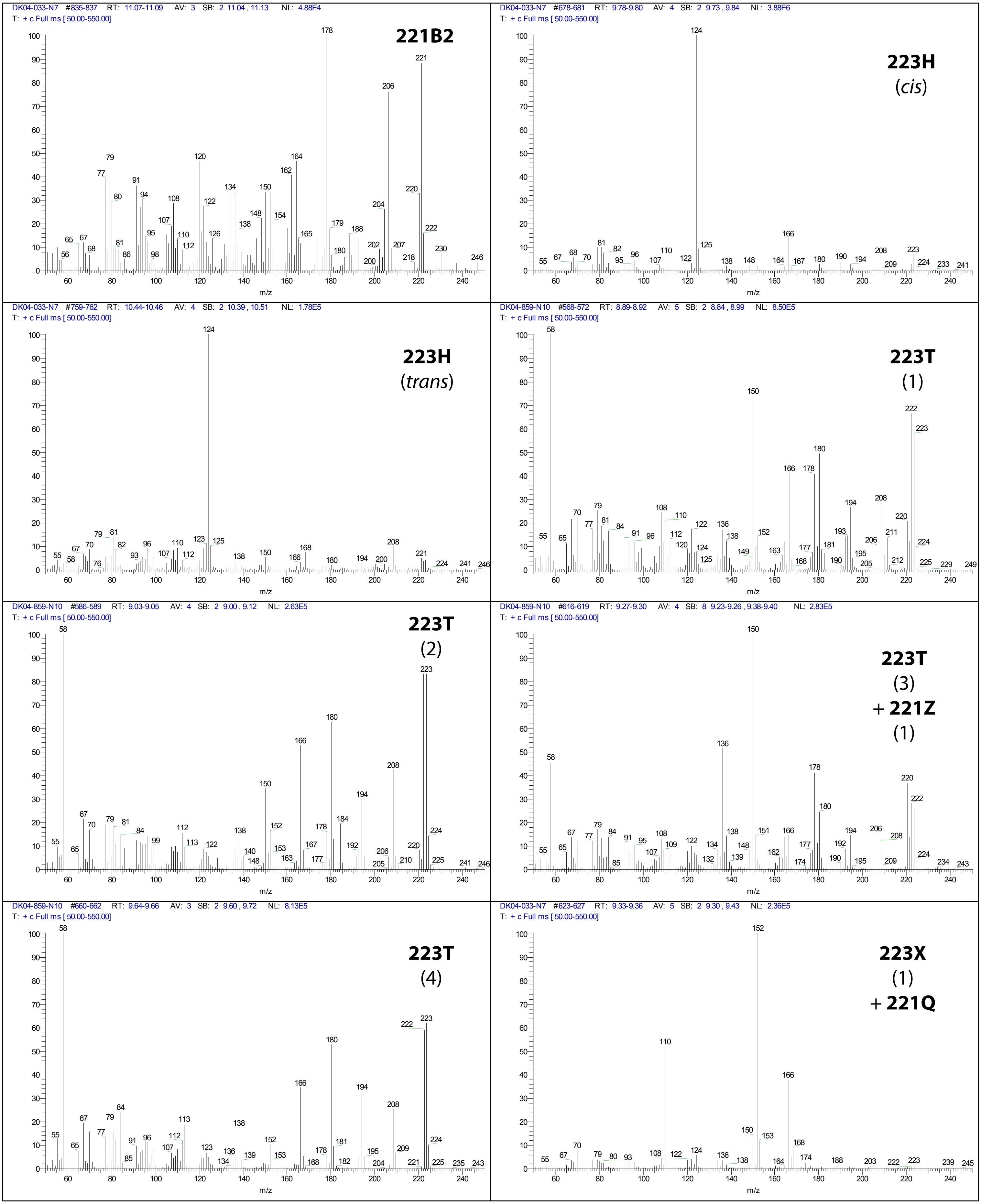
DK04-033-N7 #835-837 RT: 11.07-11.09 AV: 3 SB: 2 11.04 , 11.13 NL: 4.88E4
T: + c Full ms [50.00-550.00]
221B2
DK04-033-N7 #678-681 RT: 9.78-9.80 AV: 4 SB: 2 9.73 , 9.84 NL: 3.88E6
T: + c Full ms [50.00-550.00]
223H
(cis)
DK04-033-N7 #759-762 RT: 10.44-10.46 AV: 4 SB: 2 10.39 , 10.51 NL: 1.78E5
T: + c Full ms [50.00-550.00]
223H
(trans)
DK04-859-N10 #568-572 RT: 8.89-8.92 AV: 5 SB: 2 8.84 , 8.99 NL: 8.50E5
T: + c Full ms [50.00-550.00]
223T
(1)
DK04-859-N10 #586-589 RT: 9.03-9.05 AV: 4 SB: 2 9.00 , 9.12 NL: 2.63E5
T: + c Full ms [50.00-550.00]
223T
(2)
DK04-859-N10 #616-619 RT: 9.27-9.30 AV: 4 SB: 8 9.23-9.26 , 9.38-9.40 NL: 2.83E5
T: + c Full ms [50.00-550.00]
223T
(3)
+ 221Z
(1)
DK04-859-N10 #660-662 RT: 9.64-9.66 AV: 3 SB: 2 9.60 , 9.72 NL: 8.13E5
T: + c Full ms [50.00-550.00]
223T
(4)
DK04-033-N7 #623-627 RT: 9.33-9.36 AV: 5 SB: 2 9.30 , 9.43 NL: 2.36E5
T: + c Full ms [50.00-550.00]
223X
(1)
+ 221Q
m/z
