# Supplementary material for: Alkaloids from single skins of the Argentinian toad Melanophryniscus rubriventris (ANURA, BUFONIDAE): An unexpected variability in alkaloid profiles and a profusion of new structures
Source: Springerplus. 2012 Nov 23;1(1):51. doi: 10.1186/2193-1801-1-51 (PMC3625416; doi:10.1186/2193-1801-1-51)
Supplement: Supplementary file 4 — Additional fle 3 Figures S1-S10.: Total mass spectral ion current chromatograms for the alkaloid extracts of toad skin samples #1-10. (ZIP 12984 kb) (ZIP 9566 kb) (ZIP 13 MB) [file 40064_2012_198_MOESM4_ESM.zip › add3/1118854145799791_fig17.pdf]

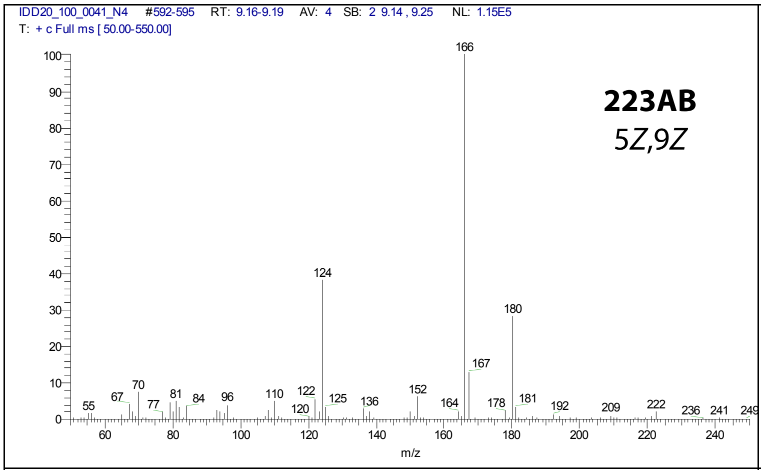
IDD20_100_0041_N4 #592-595 RT: 9.16-9.19 AV: 4 SB: 2 9.14 , 9.25 NL: 1.15E5
T: + c Full ms [50.00-550.00]
223AB
5Z,9Z
m/z

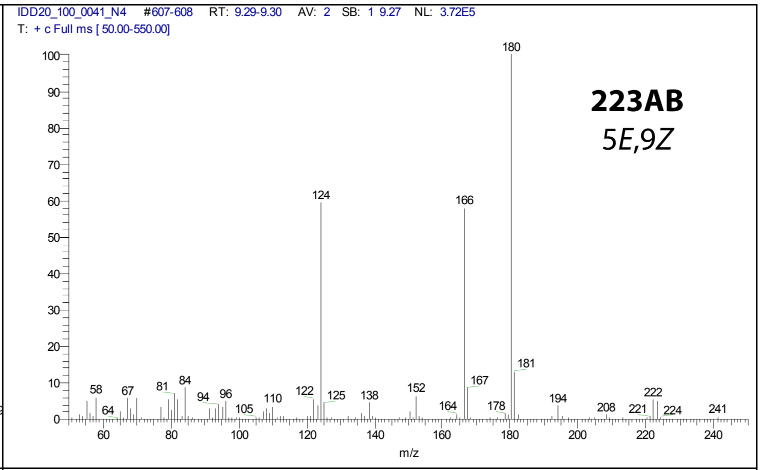
IDD20_100_0041_N4 #607-608 RT: 9.29-9.30 AV: 2 SB: 1 9.27 NL: 3.72E5
T: + c Full ms [50.00-550.00]
223AB
5E,9Z
m/z

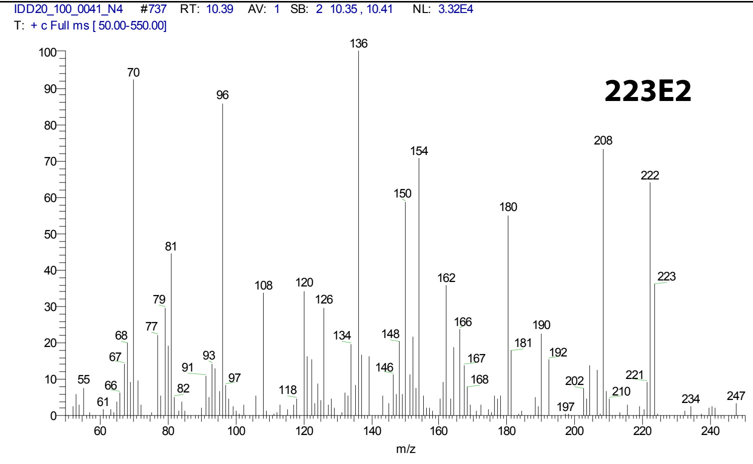
IDD20_100_0041_N4 #737 RT: 10.39 AV: 1 SB: 2 10.35 , 10.41 NL: 3.32E4
T: + c Full ms [50.00-550.00]
223E2
m/z

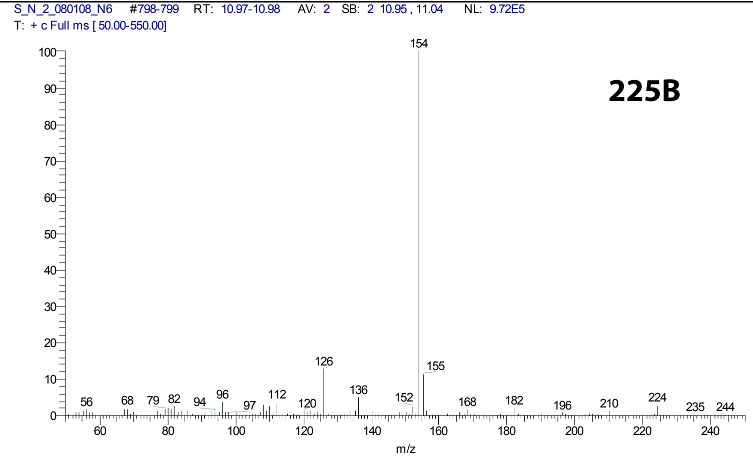
S_N_2_080108_N6 #798-799 RT: 10.97-10.98 AV: 2 SB: 2 10.95 , 11.04 NL: 9.72E5
T: + c Full ms [50.00-550.00]
225B
m/z

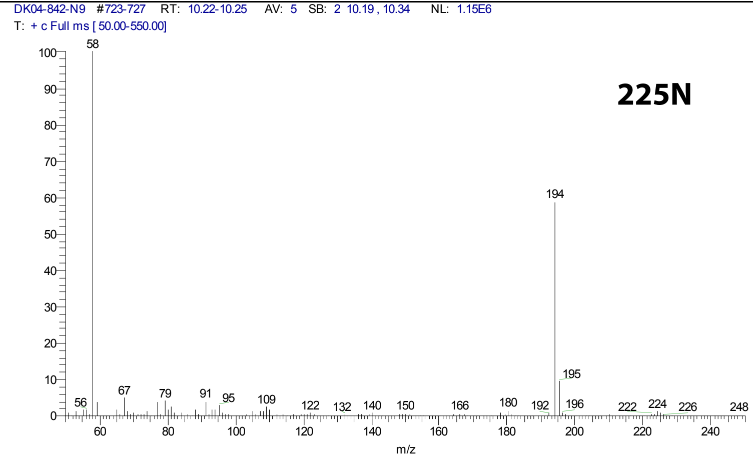
DK04-842-N9 #723-727 RT: 10.22-10.25 AV: 5 SB: 2 10.19 , 10.34 NL: 1.15E6
T: + c Full ms [50.00-550.00]
225N
m/z

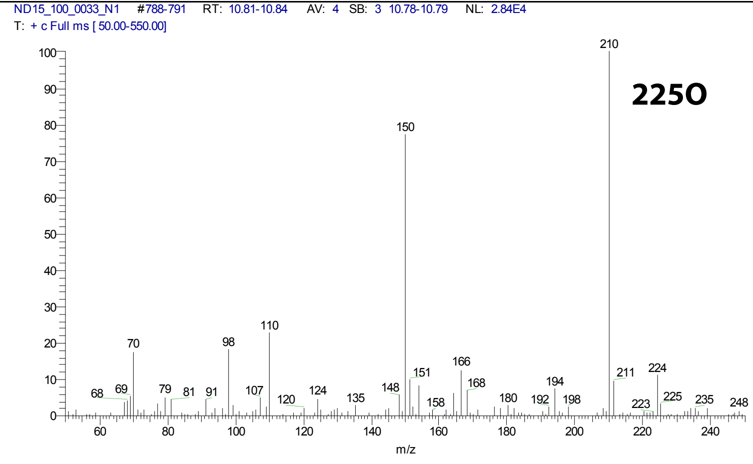
ND15_100_0033_N1 #788-791 RT: 10.81-10.84 AV: 4 SB: 3 10.78-10.79 NL: 2.84E4
T: + c Full ms [50.00-550.00]
225O
m/z

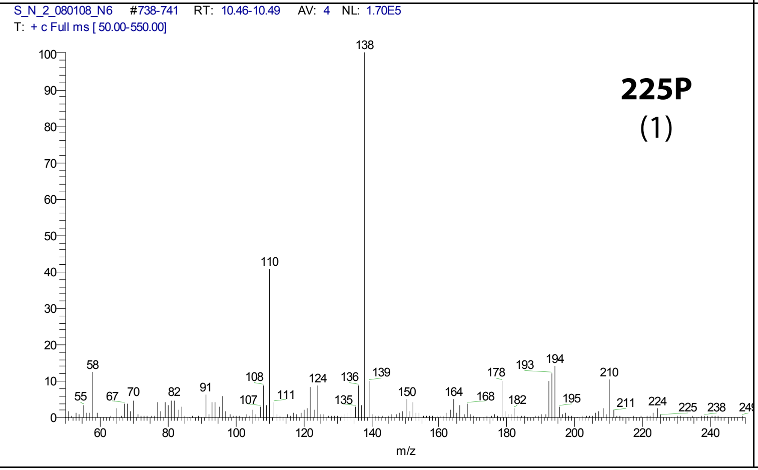
S_N_2_080108_N6 #738-741 RT: 10.46-10.49 AV: 4 NL: 1.70E5
T: + c Full ms [50.00-550.00]
225P
(1)
m/z

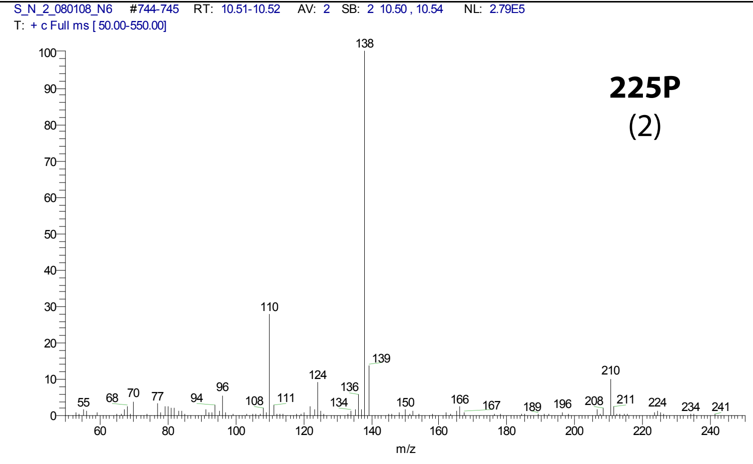
S_N_2_080108_N6 #744-745 RT: 10.51-10.52 AV: 2 SB: 2 10.50 , 10.54 NL: 2.79E5
T: + c Full ms [50.00-550.00]
225P
(2)
m/z
