# Supplementary material for: Alkaloids from single skins of the Argentinian toad Melanophryniscus rubriventris (ANURA, BUFONIDAE): An unexpected variability in alkaloid profiles and a profusion of new structures
Source: Springerplus. 2012 Nov 23;1(1):51. doi: 10.1186/2193-1801-1-51 (PMC3625416; doi:10.1186/2193-1801-1-51)
Supplement: Supplementary file 4 — Additional fle 3 Figures S1-S10.: Total mass spectral ion current chromatograms for the alkaloid extracts of toad skin samples #1-10. (ZIP 12984 kb) (ZIP 9566 kb) (ZIP 13 MB) [file 40064_2012_198_MOESM4_ESM.zip › add3/1118854145799791_fig18.pdf]

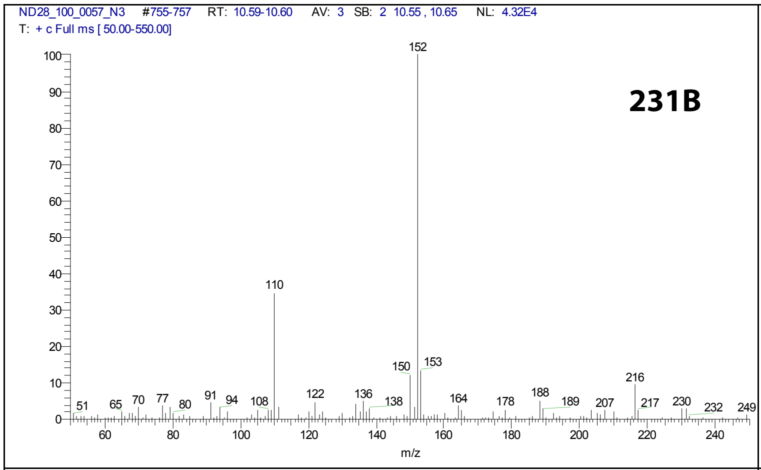
ND28_100_0057_N3 #755-757 RT: 10.59-10.60 AV: 3 SB: 2 10.55 , 10.65 NL: 4.32E4
T: + c Full ms [50.00-550.00]
231B
m/z

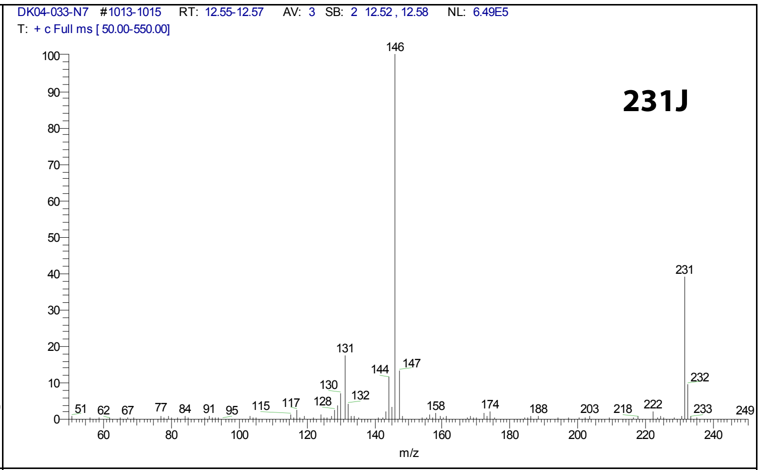
DK04-033-N7 #1013-1015 RT: 12.55-12.57 AV: 3 SB: 2 12.52 , 12.58 NL: 6.49E5
T: + c Full ms [50.00-550.00]
231J
m/z

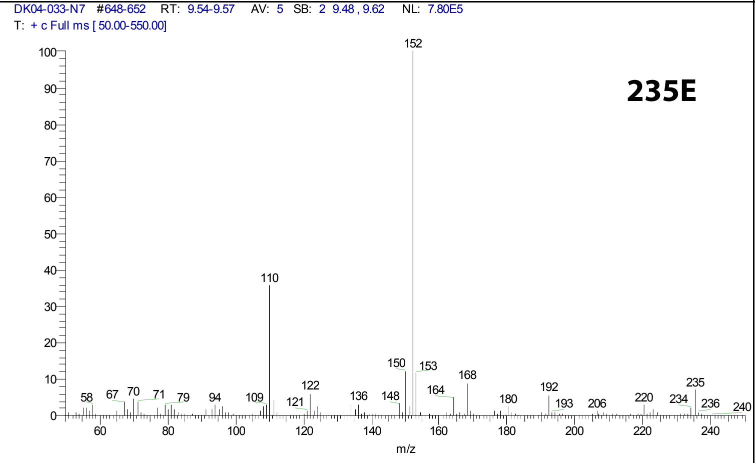
DK04-033-N7 #648-652 RT: 9.54-9.57 AV: 5 SB: 2 9.48 , 9.62 NL: 7.80E5
T: + c Full ms [50.00-550.00]
235E
m/z

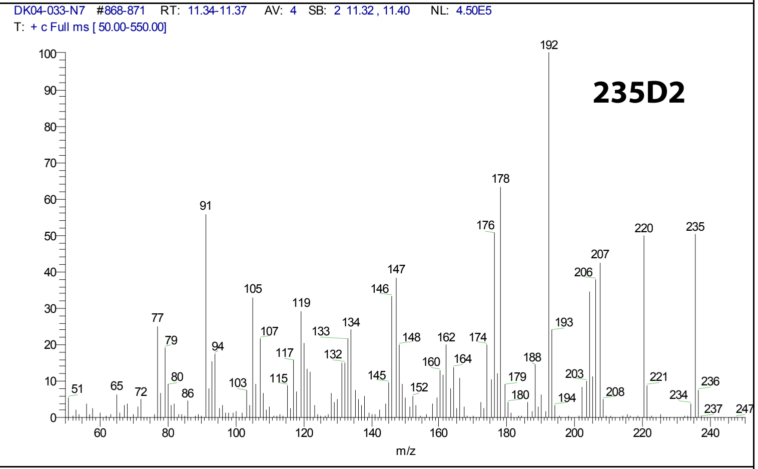
DK04-033-N7 #868-871 RT: 11.34-11.37 AV: 4 SB: 2 11.32 , 11.40 NL: 4.50E5
T: + c Full ms [50.00-550.00]
235D2
m/z

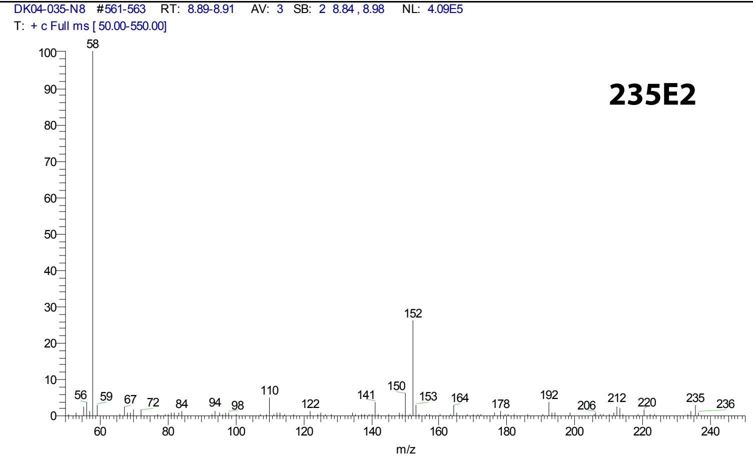
DK04-035-N8 #561-563 RT: 8.89-8.91 AV: 3 SB: 2 8.84 , 8.98 NL: 4.09E5
T: + c Full ms [50.00-550.00]
235E2
m/z

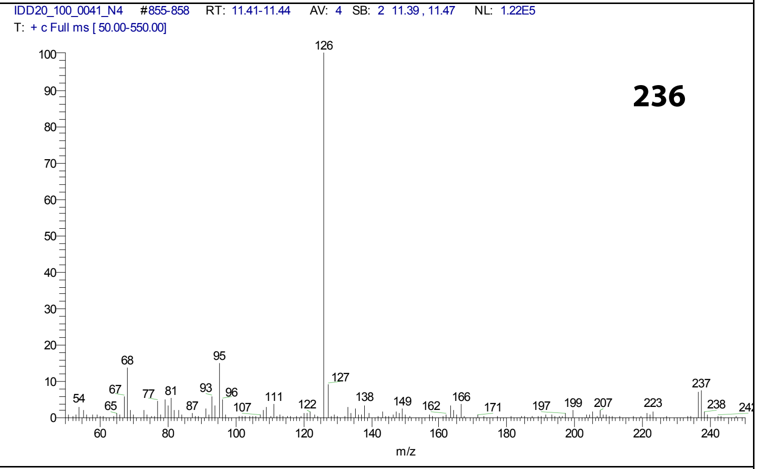
IDD20_100_0041_N4 #855-858 RT: 11.41-11.44 AV: 4 SB: 2 11.39 , 11.47 NL: 1.22E5
T: + c Full ms [50.00-550.00]
236
m/z

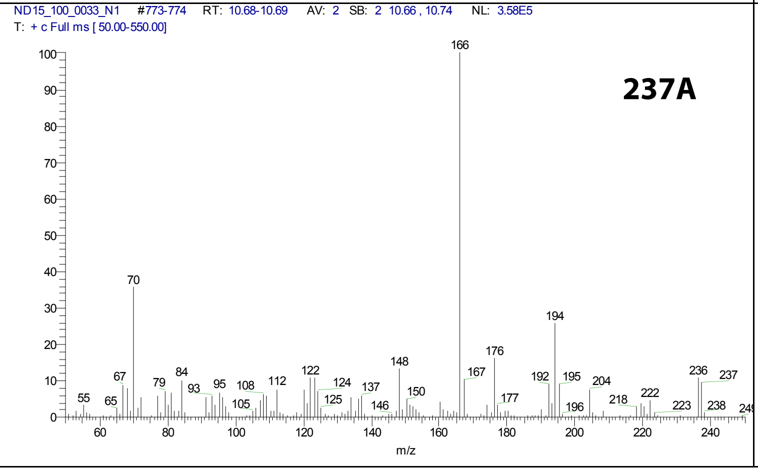
ND15_100_0033_N1 #773-774 RT: 10.68-10.69 AV: 2 SB: 2 10.66 , 10.74 NL: 3.58E5
T: + c Full ms [50.00-550.00]
237A
m/z

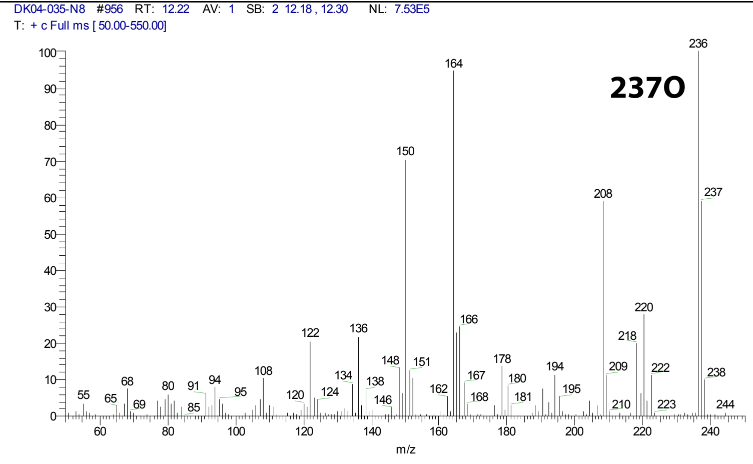
DK04-035-N8 #956 RT: 12.22 AV: 1 SB: 2 12.18 , 12.30 NL: 7.53E5
T: + c Full ms [50.00-550.00]
237O
m/z
