# Supplementary material for: Alkaloids from single skins of the Argentinian toad Melanophryniscus rubriventris (ANURA, BUFONIDAE): An unexpected variability in alkaloid profiles and a profusion of new structures
Source: Springerplus. 2012 Nov 23;1(1):51. doi: 10.1186/2193-1801-1-51 (PMC3625416; doi:10.1186/2193-1801-1-51)
Supplement: Supplementary file 4 — Additional fle 3 Figures S1-S10.: Total mass spectral ion current chromatograms for the alkaloid extracts of toad skin samples #1-10. (ZIP 12984 kb) (ZIP 9566 kb) (ZIP 13 MB) [file 40064_2012_198_MOESM4_ESM.zip › add3/1118854145799791_fig19.pdf]

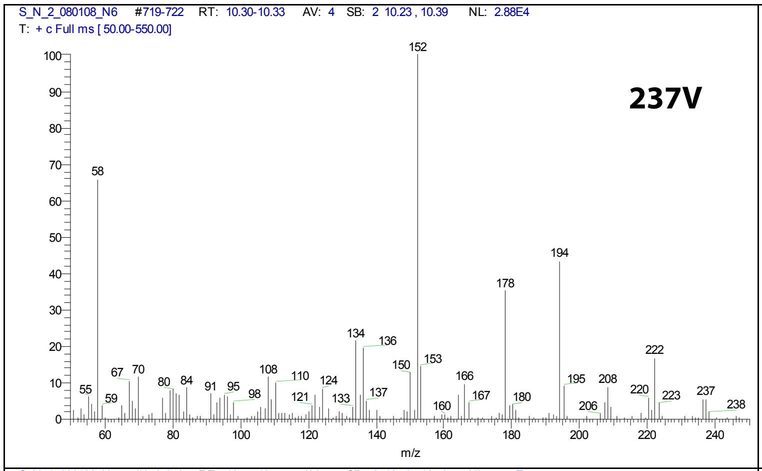
S_N_2_080108_N6 #719-722 RT: 10.30-10.33 AV: 4 SB: 2 10.23 , 10.39 NL: 2.88E4
T: + c Full ms [50.00-550.00]
237V
152
58
194
178
134 136
222
67 70 80 84 91 95 108 110 124 150 153 166 195 208 55 59 98 121 133 137 160 167 180 206 220 223 237 238
m/z

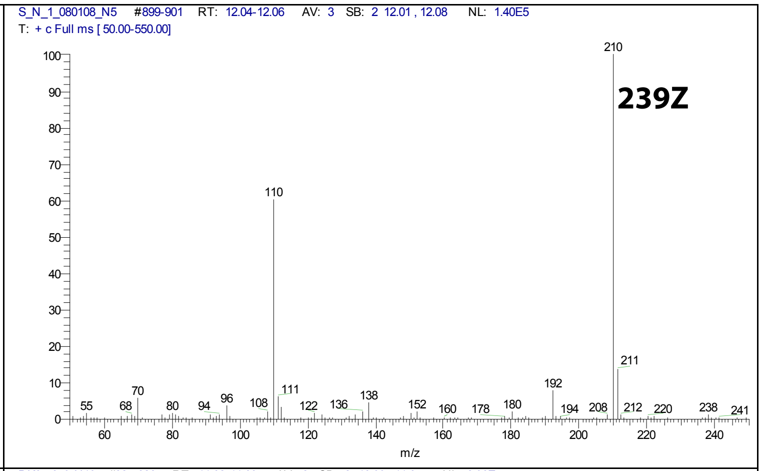
S_N_1_080108_N5 #899-901 RT: 12.04-12.06 AV: 3 SB: 2 12.01 , 12.08 NL: 1.40E5
T: + c Full ms [50.00-550.00]
239Z
210
110
211
192
55 68 70 80 94 96 108 111 122 136 138 152 160 178 180 194 208 212 220 238 241
m/z

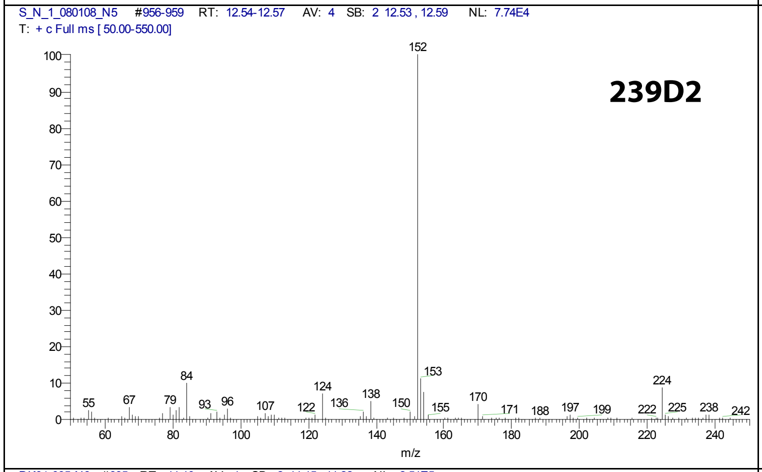
S_N_1_080108_N5 #956-959 RT: 12.54-12.57 AV: 4 SB: 2 12.53 , 12.59 NL: 7.74E4
T: + c Full ms [50.00-550.00]
239D2
152
153
224
55 67 79 84 93 96 107 122 124 136 138 150 155 170 171 188 197 199 222 225 238 242
m/z

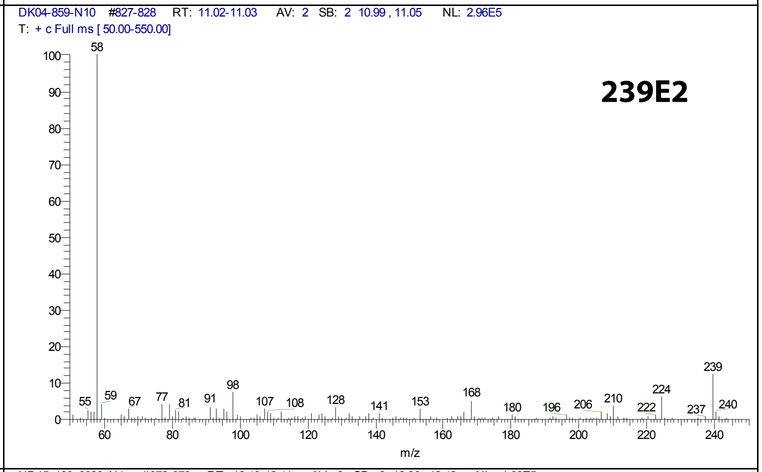
DK04-859-N10 #827-828 RT: 11.02-11.03 AV: 2 SB: 2 10.99 , 11.05 NL: 2.96E5
T: + c Full ms [50.00-550.00]
239E2
58
239
55 59 67 77 81 91 98 107 108 128 141 153 168 180 196 206 210 222 224 237 240
m/z

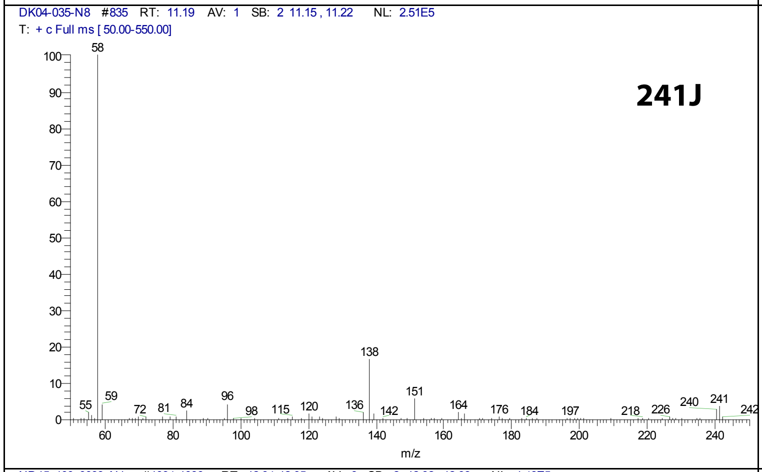
DK04-035-N8 #835 RT: 11.19 AV: 1 SB: 2 11.15 , 11.22 NL: 2.51E5
T: + c Full ms [50.00-550.00]
241J
58
138
55 59 72 81 84 96 98 115 120 136 142 151 164 176 184 197 218 226 240 241 242
m/z

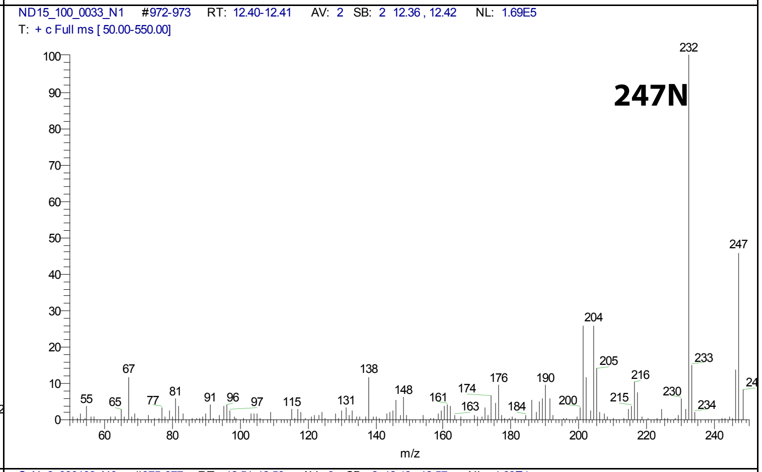
ND15_100_0033_N1 #972-973 RT: 12.40-12.41 AV: 2 SB: 2 12.36 , 12.42 NL: 1.69E5
T: + c Full ms [50.00-550.00]
247N
232
247
204
205 216 233
55 65 67 77 81 91 96 97 115 131 138 148 161 163 174 176 184 190 200 215 230 234
m/z

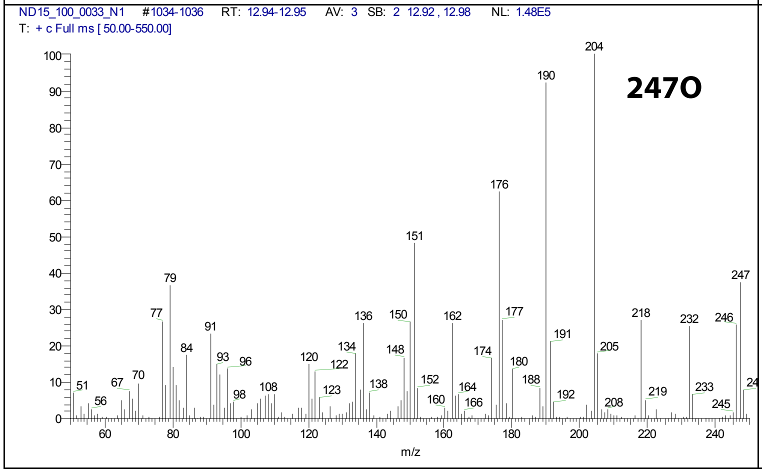
ND15_100_0033_N1 #1034-1036 RT: 12.94-12.95 AV: 3 SB: 2 12.92 , 12.98 NL: 1.48E5
T: + c Full ms [50.00-550.00]
247O
204
190
176
151
247
79
77 91 136 150 162 177 218 232 246
51 56 67 70 84 93 96 98 108 120 122 123 134 138 148 152 160 164 166 174 180 188 191 192 205 208 219 233 245
m/z

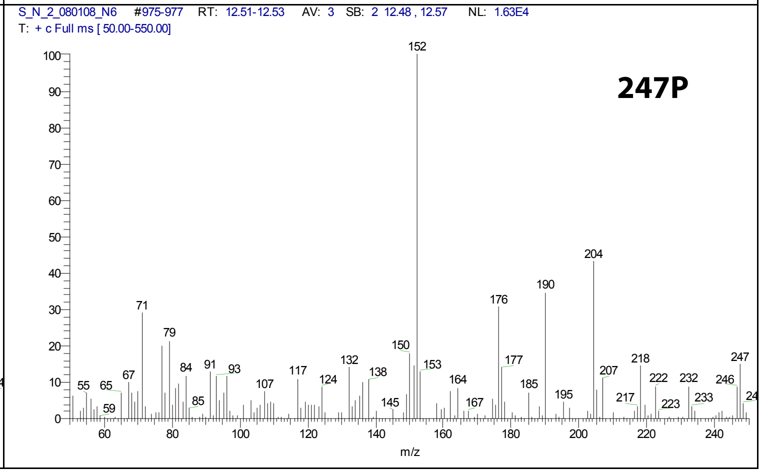
S_N_2_080108_N6 #975-977 RT: 12.51-12.53 AV: 3 SB: 2 12.48 , 12.57 NL: 1.63E4
T: + c Full ms [50.00-550.00]
247P
152
204
190
176
71 79
55 59 65 67 84 85 91 93 107 117 124 132 138 145 150 153 164 167 177 185 195 207 217 218 222 223 232 233 246 247
m/z
