# Supplementary material for: Alkaloids from single skins of the Argentinian toad Melanophryniscus rubriventris (ANURA, BUFONIDAE): An unexpected variability in alkaloid profiles and a profusion of new structures
Source: Springerplus. 2012 Nov 23;1(1):51. doi: 10.1186/2193-1801-1-51 (PMC3625416; doi:10.1186/2193-1801-1-51)
Supplement: Supplementary file 4 — Additional fle 3 Figures S1-S10.: Total mass spectral ion current chromatograms for the alkaloid extracts of toad skin samples #1-10. (ZIP 12984 kb) (ZIP 9566 kb) (ZIP 13 MB) [file 40064_2012_198_MOESM4_ESM.zip › add3/1118854145799791_fig21.pdf]

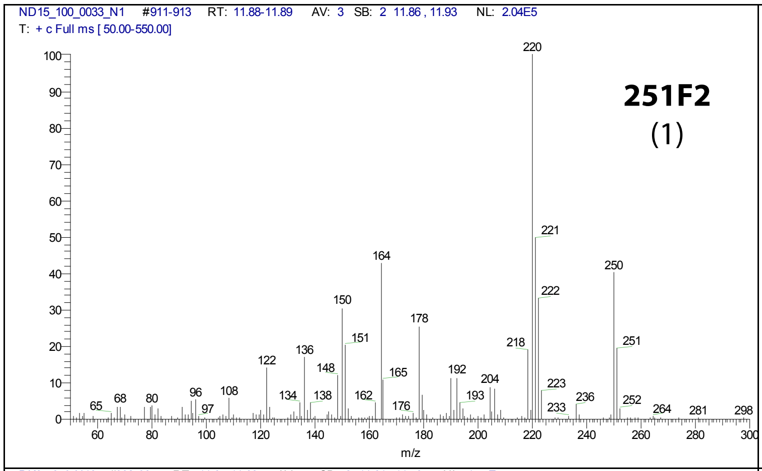
ND15_100_0033_N1 #911-913 RT: 11.88-11.89 AV: 3 SB: 2 11.86 , 11.93 NL: 2.04E5
T: + c Full ms [50.00-550.00]
251F2
(1)
220
221
164
250
222
150
178
151
136
122
148
165
192
204
218
223
65
68
80
96
97
108
134
138
162
176
193
233
236
252
264
281
298
m/z

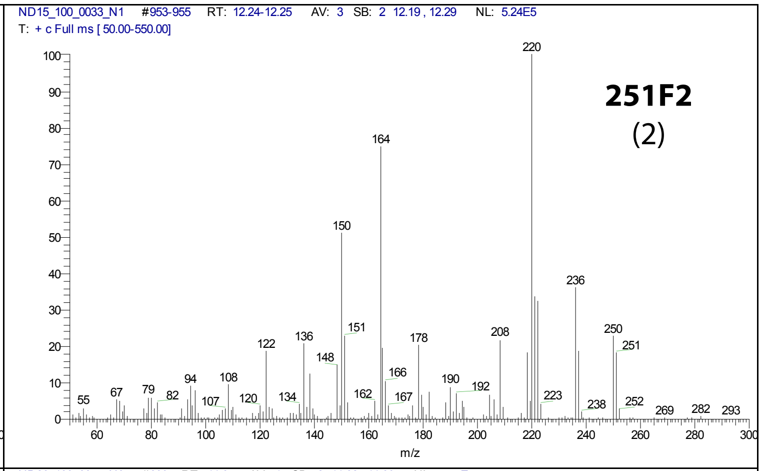
ND15_100_0033_N1 #953-955 RT: 12.24-12.25 AV: 3 SB: 2 12.19 , 12.29 NL: 5.24E5
T: + c Full ms [50.00-550.00]
251F2
(2)
220
164
150
236
250
251
208
178
151
136
122
148
166
190
192
94
108
67
79
82
107
120
134
162
167
223
238
252
55
269
282
293
m/z

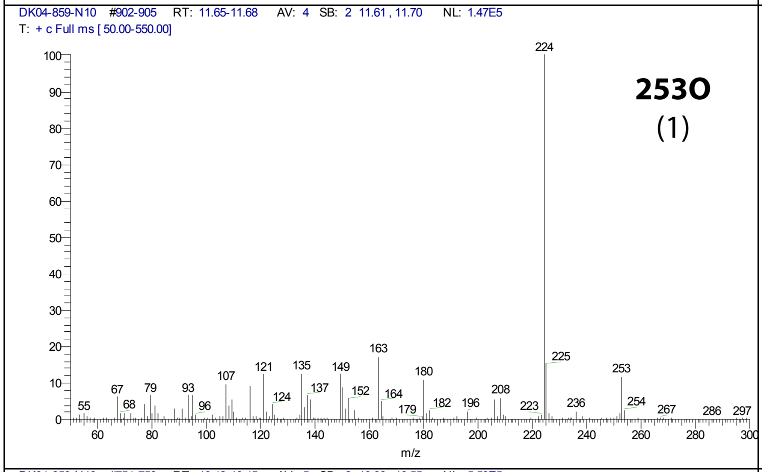
DK04-859-N10 #902-905 RT: 11.65-11.68 AV: 4 SB: 2 11.61 , 11.70 NL: 1.47E5
T: + c Full ms [50.00-550.00]
253O
(1)
224
225
253
163
180
107
121
135
149
137
124
152
164
208
67
79
93
55
68
96
179
182
196
223
236
254
267
286
297
m/z

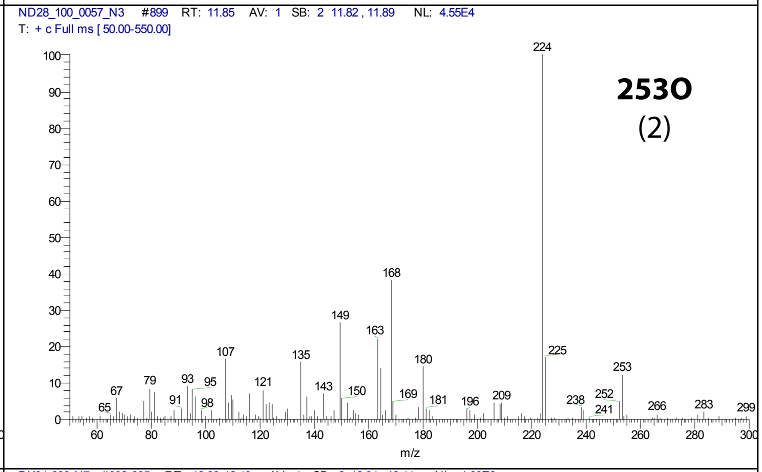
ND28_100_0057_N3 #899 RT: 11.85 AV: 1 SB: 2 11.82 , 11.89 NL: 4.55E4
T: + c Full ms [50.00-550.00]
253O
(2)
224
168
149
163
225
253
180
107
135
121
143
150
169
181
196
209
79
93
95
67
91
98
65
238
252
241
266
283
299
m/z

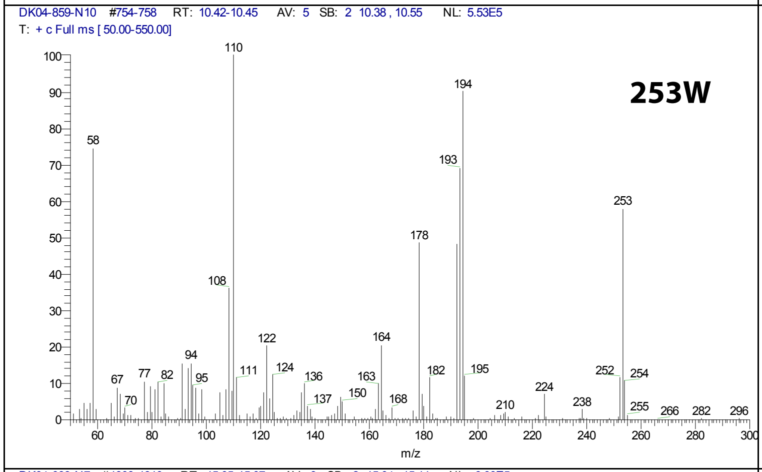
DK04-859-N10 #754-758 RT: 10.42-10.45 AV: 5 SB: 2 10.38 , 10.55 NL: 5.53E5
T: + c Full ms [50.00-550.00]
253W
110
194
58
193
253
178
108
122
164
94
67
77
82
95
70
111
124
136
137
150
163
168
182
195
210
224
238
252
254
255
266
282
296
m/z

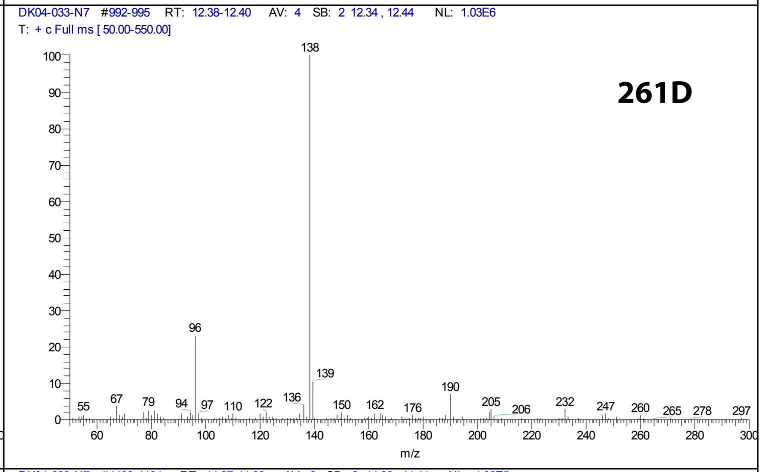
DK04-033-N7 #992-995 RT: 12.38-12.40 AV: 4 SB: 2 12.34 , 12.44 NL: 1.03E6
T: + c Full ms [50.00-550.00]
261D
138
96
139
190
55
67
79
94
97
110
122
136
150
162
176
205
206
232
247
260
265
278
297
m/z

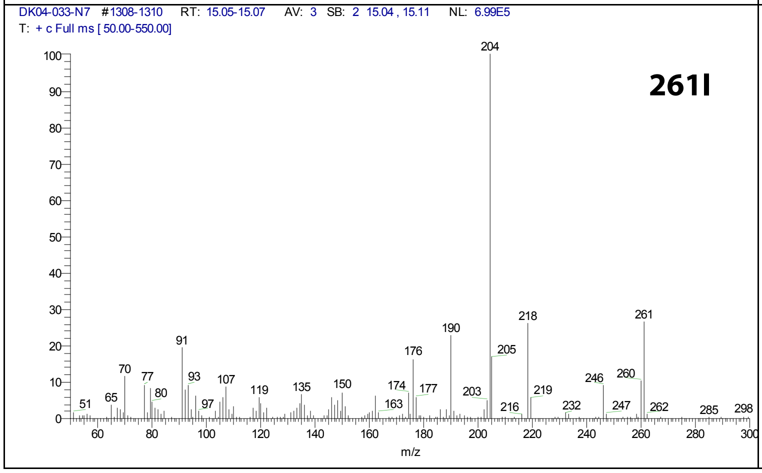
DK04-033-N7 #1308-1310 RT: 15.05-15.07 AV: 3 SB: 2 15.04 , 15.11 NL: 6.99E5
T: + c Full ms [50.00-550.00]
261I
204
91
190
218
261
176
205
70
77
93
80
107
119
135
150
174
163
177
203
216
219
232
246
260
247
262
51
65
97
285
298
m/z

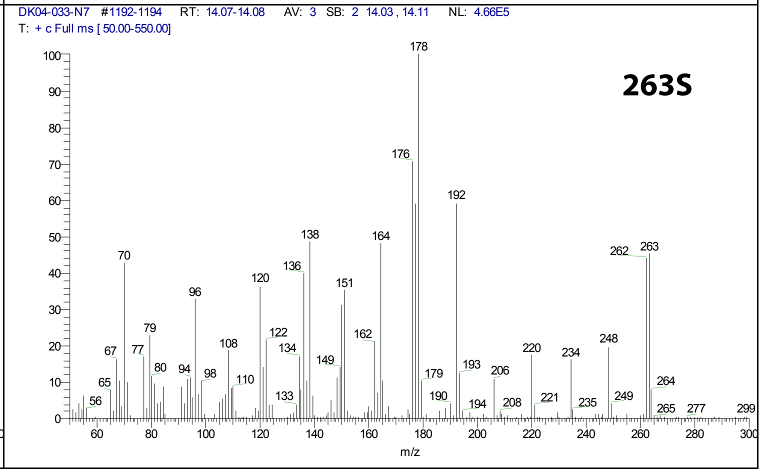
DK04-033-N7 #1192-1194 RT: 14.07-14.08 AV: 3 SB: 2 14.03 , 14.11 NL: 4.66E5
T: + c Full ms [50.00-550.00]
263S
178
176
192
138
164
70
136
120
96
151
262
263
79
67
77
108
122
134
162
248
220
234
65
56
80
94
98
110
133
149
179
193
206
190
194
208
221
235
249
264
265
277
299
m/z
