# Supplementary material for: Alkaloids from single skins of the Argentinian toad Melanophryniscus rubriventris (ANURA, BUFONIDAE): An unexpected variability in alkaloid profiles and a profusion of new structures
Source: Springerplus. 2012 Nov 23;1(1):51. doi: 10.1186/2193-1801-1-51 (PMC3625416; doi:10.1186/2193-1801-1-51)
Supplement: Supplementary file 4 — Additional fle 3 Figures S1-S10.: Total mass spectral ion current chromatograms for the alkaloid extracts of toad skin samples #1-10. (ZIP 12984 kb) (ZIP 9566 kb) (ZIP 13 MB) [file 40064_2012_198_MOESM4_ESM.zip › add3/1118854145799791_fig22.pdf]

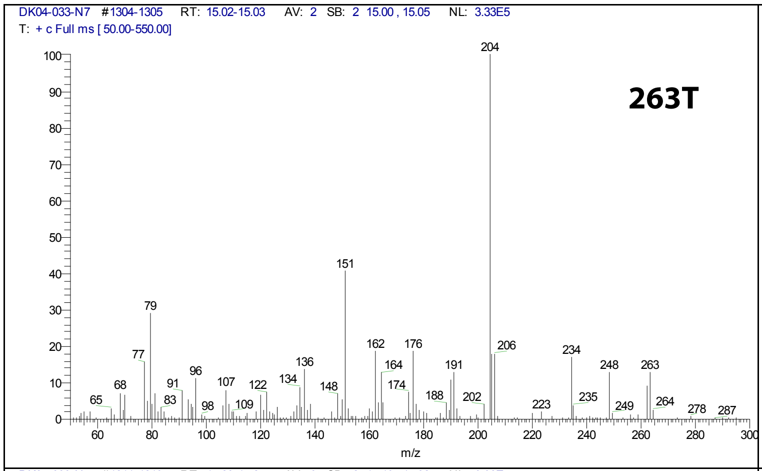
DK04-033-N7 #1304-1305 RT: 15.02-15.03 AV: 2 SB: 2 15.00 , 15.05 NL: 3.33E5
T: + c Full ms [50.00-550.00]
263T
204
151
79
77
96
91
83
98
107
109
122
134
136
148
162
164
174
176
188
191
202
206
223
234
235
248
249
263
264
278
287
65
68
m/z

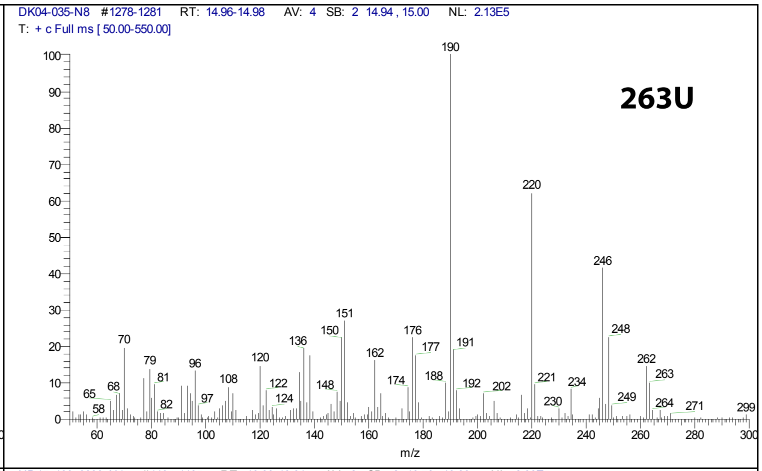
DK04-035-N8 #1278-1281 RT: 14.96-14.98 AV: 4 SB: 2 14.94 , 15.00 NL: 2.13E5
T: + c Full ms [50.00-550.00]
263U
190
220
246
248
151
150
136
176
177
162
191
188
174
120
122
124
148
108
96
97
79
81
82
70
68
65
58
192
202
221
230
234
249
262
263
264
271
299
m/z

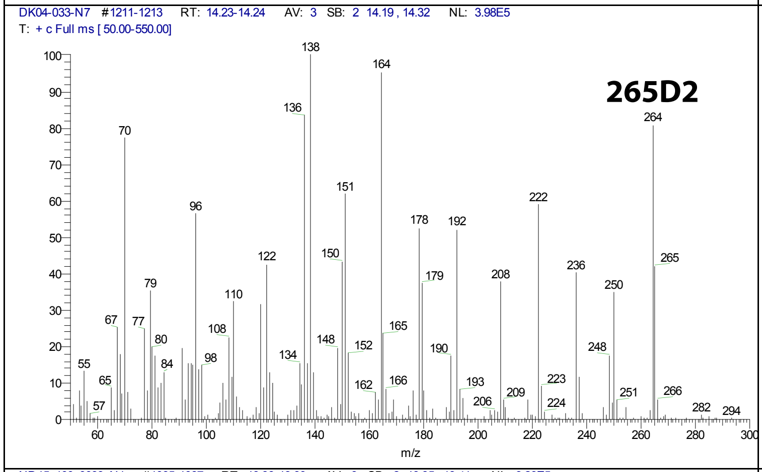
DK04-033-N7 #1211-1213 RT: 14.23-14.24 AV: 3 SB: 2 14.19 , 14.32 NL: 3.98E5
T: + c Full ms [50.00-550.00]
265D2
138
164
136
264
70
151
96
222
178
192
122
150
265
179
208
236
79
110
250
67
77
108
80
98
84
134
148
152
165
190
248
55
65
57
162
166
193
206
209
223
224
251
266
282
294
m/z

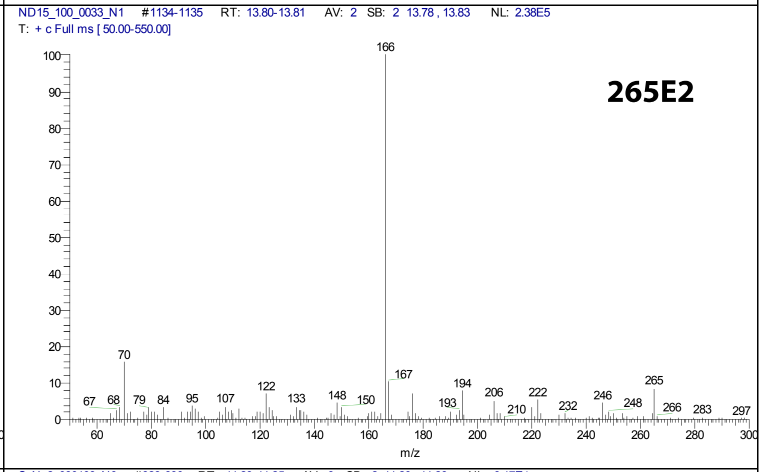
ND15_100_0033_N1 #1134-1135 RT: 13.80-13.81 AV: 2 SB: 2 13.78 , 13.83 NL: 2.38E5
T: + c Full ms [50.00-550.00]
265E2
166
70
167
67
68
79
84
95
107
122
133
148
150
193
194
206
210
222
232
246
248
265
266
283
297
m/z

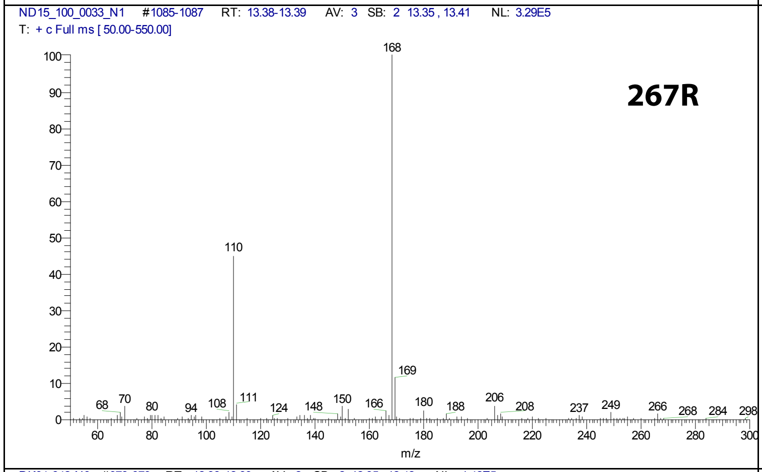
ND15_100_0033_N1 #1085-1087 RT: 13.38-13.39 AV: 3 SB: 2 13.35 , 13.41 NL: 3.29E5
T: + c Full ms [50.00-550.00]
267R
168
110
169
68
70
80
94
108
111
124
148
150
166
180
188
206
208
237
249
266
268
284
298
m/z

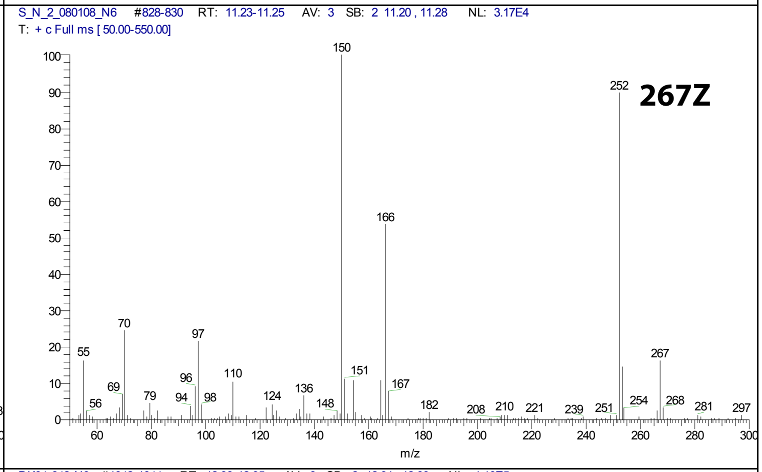
S_N_2_080108_N6 #828-830 RT: 11.23-11.25 AV: 3 SB: 2 11.20 , 11.28 NL: 3.17E4
T: + c Full ms [50.00-550.00]
267Z
150
252
166
70
97
55
96
110
69
56
79
94
98
124
136
148
151
167
182
208
210
221
239
251
254
267
268
281
297
m/z

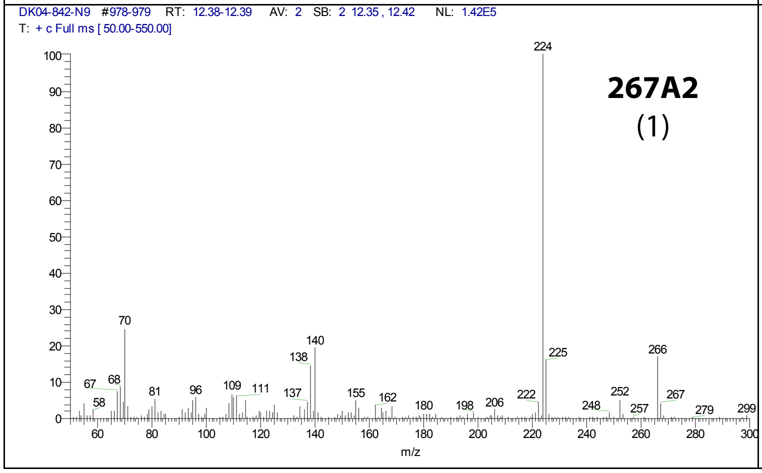
DK04-842-N9 #978-979 RT: 12.38-12.39 AV: 2 SB: 2 12.35 , 12.42 NL: 1.42E5
T: + c Full ms [50.00-550.00]
267A2
(1)
224
70
140
138
225
266
67
58
68
81
96
109
111
137
155
162
180
198
206
222
248
252
257
267
279
299
m/z

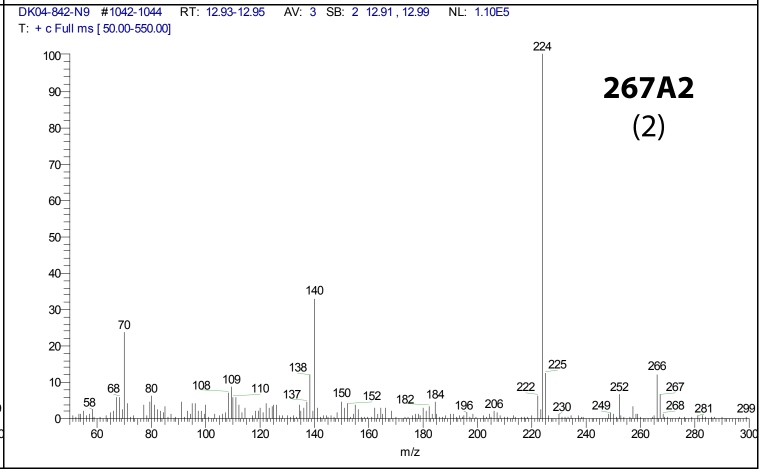
DK04-842-N9 #1042-1044 RT: 12.93-12.95 AV: 3 SB: 2 12.91 , 12.99 NL: 1.10E5
T: + c Full ms [50.00-550.00]
267A2
(2)
224
140
70
138
225
266
58
68
80
108
109
110
137
150
152
182
184
196
206
222
230
249
252
267
268
281
299
m/z
