# Supplementary material for: Alkaloids from single skins of the Argentinian toad Melanophryniscus rubriventris (ANURA, BUFONIDAE): An unexpected variability in alkaloid profiles and a profusion of new structures
Source: Springerplus. 2012 Nov 23;1(1):51. doi: 10.1186/2193-1801-1-51 (PMC3625416; doi:10.1186/2193-1801-1-51)
Supplement: Supplementary file 4 — Additional fle 3 Figures S1-S10.: Total mass spectral ion current chromatograms for the alkaloid extracts of toad skin samples #1-10. (ZIP 12984 kb) (ZIP 9566 kb) (ZIP 13 MB) [file 40064_2012_198_MOESM4_ESM.zip › add3/1118854145799791_fig23.pdf]

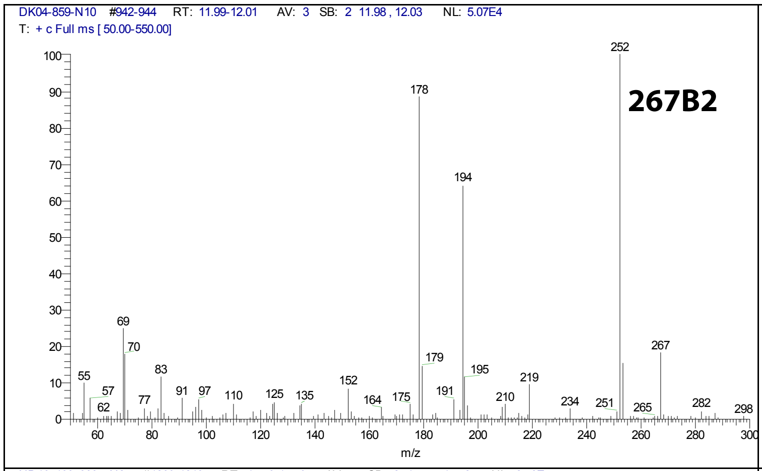
DK04-859-N10 #942-944 RT: 11.99-12.01 AV: 3 SB: 2 11.98 , 12.03 NL: 5.07E4
T: + c Full ms [50.00-550.00]
267B2
m/z

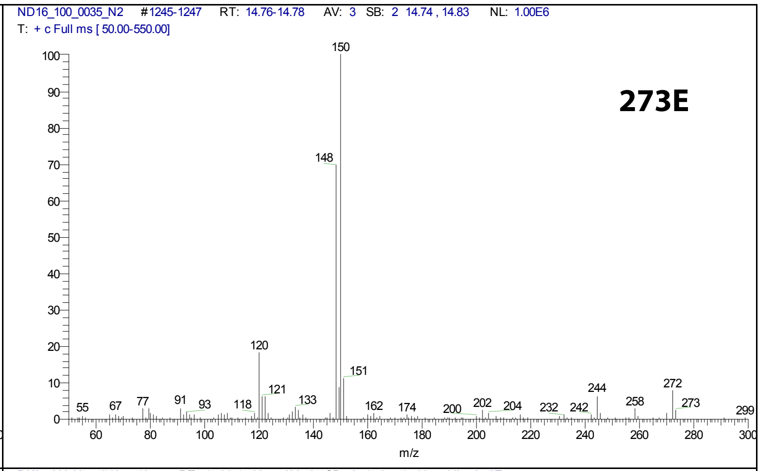
ND16_100_0035_N2 #1245-1247 RT: 14.76-14.78 AV: 3 SB: 2 14.74 , 14.83 NL: 1.00E6
T: + c Full ms [50.00-550.00]
273E
m/z

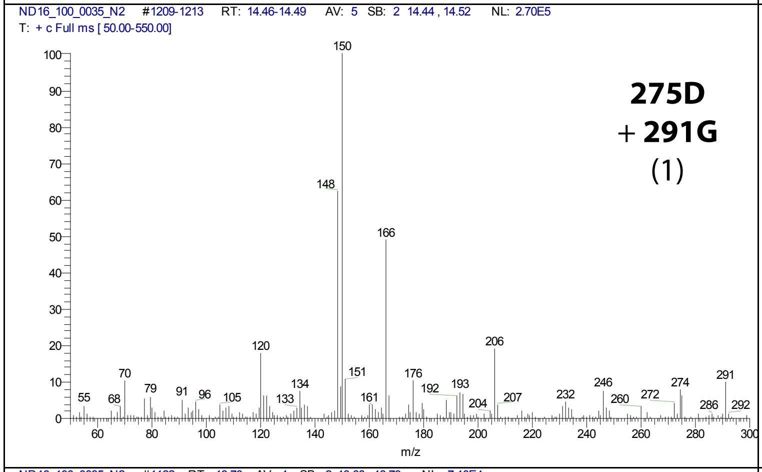
ND16_100_0035_N2 #1209-1213 RT: 14.46-14.49 AV: 5 SB: 2 14.44 , 14.52 NL: 2.70E5
T: + c Full ms [50.00-550.00]
275D
+ 291G
(1)
m/z

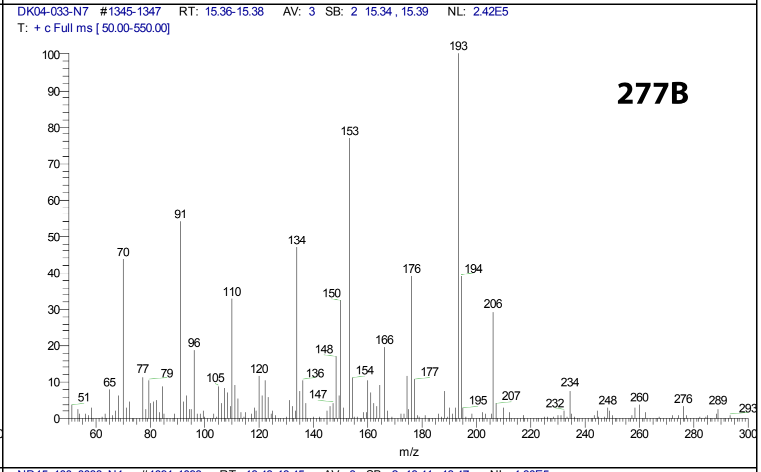
DK04-033-N7 #1345-1347 RT: 15.36-15.38 AV: 3 SB: 2 15.34 , 15.39 NL: 2.42E5
T: + c Full ms [50.00-550.00]
277B
m/z

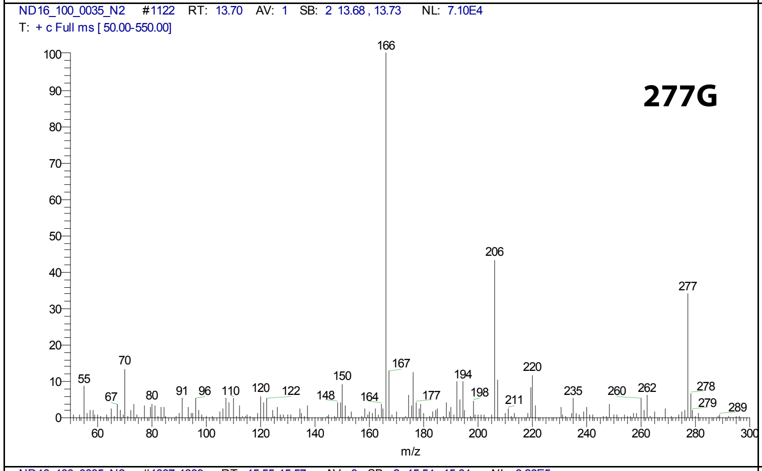
ND16_100_0035_N2 #1122 RT: 13.70 AV: 1 SB: 2 13.68 , 13.73 NL: 7.10E4
T: + c Full ms [50.00-550.00]
277G
m/z

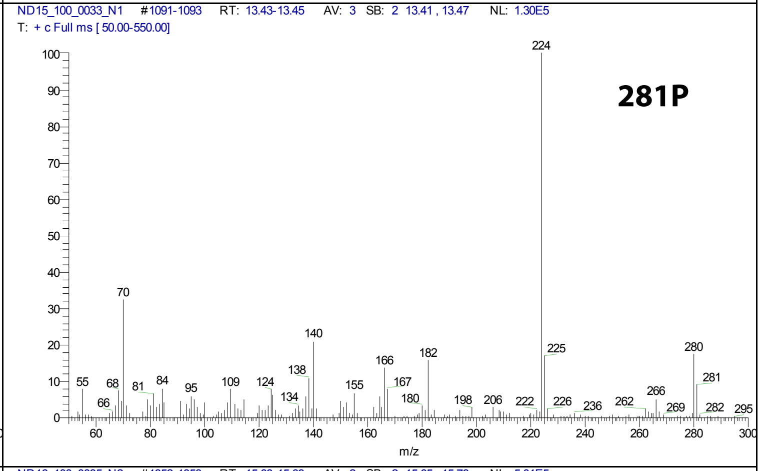
ND15_100_0033_N1 #1091-1093 RT: 13.43-13.45 AV: 3 SB: 2 13.41 , 13.47 NL: 1.30E5
T: + c Full ms [50.00-550.00]
281P
m/z

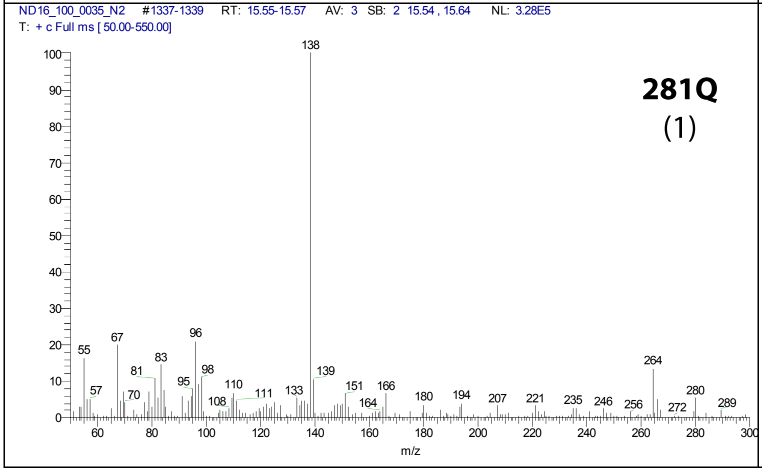
ND16_100_0035_N2 #1337-1339 RT: 15.55-15.57 AV: 3 SB: 2 15.54 , 15.64 NL: 3.28E5
T: + c Full ms [50.00-550.00]
281Q
(1)
m/z

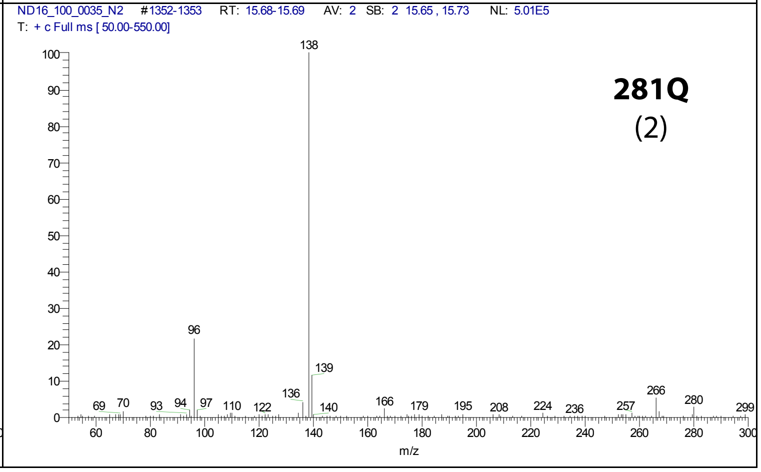
ND16_100_0035_N2 #1352-1353 RT: 15.68-15.69 AV: 2 SB: 2 15.65 , 15.73 NL: 5.01E5
T: + c Full ms [50.00-550.00]
281Q
(2)
m/z
