# Supplementary material for: Alkaloids from single skins of the Argentinian toad Melanophryniscus rubriventris (ANURA, BUFONIDAE): An unexpected variability in alkaloid profiles and a profusion of new structures
Source: Springerplus. 2012 Nov 23;1(1):51. doi: 10.1186/2193-1801-1-51 (PMC3625416; doi:10.1186/2193-1801-1-51)
Supplement: Supplementary file 4 — Additional fle 3 Figures S1-S10.: Total mass spectral ion current chromatograms for the alkaloid extracts of toad skin samples #1-10. (ZIP 12984 kb) (ZIP 9566 kb) (ZIP 13 MB) [file 40064_2012_198_MOESM4_ESM.zip › add3/1118854145799791_fig24.pdf]

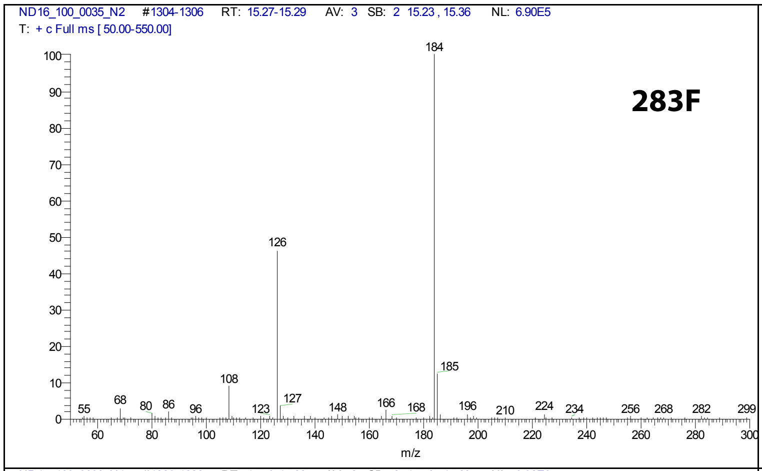
ND16_100_0035_N2 #1304-1306 RT: 15.27-15.29 AV: 3 SB: 2 15.23 , 15.36 NL: 6.90E5
T: + c Full ms [50.00-550.00]
283F
184
126
108
185
55 68 80 86 96 123 127 148 166 168 196 210 224 234 256 268 282 299
m/z

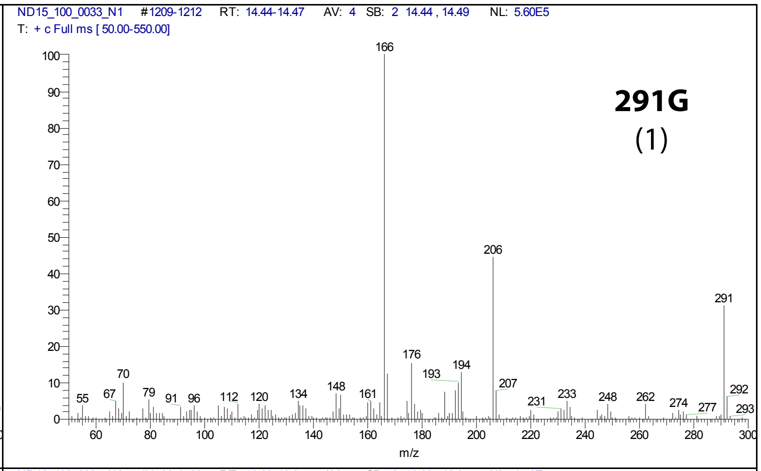
ND15_100_0033_N1 #1209-1212 RT: 14.44-14.47 AV: 4 SB: 2 14.44 , 14.49 NL: 5.60E5
T: + c Full ms [50.00-550.00]
291G
(1)
166
206
291
176
193 194
70
55 67 79 91 96 112 120 134 148 161 207 231 233 248 262 274 277 292 293
m/z

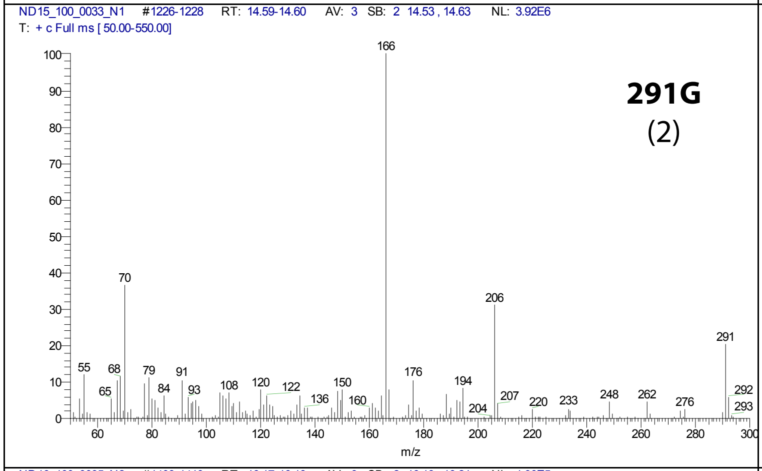
ND15_100_0033_N1 #1226-1228 RT: 14.59-14.60 AV: 3 SB: 2 14.53 , 14.63 NL: 3.92E6
T: + c Full ms [50.00-550.00]
291G
(2)
166
70
206
291
55 65 68 79 84 91 93 108 120 122 136 150 160 176 194 204 207 220 233 248 262 276 292 293
m/z

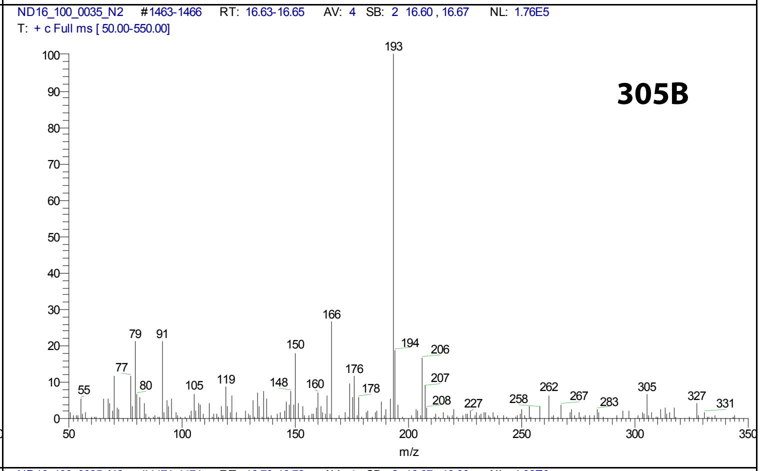
ND16_100_0035_N2 #1463-1466 RT: 16.63-16.65 AV: 4 SB: 2 16.60 , 16.67 NL: 1.76E5
T: + c Full ms [50.00-550.00]
305B
193
166
79 91
150
194 206
55 77 80 105 119 148 160 176 178 207 208 227 258 262 267 283 305 327 331
m/z

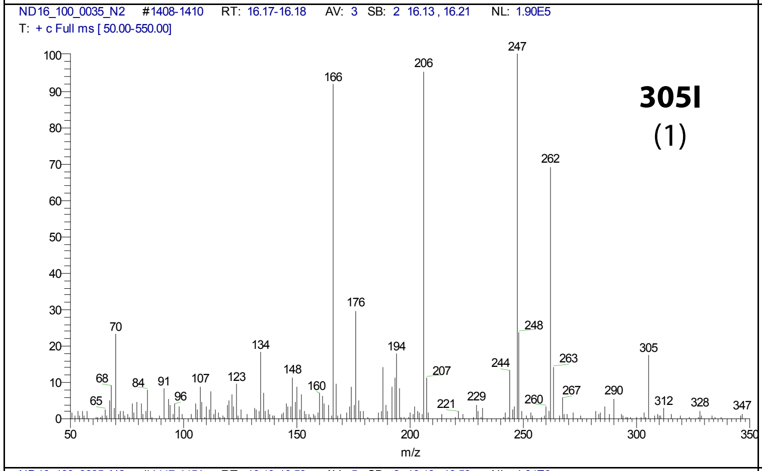
ND16_100_0035_N2 #1408-1410 RT: 16.17-16.18 AV: 3 SB: 2 16.13 , 16.21 NL: 1.90E5
T: + c Full ms [50.00-550.00]
305I
(1)
247
166
206
262
176
70
134
194
305
65 68 84 91 96 107 123 148 160 207 221 229 244 248 260 263 267 290 312 328 347
m/z

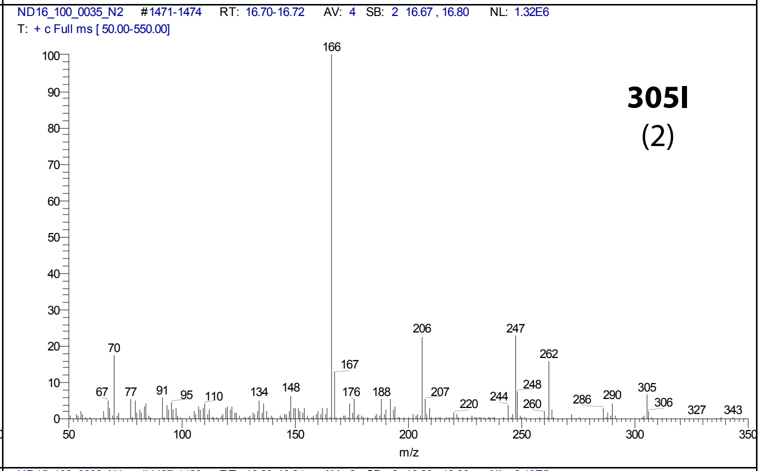
ND16_100_0035_N2 #1471-1474 RT: 16.70-16.72 AV: 4 SB: 2 16.67 , 16.80 NL: 1.32E6
T: + c Full ms [50.00-550.00]
305I
(2)
166
206 247
262
70
67 77 91 95 110 134 148 167 176 188 207 220 244 248 260 286 290 305 306 327 343
m/z

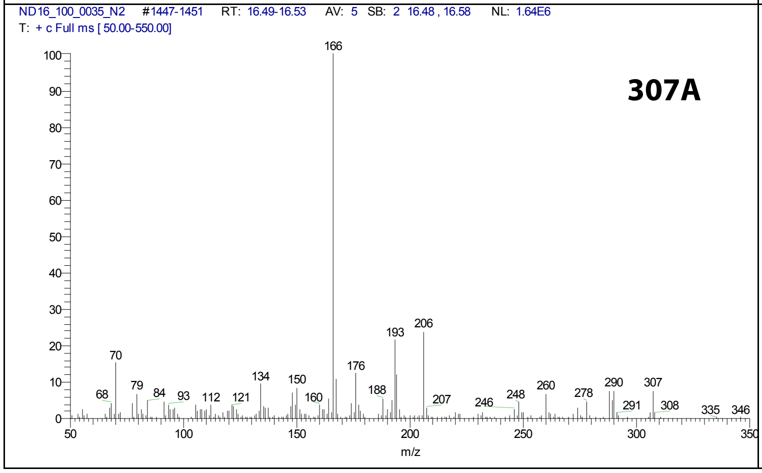
ND16_100_0035_N2 #1447-1451 RT: 16.49-16.53 AV: 5 SB: 2 16.48 , 16.58 NL: 1.64E6
T: + c Full ms [50.00-550.00]
307A
166
193 206
70
68 79 84 93 112 121 134 150 160 176 188 207 246 248 260 278 290 291 307 308 335 346
m/z

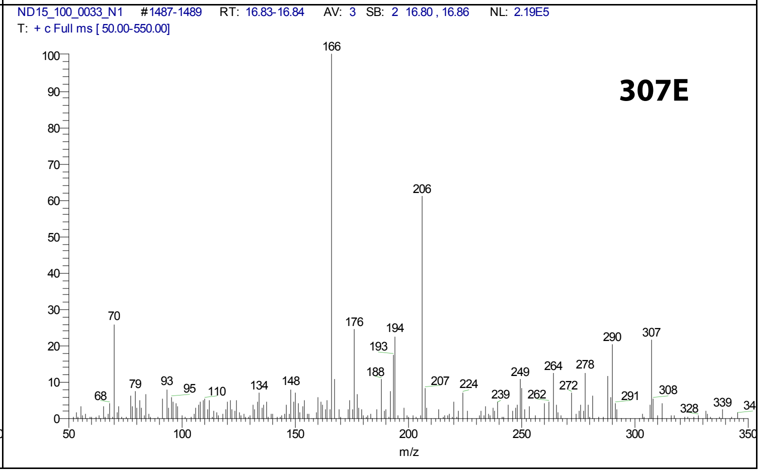
ND15_100_0033_N1 #1487-1489 RT: 16.83-16.84 AV: 3 SB: 2 16.80 , 16.86 NL: 2.19E5
T: + c Full ms [50.00-550.00]
307E
166
206
70
176 194
193
290 307
68 79 93 95 110 134 148 188 207 224 239 249 262 264 272 278 291 308 328 339 34
m/z
