# Supplementary material for: Alkaloids from single skins of the Argentinian toad Melanophryniscus rubriventris (ANURA, BUFONIDAE): An unexpected variability in alkaloid profiles and a profusion of new structures
Source: Springerplus. 2012 Nov 23;1(1):51. doi: 10.1186/2193-1801-1-51 (PMC3625416; doi:10.1186/2193-1801-1-51)
Supplement: Supplementary file 4 — Additional fle 3 Figures S1-S10.: Total mass spectral ion current chromatograms for the alkaloid extracts of toad skin samples #1-10. (ZIP 12984 kb) (ZIP 9566 kb) (ZIP 13 MB) [file 40064_2012_198_MOESM4_ESM.zip › add3/1118854145799791_fig25.pdf]

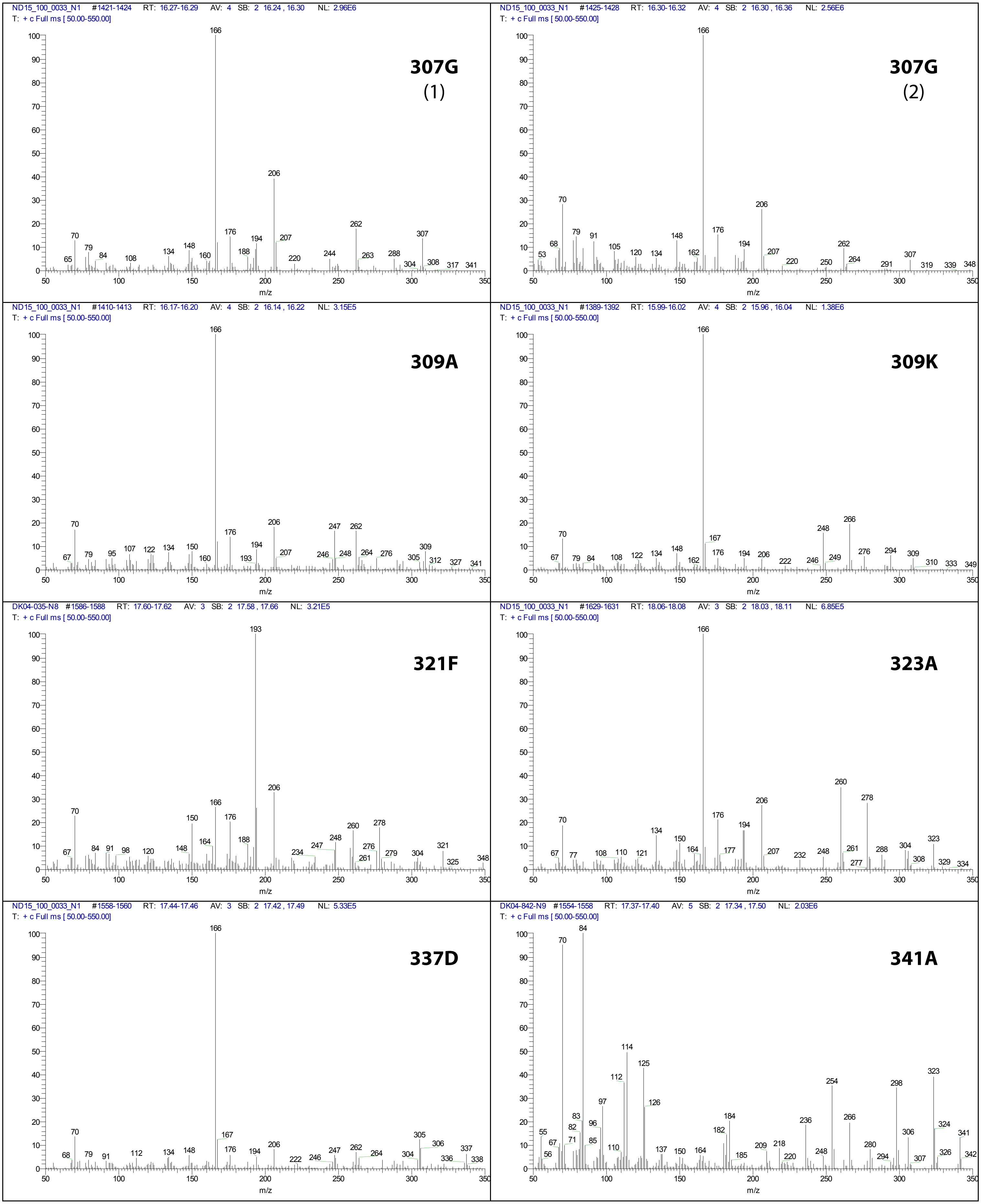
ND15_100_0033_N1 #1421-1424 RT: 16.27-16.29 AV: 4 SB: 2 16.24 , 16.30 NL: 2.96E6
T: + c Full ms [50.00-550.00]
307G (1)
ND15_100_0033_N1 #1425-1428 RT: 16.30-16.32 AV: 4 SB: 2 16.30 , 16.36 NL: 2.56E6
T: + c Full ms [50.00-550.00]
307G (2)
ND15_100_0033_N1 #1410-1413 RT: 16.17-16.20 AV: 4 SB: 2 16.14 , 16.22 NL: 3.15E5
T: + c Full ms [50.00-550.00]
309A
ND15_100_0033_N1 #1389-1392 RT: 15.99-16.02 AV: 4 SB: 2 15.96 , 16.04 NL: 1.38E6
T: + c Full ms [50.00-550.00]
309K
DK04-035-N8 #1586-1588 RT: 17.60-17.62 AV: 3 SB: 2 17.58 , 17.66 NL: 3.21E5
T: + c Full ms [50.00-550.00]
321F
ND15_100_0033_N1 #1629-1631 RT: 18.06-18.08 AV: 3 SB: 2 18.03 , 18.11 NL: 6.85E5
T: + c Full ms [50.00-550.00]
323A
ND15_100_0033_N1 #1558-1560 RT: 17.44-17.46 AV: 3 SB: 2 17.42 , 17.49 NL: 5.33E5
T: + c Full ms [50.00-550.00]
337D
DK04-842-N9 #1554-1558 RT: 17.37-17.40 AV: 5 SB: 2 17.34 , 17.50 NL: 2.03E6
T: + c Full ms [50.00-550.00]
341A
m/z
