# Supplementary material for: Alkaloids from single skins of the Argentinian toad Melanophryniscus rubriventris (ANURA, BUFONIDAE): An unexpected variability in alkaloid profiles and a profusion of new structures
Source: Springerplus. 2012 Nov 23;1(1):51. doi: 10.1186/2193-1801-1-51 (PMC3625416; doi:10.1186/2193-1801-1-51)
Supplement: Supplementary file 4 — Additional fle 3 Figures S1-S10.: Total mass spectral ion current chromatograms for the alkaloid extracts of toad skin samples #1-10. (ZIP 12984 kb) (ZIP 9566 kb) (ZIP 13 MB) [file 40064_2012_198_MOESM4_ESM.zip › add3/1118854145799791_fig26.pdf]

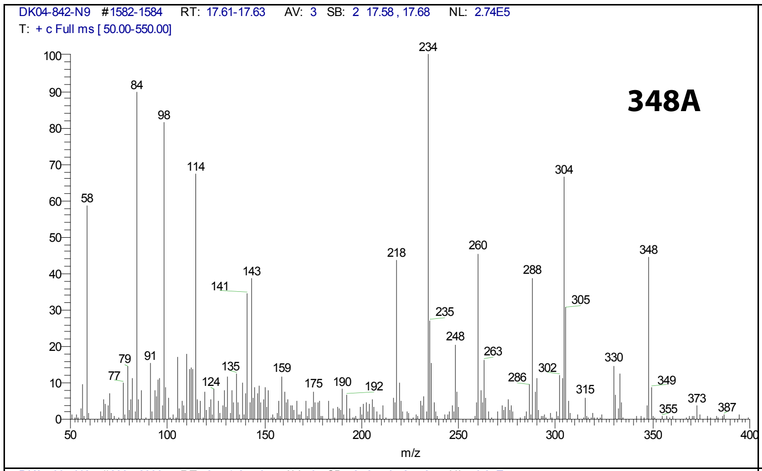
DK04-842-N9 #1582-1584 RT: 17.61-17.63 AV: 3 SB: 2 17.58 , 17.68 NL: 2.74E5
T: + c Full ms [50.00-550.00]
348A
234 84 98 114 304 58 348 260 218 288 143 141 235 305 248 330 263 79 91 159 135 286 302 175 190 192 124 77 349 315 373 387 355
m/z

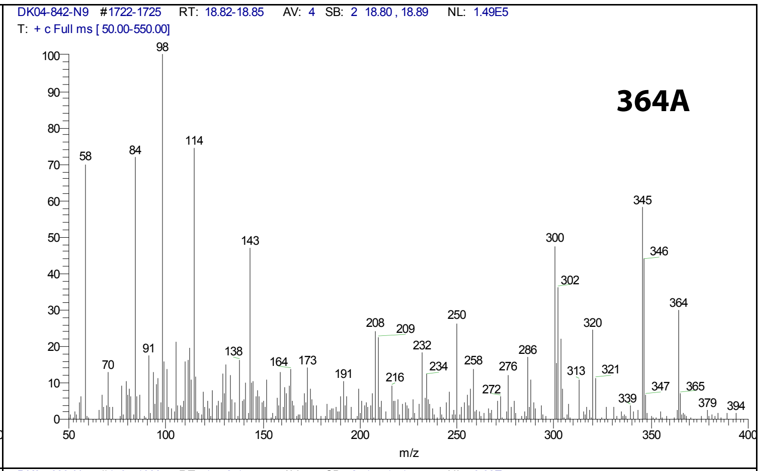
DK04-842-N9 #1722-1725 RT: 18.82-18.85 AV: 4 SB: 2 18.80 , 18.89 NL: 1.49E5
T: + c Full ms [50.00-550.00]
364A
98 114 58 84 345 346 143 300 302 364 208 209 250 320 232 286 91 138 164 173 70 258 276 234 191 216 313 321 272 339 347 365 379 394
m/z

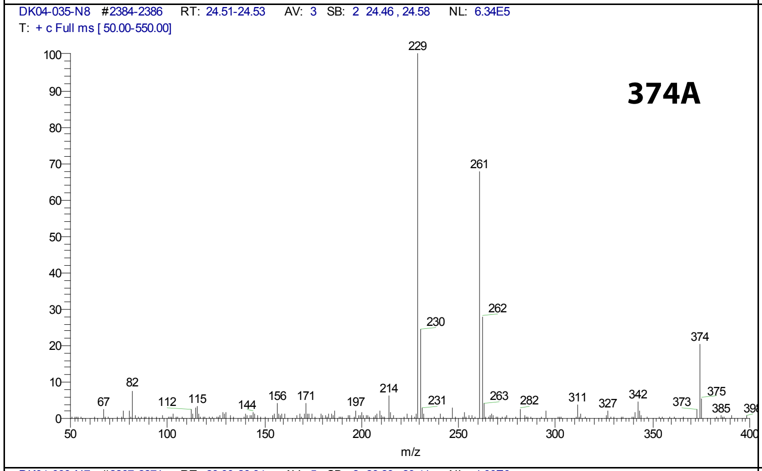
DK04-035-N8 #2384-2386 RT: 24.51-24.53 AV: 3 SB: 2 24.46 , 24.58 NL: 6.34E5
T: + c Full ms [50.00-550.00]
374A
229 261 230 262 374 82 214 375 67 112 115 144 156 171 197 231 263 282 311 327 342 373 385
m/z

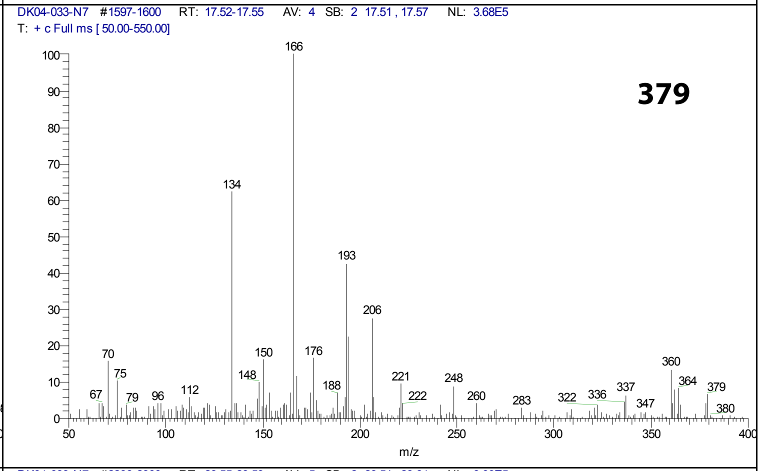
DK04-033-N7 #1597-1600 RT: 17.52-17.55 AV: 4 SB: 2 17.51 , 17.57 NL: 3.68E5
T: + c Full ms [50.00-550.00]
379
166 134 193 206 70 150 176 75 148 112 221 248 67 79 96 188 222 260 283 322 336 337 347 360 364 379 380
m/z

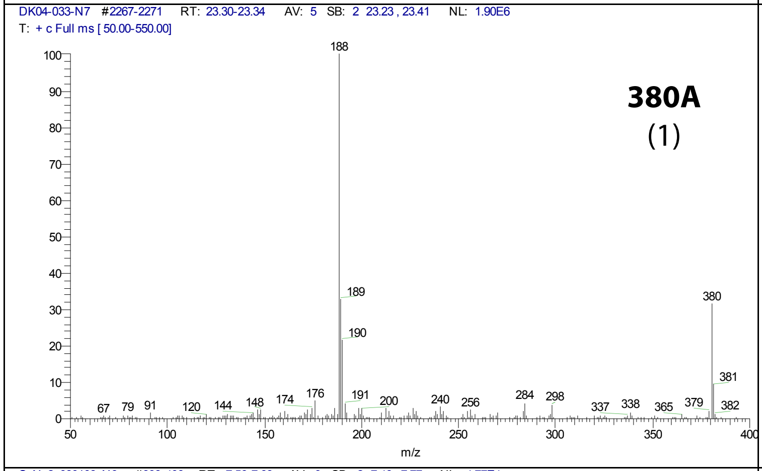
DK04-033-N7 #2267-2271 RT: 23.30-23.34 AV: 5 SB: 2 23.23 , 23.41 NL: 1.90E6
T: + c Full ms [50.00-550.00]
380A
(1)
188 189 190 380 381 176 191 200 240 256 284 298 67 79 91 120 144 148 174 337 338 365 379 382
m/z

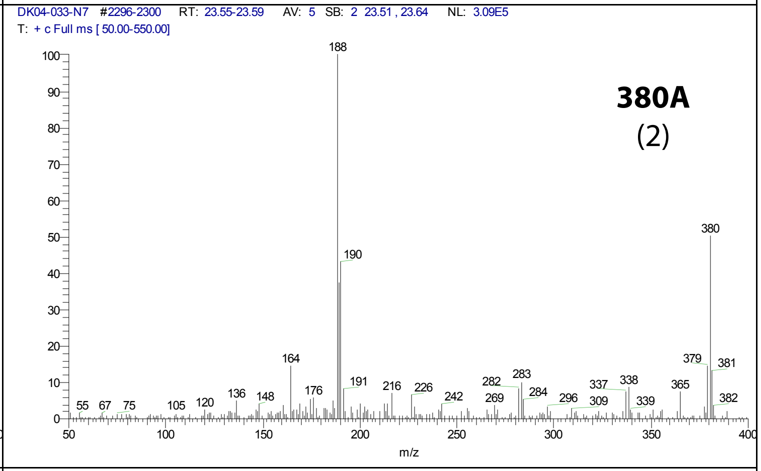
DK04-033-N7 #2296-2300 RT: 23.55-23.59 AV: 5 SB: 2 23.51 , 23.64 NL: 3.09E5
T: + c Full ms [50.00-550.00]
380A
(2)
188 380 190 164 283 282 379 381 191 216 226 136 148 176 242 269 284 296 309 337 338 339 365 382 55 67 75 105 120
m/z

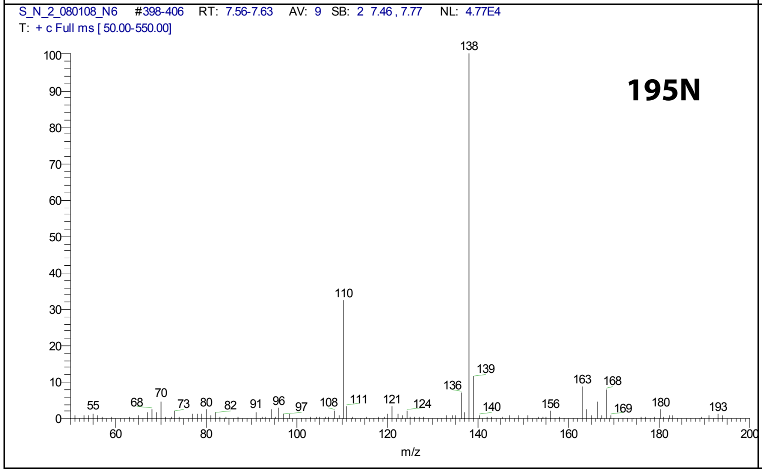
S_N_2_080108_N6 #398-406 RT: 7.56-7.63 AV: 9 SB: 2 7.46 , 7.77 NL: 4.77E4
T: + c Full ms [50.00-550.00]
195N
138 110 139 163 168 136 70 55 68 73 80 82 91 96 97 108 111 121 124 140 156 169 180 193
m/z
